# Muonium Chemistry at Diiron Subsite Analogues of [FeFe]‐Hydrogenase

**DOI:** 10.1002/anie.201607109

**Published:** 2016-10-14

**Authors:** Joseph A. Wright, Jamie N. T. Peck, Stephen P. Cottrell, Aušra Jablonskytė, Vasily S. Oganesyan, Christopher J. Pickett, Upali A. Jayasooriya

**Affiliations:** ^1^ Energy Materials LaboratorySchool of ChemistryUniversity of East AngliaNorwichNR4 7TJUK; ^2^ Rutherford Appleton LaboratoryHarwell OxfordDidcotOX11 0QXUK

**Keywords:** enzyme mimics, [FeFe]-hydrogenase, hydrogen, muonium

## Abstract

The chemistry of metal hydrides is implicated in a range of catalytic processes at metal centers. Gaining insight into the formation of such sites by protonation and/or electronation is therefore of significant value in fully exploiting the potential of such systems. Here, we show that the muonium radical (Mu^.^), used as a low isotopic mass analogue of hydrogen, can be exploited to probe the early stages of hydride formation at metal centers. Mu^.^ undergoes the same chemical reactions as H^.^ and can be directly observed due to its short lifetime (in the microseconds) and unique breakdown signature. By implanting Mu^.^ into three models of the [FeFe]‐hydrogenase active site we have been able to detect key muoniated intermediates of direct relevance to the hydride chemistry of these systems.

Developing new approaches to gaining insight into catalytic systems is of central importance. We are now exploring the exciting possibility of using muonium radicals as surrogates for H^.^ in the study of catalytic and electrocatalytic reactivity at metal centers. The system we have chosen is the active site of the [FeFe]‐hydrogenase.

The [FeFe]‐ and [NiFe]‐hydrogenases catalyze the reversible reduction of protons to dihydrogen at high turnover frequencies and at low overpotentials. On a per catalytic site basis, the turnover frequencies of certain of these enzymes immobilized on electrodes can rival the best conventional electrocatalyst, carbon‐supported platinum.^1^, ^2^, ^3^ However, the high molecular mass and large geometric footprint of the native enzymes result in rather low current densities, at best ca. 3 mA cm^−2^ at 20 °C. Given that hydrogen fuel or producer cells typically operate at current densities some two or three orders of magnitude greater, it is arguable whether hydrogenase‐based electrodes are likely to be useful materials in practical devices. Nevertheless, it is well established that the relatively small metallosulfur centers within these enzymes are responsible for their high catalytic activity and this has prompted extensive research on synthetic analogues of these active sites.^1^, ^3^, ^4^ This research is driven both by the need to provide mechanistic insights into the biological chemistry and the possibility of creating new materials for electrocatalysis based on abundant 3d metals.

Mechanistically, the formation of more or less transient hydride intermediates is central to both hydrogen evolution or uptake by the [FeFe]‐ and [NiFe]‐hydrogenases and electrocatalysis by synthetic analogues of their active sites.^5^ We now describe the first application of muon spin spectroscopy to probe hydride chemistry at metallosulfur sites related to that within a hydrogenase, specifically the diiron subsite of [FeFe]‐hydrogenase.

When an energetic muon (μ^+^) beam passes through a solid sample some of the sub‐atomic μ^+^ ions capture an electron to form muonium radicals (Mu^.^);^6^ these are sufficiently slowed to react with the bulk target species, in our case the diiron subsite analogue. Muonium can be considered chemically as acting as a light isotope of hydrogen atom, having about 1/9 of its mass; Mu^.^ thus provides a surrogate for hydrogen radicals. The implantation of a muonium radical on a diiron‐subsite analogue can be viewed as the equivalent of concerted addition of a proton and an electron; it takes place on the nano‐ to microsecond timescale giving rise to paramagnetic species.^6^ Analogous to the proton, the muon has a nuclear spin of one‐half; at the end of its life (lifetime 2.197 μs) it decays to give a positron which is emitted preferentially along the spin direction at the moment of decay. Because it is possible to produce almost 100 % spin‐polarized muon beams, detection of the direction of emission of the decay positrons allows the study of the evolution of the muon(ium) spin within the implanted sample. This gives information on the system under scrutiny via the magnetic fields local to the muon site, μSR (muon spin rotation/relaxation/resonance) spectroscopy.^7^

To date, there have been only a very small number of studies on application of μSR to organometallic systems.^8^ These have been focused on silylenes,^9^ or cyclopentadienyl or arene (half)sandwich systems;^10^, ^11^, ^12^, ^13^ in these compounds protonation and proton‐coupled electron transfer are of limited relevance to the key chemical reactions of the metal complexes. In contrast, the spectroscopic data we have so obtained can be modelled by ab initio DFT calculations and we show that this provides compelling evidence for the muono‐formyl Fe‐C(Mu)=O and bridging Fe‐Mu‐Fe or terminal Fe‐Mu muonide transients, light isotopes of paramagnetic species that are implicit in hydrogen evolution and uptake.^1^, ^2^, ^3^, ^14^

As far as we are aware, there are no studies using μSR spectroscopy to probe chemical processes relevant to metal hydrides in catalysis or electrocatalysis. In this work, we have examined three [FeFe]‐hydrogenase subsite models, each of which illustrate a different aspect of hydride chemistry (Figure 1). Thus, complex **1** is known to engage in electrocatalysis, in which an electron and a proton are added successively;^15^ complex **2** protonates at the metal–metal bond^16^ enabling subsequent electronation to yield a mixed‐valence Fe(1.5)Fe(1.5) hydride (cf. H‐atom addition);^17^ whilst complex **3** possesses the bis‐cyanide coordination found in the enzyme and has been shown to reconstitute an apoenzyme.^18^, ^19^, ^20^

**Figure 1:**
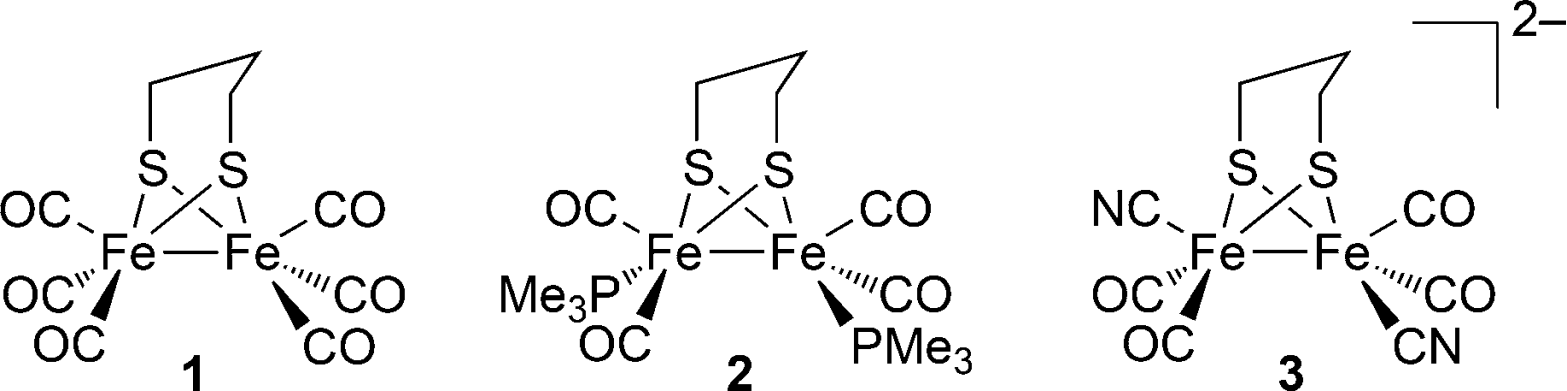
Complexes **1**–**3**.

In the solid state (an anisotropic environment), coupling between the radical (electron) and muon spins gives rise to the Δ_1_ transition.^6^ This may be probed using “avoided level crossing muon spin resonance” (ALC‐μSR).^6^ ALC is a longitudinal field μSR technique which detects the reduction in polarization at “level crossings” in the Breit–Rabi diagram. For Δ_1_ resonances, where only the muon spin changes sign, the resonance field is related to the hyperfine interaction by equation (1).

(1) $$B_{\mathrm{res}}^{\Delta_{1}}=\frac{1}{2}\left[\frac{A_{\mu }}{\gamma_{\mu }}-\frac{A_{\mu }}{\gamma_{e}}\right]$$

ALC‐μSR spectra of **1**–**3** at 300 K over the field range 2 kG to 18 kG are shown in Figure 2.^21^ The general features of all three spectra are remarkably similar: each room temperature spectrum exhibits a broad signal with a maximum in the range 8 kG to 10 kG. The signals all show significant temperature dependence, with essentially complete loss of the signal at the lowest temperature (10 K) for **1** and **3**, whilst **2** exhibits only a small residual signal.

**Figure 2:**
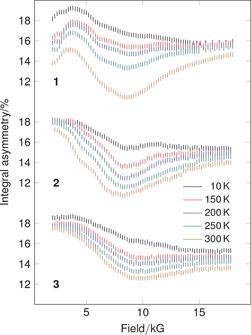
Raw time‐integral ALC‐μSR spectra for **1**–**3**. Data points are shown as sticks representing the estimated uncertainty in each point. The vertical scale is the same for each spectrum.

Given the very broad nature of the ALC‐μSR signals, accurate modelling of the background is essential. Scanning using a cell containing a mass of aluminum foil equivalent to the sample showed a smooth curve which could be fitted using a fourth‐order polynomial (Figure S1). Whilst the line shape for the solid‐state anisotropic signals here is expected to be complex, it is possible to approximate the spectra using Gaussian curves following subtraction of the cell background (Figure 3, Figures S5, S6). This process confirms the presence of a common major signal at around 8.5 kG in all three samples. A second maximum at ca. 4.5 kG can clearly be seen at 300 K for **2** and at lower temperatures for **1** (Figure 2, Figures S5, S6). Complex **3** does not show this signal.

**Figure 3:**
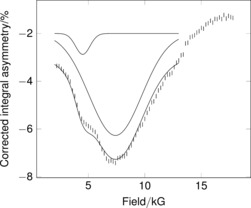
Background‐subtracted time‐integral ALC‐μSR spectra for **2** at 300 K showing Gaussian line shape approximation for the range 2 kG to 13 kG.

The specific nature of the muonium species which give rise to the ca. 8.5 kG and ca. 4.5 kG signals has been probed by computational simulation of plausible structures, from which Δ_0_ values have been calculated.^22^ For the known solid‐state structure of **2** which has a basal–basal deployment of phosphine ligands,^16^ the high‐field signal at 8.5 kG is consistent with the Mu being bound to iron either in a bridging or terminal position (Figure 4). For **3**, only the bridging muonide fits with the experimental value. The low‐field signal at 4.5 kG can be accounted for by formation of a formyl‐like radical (Figure 5, left). We note that structures in which the rotation of a tripodal ligand group has occurred can also accommodate the observed signals (Figure 5, right), but such a rotation is perhaps unlikely in the solid state given the timeframe of the ALC‐μSR experiment.

**Figure 4:**
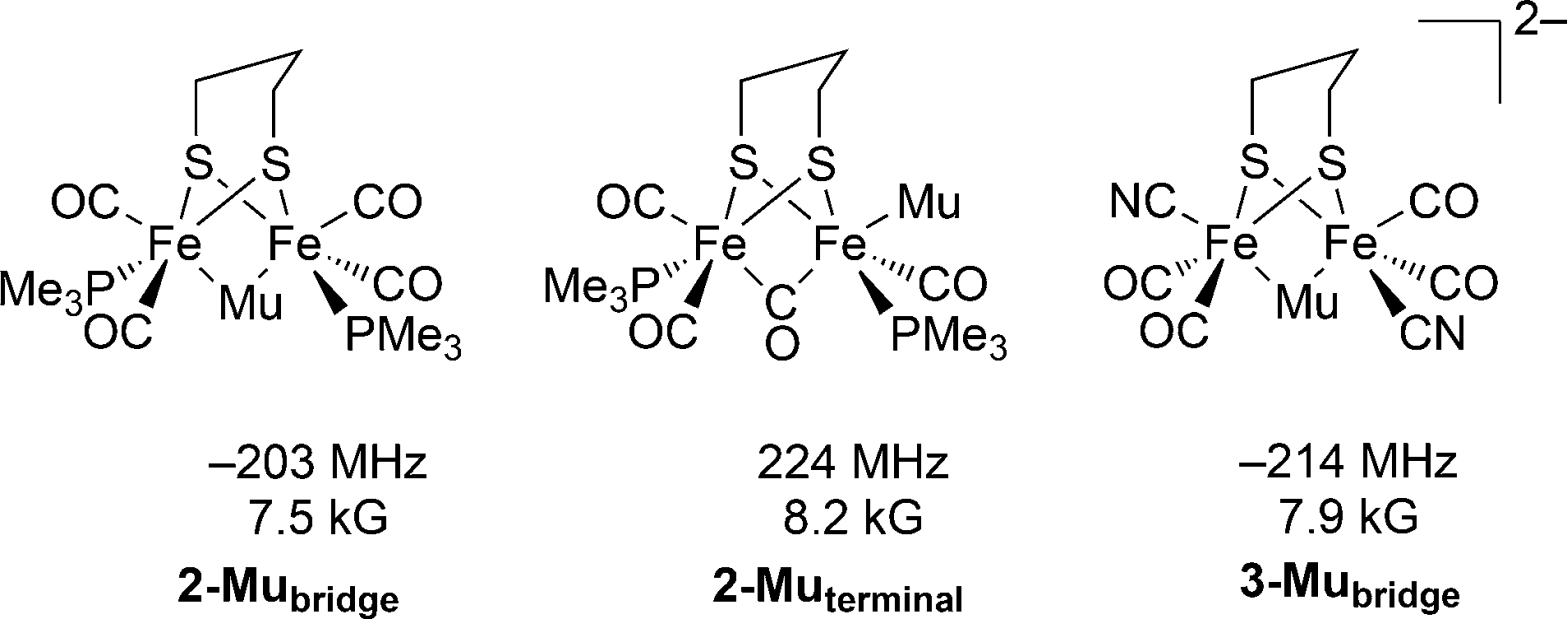
Potential sites of muon addition, and calculated hyperfine coupling and resonance field values.

**Figure 5:**
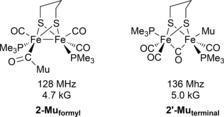
Example sites with resonance fields of around 5.0 kG.

It is important to note that **2‐Mu_bridge_** is structurally analogous to the mixed‐valence bridging hydride detected by electron paramagnetic resonance on one‐electron reduction of the closed‐shell cationic hydride, [HFe_2_(pdt)(CO)_4_(PMe_3_)_2_]^+^,^17^ and that **1‐Mu_formyl_** can be regarded as an isotopomer of the formyl species observed upon reduction of **1** in presence of acid.^6^, ^15^

Figure 2 shows that the intensities of the signals are temperature‐dependent. At low temperature (10 K) the response approaches that of the cell background. Figure 6 shows the Arrhenius plot for the major resonance in each of the three samples where the 10 K data is subtracted with the spectra pinned to zero at 14 kG (**1**)/18 kG (**2** and **3**). For the full range of data **2** and **3** there is an excellent linear correlation of ln *I* with 1/*T*.^23^ The more limited data for **1** shows a similar trend. The estimated activation energies obtained for the addition at the bridging position are **1** 3.40(2) kJ mol^−1^, **2** 2.45(2) kJ mol^−1^ and **3** 1.58(3) kJ mol^−1^, consistent with the high reactivity of muonium. The order obtained is notable, with activation energies falling as the systems become more electron rich: **1** is the least electron rich, **3** the most.

**Figure 6:**
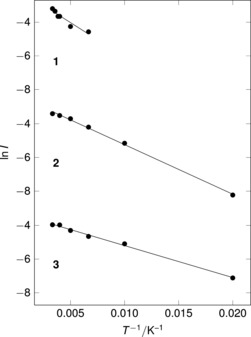
Arrhenius plots for signal intensity following muoniation, subtracting 10 K spectra and correcting for temperature‐dependent drift. (Resonance positions: **1** 8.4 kG, **2** 8.2 kG, **3** 10.0 kG).

The second resonance which we have assigned to **Mu_formyl_** in the data for the PMe_3_ complex (**2**) remains resolved across the temperature range 100 K to 300 K. From the raw ln *I* and 1/*T* data we estimate an activation energy of ca. 9 kJ mol^−1^ from the Arrhenius relationship (correlation coefficient 0.965). In the lower temperature scans, the intensity of the resonance is lost in the background.

As a further verification that radical states are formed, repolarization experiments were also carried out (Figure 7, Figure S14). We clearly observed the recovery of polarization across the temperature range. Whilst such experiments confirm radical generation, extraction of hyperfine coupling constants is known to be difficult.^6^ It is notable however that there is recovery at the lowest temperature (5 K), where the ALC‐μSR spectra are essentially featureless. Thus, radical states are still being formed at low temperature but likely broadened beyond detection in the ALC‐μSR. This may be due to the existence of multiple sites of addition, consistent with the DFT calculations, and/or may be due to electron‐spin relaxation.

**Figure 7:**
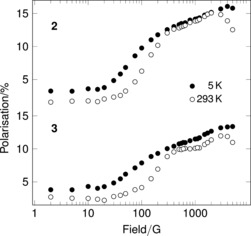
Repolarization spectra for **2** and **3** at ambient and base temperatures. Estimated uncertainty values were smaller than the markers used for all points. The vertical scale is the same for both spectra.

In summary, we have provided evidence that muonium interacts in the solid state with the diiron subsite analogues **1**–**3** to give 35‐electron bridging muonides (μ‐Mu)Fe_2_(pdt)(CO)_4_(L)_2_. The formation of these species is temperature‐dependent with activation energies less than 4 kJ mol^−1^. In the case L=PMe_3_ (**2**) we observed a second resonance, assigned to the formation of a muono‐formyl species, ‐COMu. The generation of these muoniated species have direct parallels in the protonation/electronation of substrates^17^ and represent the first example of the application of μSR to electrocatalytic systems.

The work described here is likely to presage a wider application of muonium chemistry. The role of metal–hydride interactions in diverse inorganic, organometallic, and biological chemistry is extensive, ranging from β‐elimination through water‐gas shift chemistry, the chemistry of the hydrogenases and nitrogenases, to electrocatalytic systems for hydrogen fuel/producer cells. Muonium chemistry coupled with the fast timescale of μSR spectroscopy offers the prospect of unravelling mechanistic detail of such systems, for example, the possibility of detecting transient dihydrogen/dihydride analogues.

## Supporting information

As a service to our authors and readers, this journal provides supporting information supplied by the authors. Such materials are peer reviewed and may be re‐organized for online delivery, but are not copy‐edited or typeset. Technical support issues arising from supporting information (other than missing files) should be addressed to the authors.

Supplementary
