# Supplementary material for: Muonium Chemistry at Diiron Subsite Analogues of [FeFe]‐Hydrogenase
Source: Angew Chem Int Ed Engl. 2016 Oct 14;55(47):14580–3. doi: 10.1002/anie.201607109 (PMC5484327; doi:10.1002/anie.201607109)
Supplement: Supplementary file 1 — Supplementary [file ANIE-55-14580-s001.pdf]

Angewandte Chemie
Eine Zeitschrift der Gesellschaft Deutscher Chemiker

Supporting Information

Muonium Chemistry at Diiron Subsite Analogues of [FeFe]-Hydrogenase

Joseph A. Wright, Jamie N. T. Peck, Stephen P. Cottrell, Aušra Jablonskytė, Vasily S. Oganesyan, Christopher J. Pickett,* and Upali A. Jayasooriya**

anie_201607109_sm_miscellaneous_information.pdf

Experimental

The compounds **1**–**3** were prepared by literature methods.[S1–S3] Avoided level crossing muon experiments were performed on the HiFi spectrometer at the ISIS pulsed muon facility at the STFC Rutherford Appleton Laboratory, UK. Repolarization experiments were performed on the HiFi spectrometer (**1**) or the EMU spectrometer (**2** and **3**) at the same facility. The sample environment was provided by a closed cycle refrigerator cryostat. Samples were packed into titanium sample cells (25 mm diameter). Ten million events were collected for each data point. Background scans were performed using a cell filled with aluminum foil of the same mass as the sample and mounted in an identical fashion. Data were processed using the Mantid program.[S4]

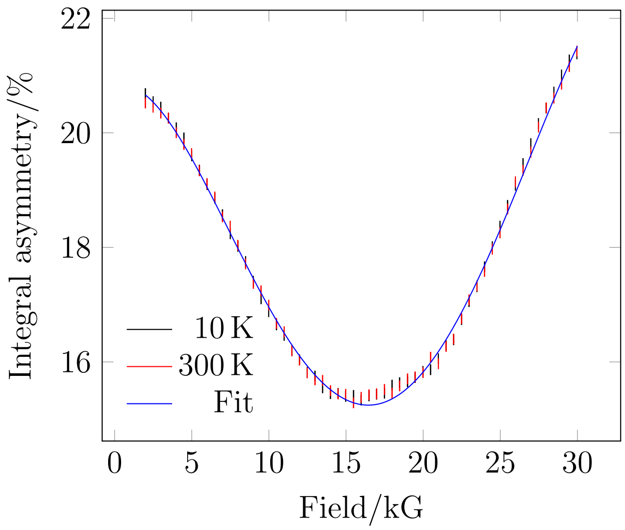

Figure S1: Time-integral ALC-SR background spectra (Al foil). Real data are shown as sticks representing the estimated uncertainty in each point. The line is a fourth-order polynomial fit using data for both temperatures: fitting the two data sets independently gave fit parameters identical within the estimated uncertainty values.

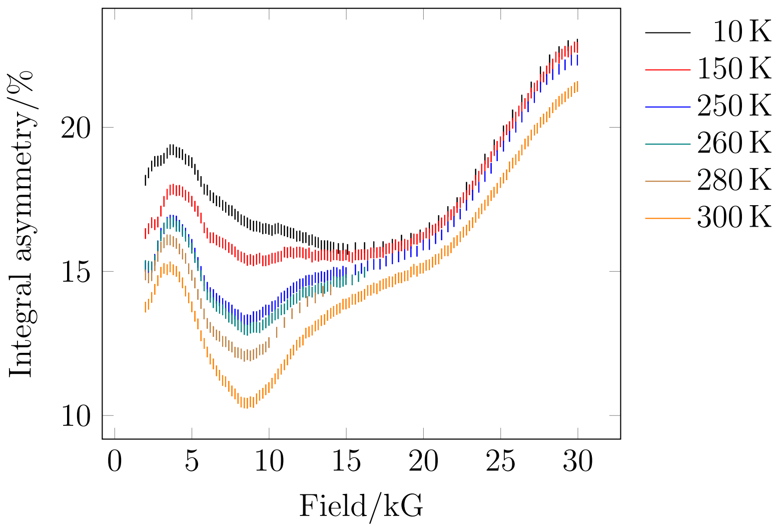

Figure S2: Raw time-integral ALC-SR background spectrum of **1** at all temperatures measured.

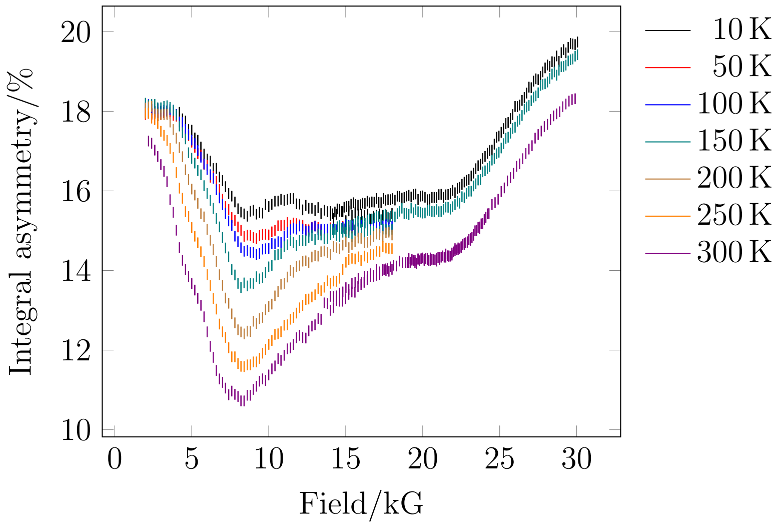

Figure S3: Raw time-integral ALC-SR background spectrum of **2** at all temperatures measured. Note that the region 15 kG to 30 kG was measured in separate runs to the region 2 kG to 18 kG, resulting in the overlapping data points and some discontinuity in the overlapping region.

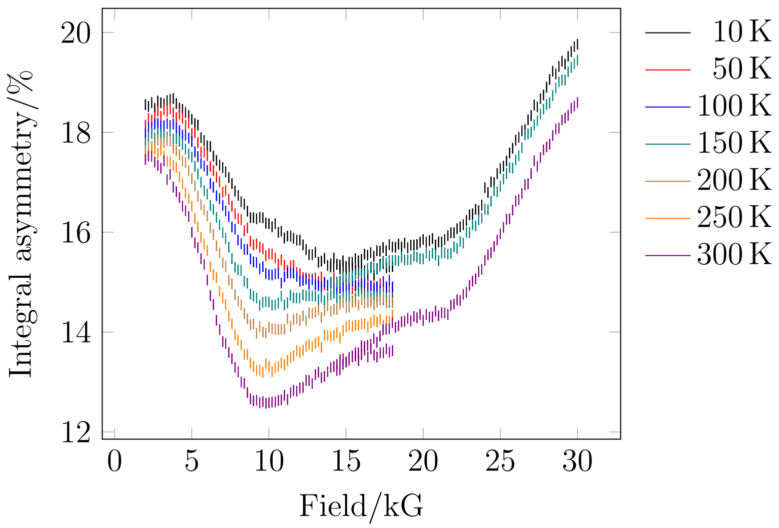

Figure S4: Raw time-integral ALC-SR background spectrum of **3** at all temperatures measured. Not that the region 15 kG to 30 kG was measured in separate runs to the region 2 kG to 18 kG, resulting in the overlapping data points and some discontinuity in the overlapping region.

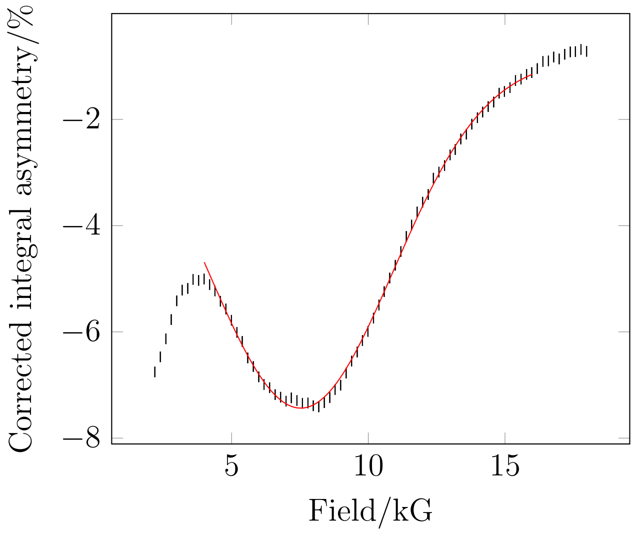

Figure S5: Background-subtracted time-integral ALC-SR spectra for **1** at 300 K showing Gaussian line shape approximation for the range 4 kG to 16 kG.

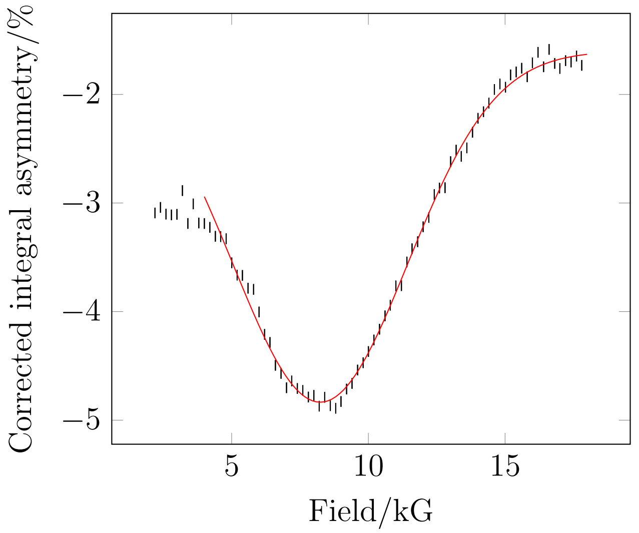

Figure S6: Background-subtracted time-integral ALC-SR spectra for **3** at 300 K showing Gaussian line shape approximation for the range 4 kG to 18 kG.

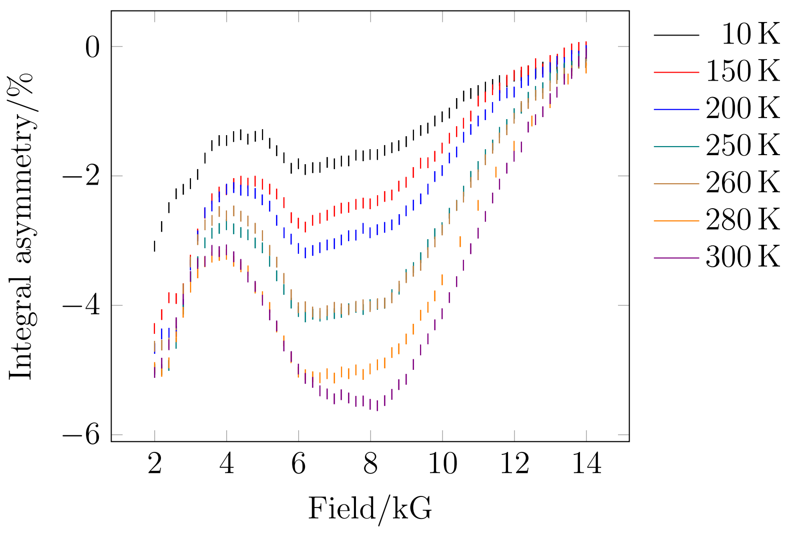

Figure S7: Background-subtracted time-integral ALC-SR spectra for **1** offset such that 14 kG data point is at constant (zero) asymmetry.

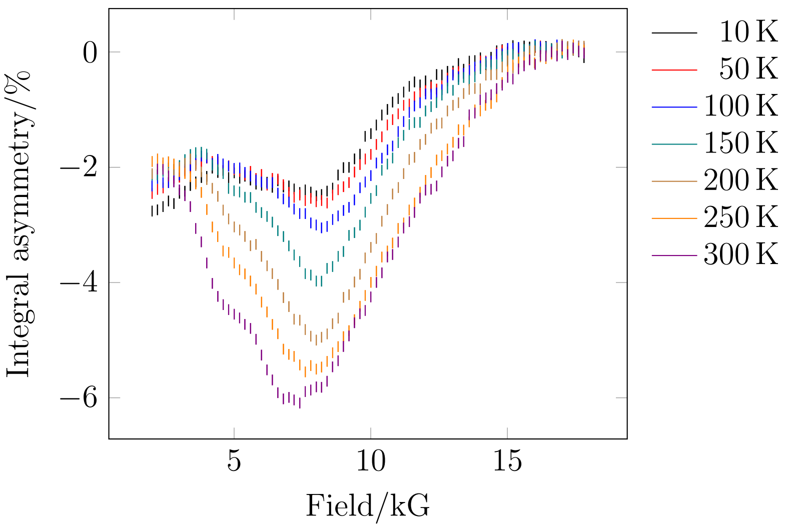

Figure S8: Background-subtracted time-integral ALC-SR spectra for **2** offset such that 18 kG data point is at constant (zero) asymmetry.

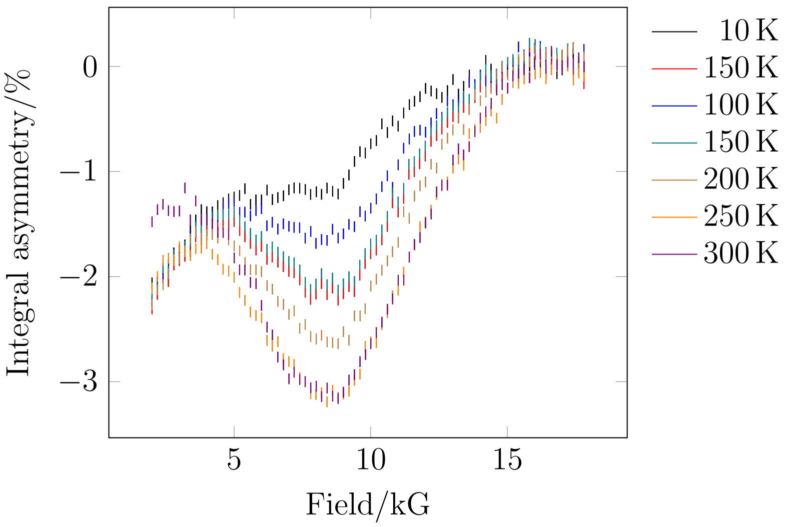

Figure S9: Background-subtracted time-integral ALC-SR spectra for **3** offset such that 18 kG data point is at constant (zero) asymmetry.

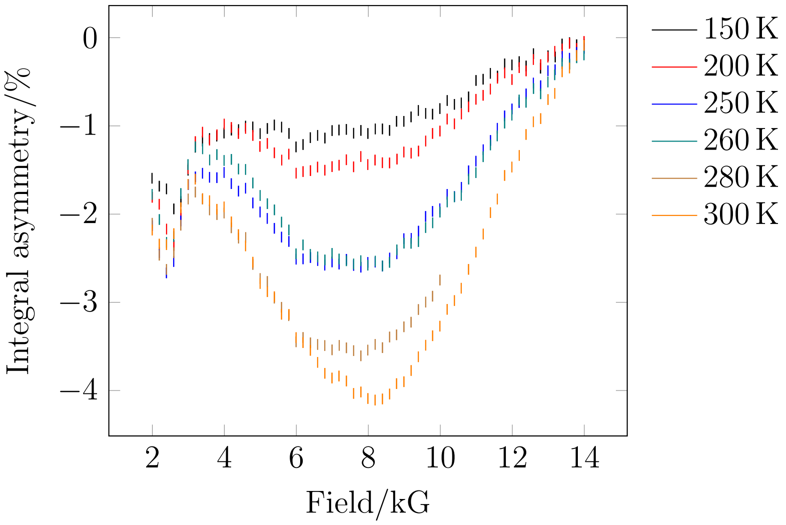

Figure S10: Time-integral ALC-SR spectra for **1** with spectrum at 10 K subtracted and offset such that 14 kG data point is at constant (zero) asymmetry.

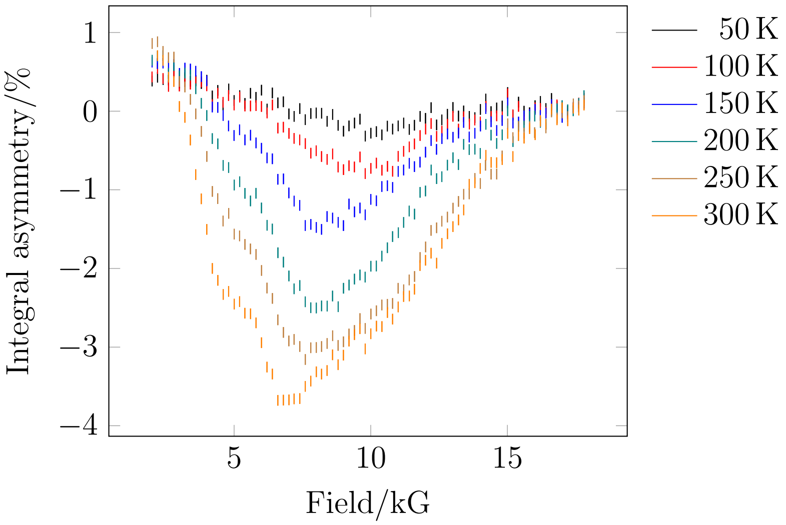

Figure S11: Time-integral ALC-SR spectra with spectrum at 10 K subtracted and offset such that 18 kG data point is at constant (zero) asymmetry.

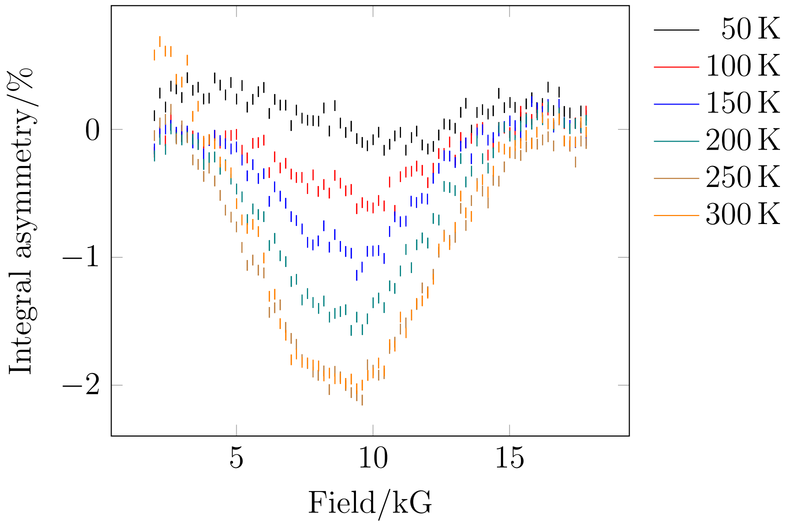

Figure S12: Time-integral ALC-SR spectra for **3** with spectrum at 10 K subtracted and offset such that 18 kG data point is at constant (zero) asymmetry.

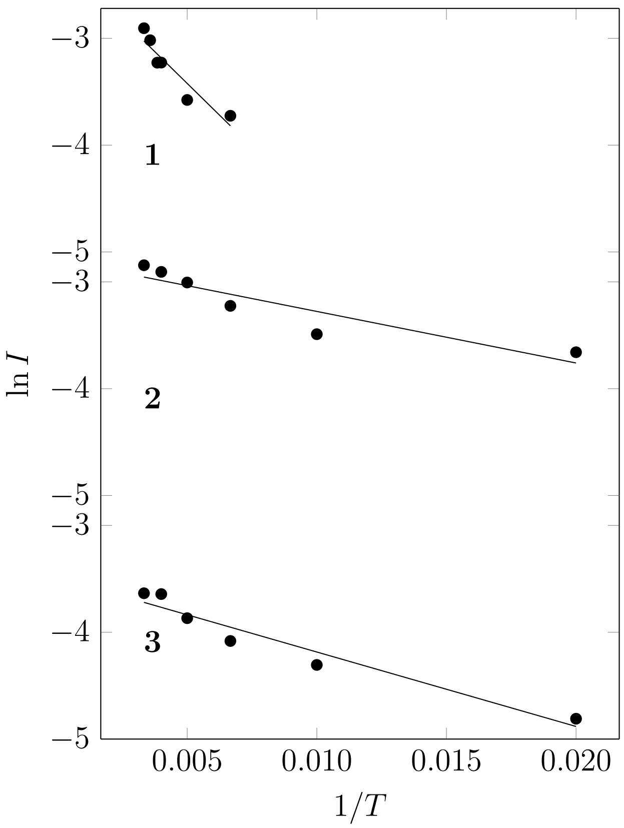

Figure S13: Arrhenius plots for signal intensity following muoniation using baseline-subtracted spectra and correcting for temperature-dependent drift. (Peek positions: **1** 8.4 kG, **2** 8.2 kG, 10.0 kG). Estimated activation enthalpies **1** 2.0 kJ mol^{-1}, **1** 0.4 kJ mol^{-1}, **1** 0.6 kJ mol^{-1}.

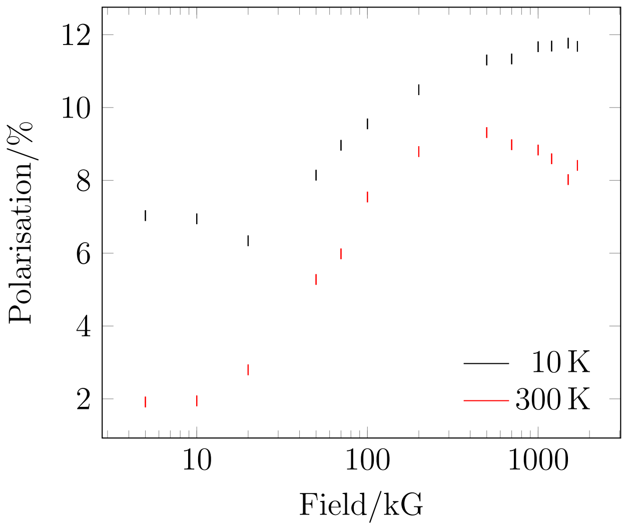

Figure S14: Repolarization spectra for **1** at ambient and base temperatures. Data points are shown as sticks representing the estimated uncertainty in each point.

Density functional theory (DFT) calculations

All calculations were performed using the Gaussian 09[S5] computational package. Geometry optimisation and frequency calculations have been carried out using the Tao–Perdew–Staroverov–Scuseria[S6] (TPSS) density functional. Phosphorus, sulfur and iron atoms are described by the the Hay and Wadt LANL2DZ[S7,S8] basis set with effective core potential (ECP). In the case of iron, the two outermost p functions were replaced with re-optimized 4p functions.[S9] For sulfur and phosphorus, additional p and d polarisation functions were added.[S10] All other atoms employ the all electron 6-31+G** basis set. Structures were geometry optimised in the gas phase with the default convergence criteria and confirmed as minima through frequency calculations.

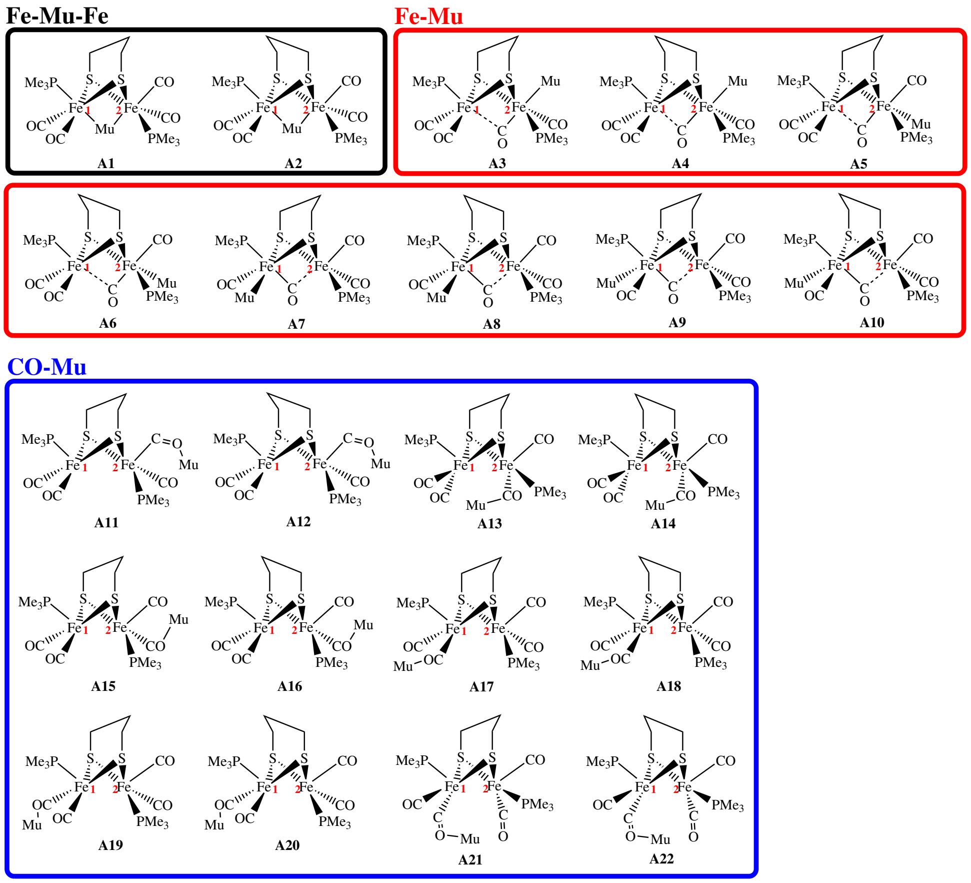

Figure S15: Possible muoniated radicals of the apical/basal structural isomer, **A**, of the $[MuFe_2(pdt)(CO)_4(PMe_3)_2]$ model system.

Table S1: DFT calculated isotropic muon-electron hyperfine coupling constants, $A_{e\mu}$, anisotropic muon hyperfine tensors and associated resonance fields for possible apical/basal isomers of $[MuFe_2(pdt)(CO)_4(PMe_3)_2]$ muoniated radicals. Reference structures are displayed in Figure S15.

Radical	$A_{e\mu}$/MHz	Muon dipole tensor/MHz			$\Delta_1 B_{res}$/T
		A_{aa}	A_{bb}	A_{cc}	
A1	−225.251	−21.977	−2.609	24.586	0.827
A2	−211.351	−21.301	−2.381	23.682	0.776
A3	209.947	−5.34	−4.457	9.797	0.771
A4	135.991	−4.171	−3.068	7.239	0.499
A5	−62.759	−6.733	−4.973	11.706	0.230
A6	−20.770	−4.425	−3.495	7.92	0.076
A7	−83.843	−10.013	−8.247	18.26	0.308
A8	−94.168	−11.000	−9.116	20.116	0.346
A9	−81.131	−10.061	−8.142	18.203	0.298
A10	−91.393	−11.077	−9.013	20.09	0.336
A11	227.974	−2.110	−1.444	3.554	0.837
A12	195.607	−1.892	−1.268	3.16	0.718
A13	2.405	−2.309	−0.763	3.072	0.009
A14	397.137	−3.882	−2.468	6.35	1.458
A15	9.655	−2.205	−0.686	2.891	0.035
A16	117.607	−1.388	−1.077	2.465	0.432
A17	400.715	−4.115	−2.784	6.899	1.472
A18	408.585	−4.050	−3.028	7.078	1.500
A19	434.203	−4.000	−3.134	7.134	1.594
A20	435.135	−4.111	−3.138	7.249	1.598
A21	−20.041	−1.894	−1.166	3.06	0.074
A22	−10.083	−1.804	−1.232	3.036	0.037

Radical	$A_{\mathrm{P1,e\mu}}$/MHz	Muon dipole tensor/MHz			$\Delta_1 B_{\mathrm{res}}$/T	$A_{\mathrm{P2,e\mu}}$/MHz	Muon dipole tensor/MHz			$\Delta_1 B_{\mathrm{res}}$/T
		A_{aa}	A_{bb}	A_{cc}		A_{aa}	A_{bb}	A_{cc}		
A1	230.329	−15.971	−11.624	27.596	−2.451	−39.284	−5.905	2.416	3.489	0.996
A2	256.759	−14.939	−14.259	29.197	−2.519	−36.654	−5.236	2.177	3.059	0.936
A3	−7.770	−0.844	0.302	0.542	1.168	252.613	−17.918	−13.621	31.539	1.405
A4	−0.931	−0.223	0.040	0.183	0.734	238.016	−15.960	−14.923	30.883	1.290
A5	−20.067	−3.188	1.157	2.031	0.228	257.014	−17.772	−13.840	31.612	1.495
A6	−5.037	−1.050	0.260	0.790	0.084	235.349	−15.885	−14.716	30.602	1.297
A7	−33.922	−5.997	2.714	3.283	0.266	194.651	−17.041	−11.610	28.651	1.233
A8	−29.622	−5.274	2.380	2.893	0.345	219.647	−16.737	−15.230	31.967	1.345
A9	−29.296	−5.679	2.219	3.460	0.277	198.641	−17.286	−11.476	28.762	1.229
A10	−29.029	−5.221	2.121	3.100	0.333	222.771	−16.901	−15.120	32.021	1.358
A11	138.562	−11.986	−8.390	20.376	0.475	−13.151	−2.057	0.715	1.342	0.814
A12	161.798	−12.337	−10.833	23.169	0.176	−10.459	−1.541	0.496	1.045	0.924
A13	236.364	−17.438	−12.856	30.293	1.263	−7.847	−1.445	0.441	1.004	1.310
A14	48.846	−4.310	−3.284	7.594	1.866	129.746	−9.925	−7.995	17.919	−0.438
A15	244.292	−16.110	−14.801	30.911	1.267	−6.257	−1.169	0.331	0.838	1.344
A16	209.043	−14.858	−13.140	27.998	0.498	33.785	−1.979	−1.523	3.501	0.939
A17	−13.970	−2.156	0.300	1.856	2.224	8.075	−1.006	−0.255	1.261	−0.119
A18	−8.428	−1.366	0.093	1.274	2.237	4.543	−0.899	0.031	0.868	−0.070
A19	−10.361	−1.792	0.169	1.623	2.384	9.072	−0.798	−0.505	1.303	−0.105
A20	−10.709	−1.619	0.239	1.380	2.391	7.340	−0.703	−0.458	1.161	0.097
A21	187.488	−15.475	−9.481	24.956	1.120	−40.593	−5.794	2.454	3.339	1.225
A22	215.841	−14.408	−12.716	27.124	−1.219	−34.942	−4.912	1.972	2.940	1.346

Table S2: DFT calculated phosphorus–electron hfccs, $A_{e\mu}$, and associated resonance fields for possible apical/basal isomers of $[MuFe_2(pdt)(CO)_4(PMe_3)_2]$ muoniated radicals. Reference structures are displayed in Figure S15.

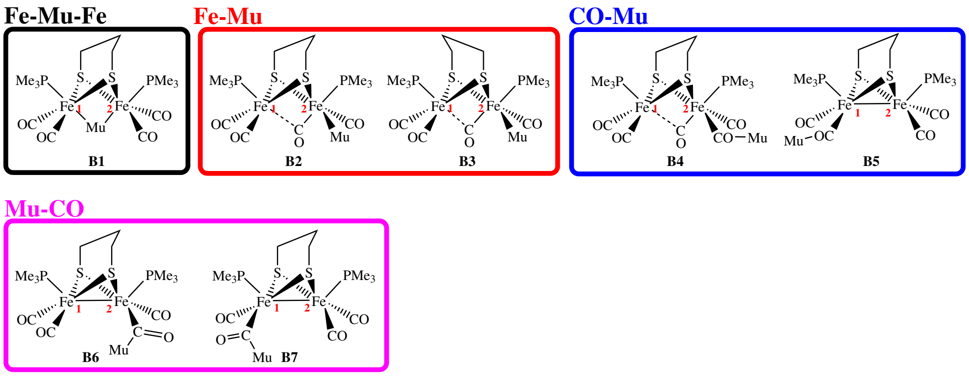

Figure S16: Possible muoniated radicals of the apical/apical structural isomer, **B**, of the [$MuFe_2(pdt)(CO)_4(PMe_3)_2$] model system.

Table S3: DFT calculated isotropic muon-electron hyperfine coupling constants, $A_{e\mu}$, anisotropic muon hyperfine tensors and associated resonance fields for possible apical/apical isomers of [$MuFe_2(pdt)(CO)_4(PMe_3)_2$] muoniated radicals. Reference structures are displayed in Figure S16.

Radical	$A_{e\mu}$/MHz	Muon dipole tensor/MHz			$\Delta_1 B_{res}$/T
		A_{aa}	A_{bb}	A_{cc}	
B1	−255.712	−23.996	−2.000	25.995	0.939
B2	−81.110	−9.846	−7.979	17.824	0.298
B3	−65.684	−8.315	−6.710	15.025	0.241
B4	415.763	−4.165	−2.896	7.061	1.527
B5	409.925	−4.175	−2.728	6.903	1.505
B6	293.033	−7.044	−2.622	9.666	1.076
B7	58.016	−4.946	−1.890	6.836	0.213

Radical	$A_{\mathrm{P1},e\mu}$/MHz	Muon dipole tensor/MHz			$\Delta_1 B_{\mathrm{res}}$/T	$A_{\mathrm{P2},e\mu}$/MHz	Muon dipole tensor/MHz			$\Delta_1 B_{\mathrm{res}}$/T
		A_{aa}	A_{bb}	A_{cc}		A_{aa}	A_{bb}	A_{cc}		
B1	188.987	−14.221	−9.996	24.217	2.391	219.134	−13.498	−12.464	25.962	−2.554
B2	174.938	−14.363	−12.232	26.595	1.379	243.786	−17.170	−13.031	30.202	−0.378
B3	147.079	−13.973	−8.904	22.876	1.146	255.375	−15.450	−15.114	30.565	−0.590
B4	−8.170	−1.140	0.418	0.722	2.274	−1.067	−1.405	−0.340	1.745	−0.038
B5	−15.073	−2.074	0.306	1.769	2.280	−0.821	−0.662	0.005	0.657	−0.076
B6	−2.554	−2.396	−0.062	2.458	1.585	−11.547	−5.671	−1.047	6.719	0.049
B7	−30.351	−4.118	−0.506	4.624	0.475	−13.910	−2.444	0.931	1.514	−0.088

Table S4: DFT calculated phosphorus–electron hfccs, $A_{e\mu}$, and associated resonance fields for possible apical/apical isomers of $[MuFe_2(pdt)(CO)_4(PMe_3)_2]$ muoniated radicals. Reference structures are displayed in Figure S16.

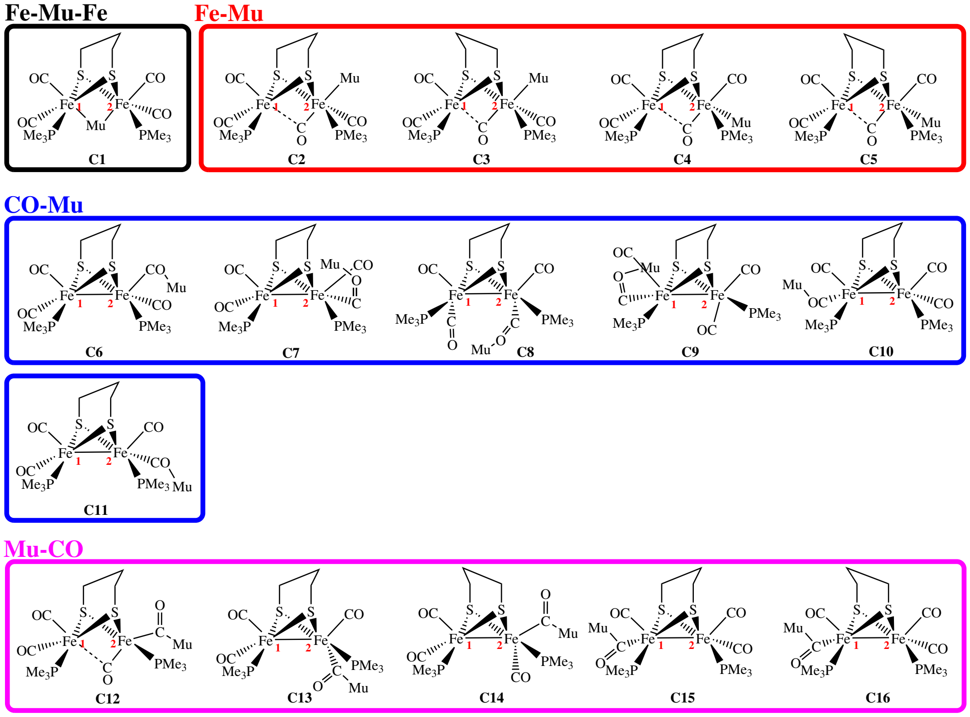

Figure S17: Possible muoniated radicals of the *cis*-basal/basal structural isomer, **C**, of the $[MuFe_2(pdt)(CO)_4(PMe_3)_2]$ model system.

Table S5: DFT calculated isotropic muon-electron hyperfine coupling constants, $A_{e\mu}$, anisotropic muon hyperfine tensors and associated resonance fields for possible *cis*-basal/basal isomers of $[MuFe_2(pdt)(CO)_4(PMe_3)_2]$ muoniated radicals. Reference structures are displayed in Figure S17.

Radical	$A_{e\mu}$/MHz	Muon dipole tensor/MHz			$\Delta_1 B_{res}$/T
		A_{aa}	A_{bb}	A_{cc}	
C1	−207.052	19.545	−2.656	22.200	0.760
C2	272.045	−6810	−5.871	12.681	0.999
C3	76.785	−3291	−2.312	5.603	0.282
C4	−81.890	−7770	−5.568	13.388	0.301
C5	−4.907	−3203	−2.332	5.535	0.018
C6	300.042	−2517	−1.922	4.439	1.102
C7	396.232	−5040	−2.358	7.398	1.455
C8	649.833	−5064	−3.587	8.651	2.386
C9	397.150	−5051	−2.389	7.440	1.458
C10	70.552	−1194	−0.823	2.018	0.259
C11	72.997	−1079	−0.720	1.799	0.268
C12	−53.451	−3037	−2.802	5.839	0.196
C13	108.614	−2085	−1.426	3.511	0.399
C14	−34.043	−2999	−2.480	5.479	0.125
C15	121.337	−2618	−1.220	3.839	0.446
C16	129.979	−2109	−1.548	3.656	0.477

Radical	$A_{P1,e\mu}$/MHz	Muon dipole tensor/MHz			$\Delta_1 B_{res}$/T	$A_{P2,e\mu}$/MHz	Muon dipole tensor/MHz			$\Delta_1 B_{res}$/T
		A_{aa}	A_{bb}	A_{cc}		A_{aa}	A_{bb}	A_{cc}		
C1	−39.032	−5.341	2.167	3.174	0.899	−40.775	−5.701	2.526	3.174	0.760
C2	−38.785	−6.124	2.75	3.374	1.668	−10.390	−1.143	0.271	0.872	0.999
C3	−59.028	−7.995	3.488	4.507	0.730	9.425	−0.800	−0.709	1.509	0.282
C4	−34.882	−5.479	2.309	3.17	0.251	−25.718	−3.842	1.259	2.583	0.301
C5	−57.718	−7.882	3.422	4.46	0.285	6.914	−0.613	−0.179	0.792	0.018
C6	−28.167	−4.061	1.343	2.718	1.761	−2.789	−0.719	−0.043	0.762	1.102
C7	−3.537	−0.274	−0.023	0.298	2.144	100.197	−8.404	−5.982	14.386	1.455
C8	2.390	−0.199	−0.129	0.328	3.472	77.838	−4.013	−2.230	6.243	2.386
C9	100.005	−8.683	−6.056	14.739	1.590	−3.660	−0.283	−0.015	0.298	1.458
C10	31.929	−1.68	−1.401	3.081	0.206	−51.473	−7.863	2.988	4.875	0.259
C11	−11.107	−1.745	0.582	1.162	0.451	−50.797	−7.017	2.883	4.134	0.268
C12	−14.988	−1.923	0.633	1.29	0.206	−55.007	−10.239	1.685	8.554	0.196
C13	−47.985	−6.4	2.373	4.026	0.841	7.714	−3.564	−1.546	5.11	0.399
C14	−62.503	−8.691	2.294	6.396	0.155	−18.136	−2.089	0.773	1.316	0.125
C15	18.042	−6.289	−1.81	8.099	0.553	−43.726	−6.147	1.999	4.147	0.446
C16	6.575	−3.564	−0.744	4.308	0.662	−44.778	−6.963	2.310	4.653	0.277

Table S6: DFT calculated phosphorus–electron hfccs, $A_{e\mu}$, and associated resonance fields for possible *cis*-basal/basal isomers of $[MuFe_2(pdt)(CO)_4(PMe_3)_2]$ muoniated radicals. Reference structures are displayed in Figure S17.

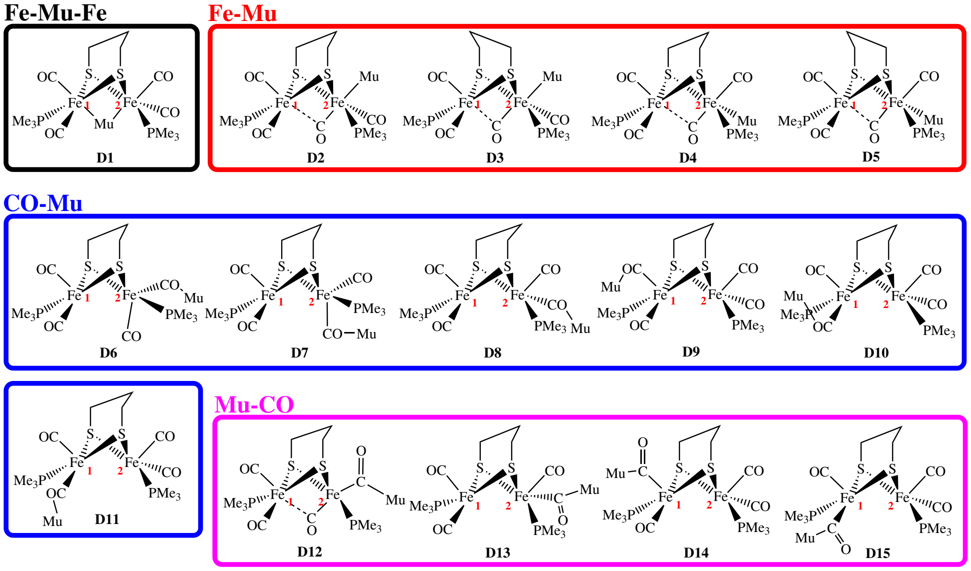

Figure S18: Possible muoniated radicals of the *trans*-basal/basal structural isomer, **D**, of the [$MuFe_2$(pdt)$(CO)_4(PMe_3)_2$] model system.

Table S7: DFT calculated isotropic muon-electron hyperfine coupling constants, $A_{e\mu}$, anisotropic muon hyperfine tensors and associated resonance fields for possible *cis*-basal/basal isomers of $[MuFe_2(pdt)(CO)_4(PMe_3)_2]$ muoniated radicals. Reference structures are displayed in Figure S17.

Radical	$A_{e\mu}$/MHz	Muon dipole tensor/MHz			$\Delta_1 B_{res}$/T
		A_{aa}	A_{bb}	A_{cc}	
D1	−203.23	−19.65	−1.97	21.62	0.75
D2	299.52	−6.50	−5.56	12.06	1.10
D3	222.17	−5.47	−4.23	9.70	0.82
D4	−86.78	−8.43	−6.19	14.62	0.32
D5	−64.12	−7.07	−5.39	12.46	0.23
D6	223.76	−2.09	−1.42	3.50	0.82
D7	232.36	−2.07	−1.72	3.79	0.85
D8	64.26	−4.92	−2.78	7.70	0.24
D9	220.6	−2.04	−1.40	3.44	0.81
D10	186.70	−1.81	−1.44	3.25	0.69
D11	59.16	−3.40	−2.24	6.24	0.22
D12	−44.35	−3.05	−2.31	5.36	0.16
D13	144.81	−2.46	−1.49	3.95	0.53
D14	7.77	−3.09	−1.72	4.80	0.03
D15	128.17	−2.64	−1.19	3.83	0.47

Radical	$A_{P1,e\mu}$/MHz	Muon dipole tensor/MHz			$\Delta_1 B_{res}$/T	$A_{P2,e\mu}$/MHz	Muon dipole tensor/MHz			$\Delta_1 B_{res}$/T
		A_{aa}	A_{bb}	A_{cc}		A_{aa}	A_{bb}	A_{cc}		
D1	39.552	−6.450	2.398	4.052	0.876	38.821	−5.933	2.250	3.684	0.88
D2	15.690	−1.880	0.760	1.120	1.691	37.932	−6.815	2.775	4.040	0.121
D3	8.669	−1.062	0.434	0.628	1.238	40.49	−7.526	2.808	4.718	0.17
D4	32.05	−5.067	1.899	3.169	0.29	38.35	−6.650	3.034	3.617	0.04
D5	25.20	−4.046	1.479	2.567	0.21	41.66	−7.217	3.291	3.925	0.09
D6	14.62	−2.411	0.902	1.509	1.28	42.84	−5.939	2.579	3.360	0.15
D7	56.60	−3.645	−3.381	7.026	0.94	40.74	−5.914	2.244	3.670	0.52
D8	36.74	−2.380	−2.303	4.683	0.15	43.82	−6.030	2.260	3.770	0.43
D9	43.35	−6.021	2.732	3.289	1.42	11.49	−1.816	0.621	1.195	0.17
D10	47.98	−6.706	2.876	3.830	1.26	51.01	−3.086	−3.010	6.095	0.53
D11	49.67	−6.612	2.771	3.841	0.59	34.79	−2.200	−2.009	4.208	0.45
D12	56.09	−9.050	2.378	6.673	0.06	20.76	−3.356	1.474	1.883	0.19
D13	2.97	−4.455	−0.868	5.323	0.76	32.13	−5.607	1.744	3.863	0.19
D14	25.43	−3.268	1.497	1.771	0.18	55.80	−6.979	2.528	4.450	0.16
D15	37.90	−5.314	2.351	2.963	0.89	23.72	−6.984	−2.230	9.214	0.33

Table S8: DFT calculated phosphorus–electron hfccs, $A_{e\mu}$, and associated resonance fields for possible *trans*-basal/basal isomers of $[MuFe_2(pdt)(CO)_4(PMe_3)_2]$ muoniated radicals. Reference structures are displayed in Figure S18.

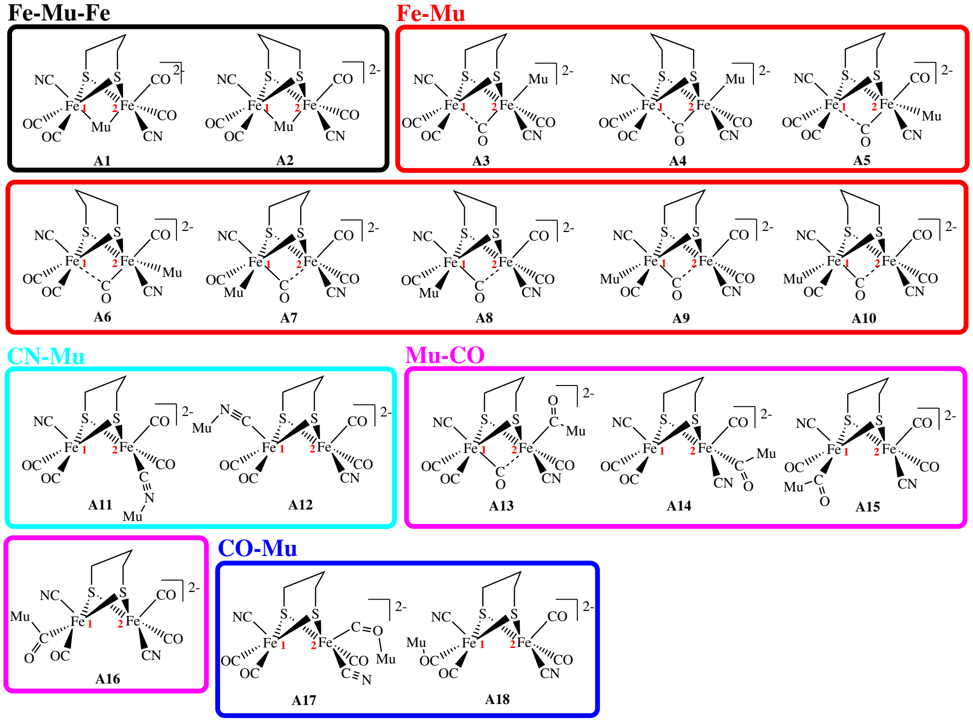

Figure S19: Possible muoniated radicals of the apical/basal structural isomer, **A**, of the $[MuFe_2(pdt)(CO)_4(CN)_2]^{2-}$ model system.

Table S9: DFT calculated isotropic muon-electron hyperfine coupling constants, $A_{e\mu}$, anisotropic muon hyperfine tensors and associated resonance fields for possible apical/basal isomers of $[MuFe_2(pdt)(CO)_4(CN)_2]$ muoniated radicals. Reference structures are displayed in Figure S19.

Radical	$A_{e\mu}$/MHz	Muon dipole tensor/MHz			$\Delta_1 B_{res}$/T
		A_{aa}	A_{bb}	A_{cc}	
A1	−213.949	−19.615	−2.449	22.065	0.786
A2	−224.598	−20.031	−2.755	22.786	0.825
A3	295.082	−5.989	−4.894	10.883	1.084
A4	73.69	−2.936	−2.000	4.936	0.271
A5	−65.138	−7.014	−4.799	11.813	0.239
A6	−4.404	−3.222	−2.421	5.643	0.016
A7	−23.921	−4.811	−3.868	8.679	0.087
A8	−81.162	−9.794	−8.265	18.059	0.298
A9	−42.956	−6.578	−5.390	11.968	0.158
A10	−76.204	−9.651	−7.906	17.557	0.280
A11	−28.734	−1.635	−1.105	2.740	0.106
A12	40.171	−0.666	−0.523	1.189	0.148
A13	465.331	−5.124	0.96	4.164	1.709
A14	92.470	−2.024	−0.926	2.949	0.340
A15	−62.397	−4.212	−3.663	7.874	0.229
A16	339.849	−7.574	−2.25	9.824	1.248
A17	277.621	−5.013	−1.455	6.468	1.019
A18	105.963	−11.729	−6.351	18.08	0.389

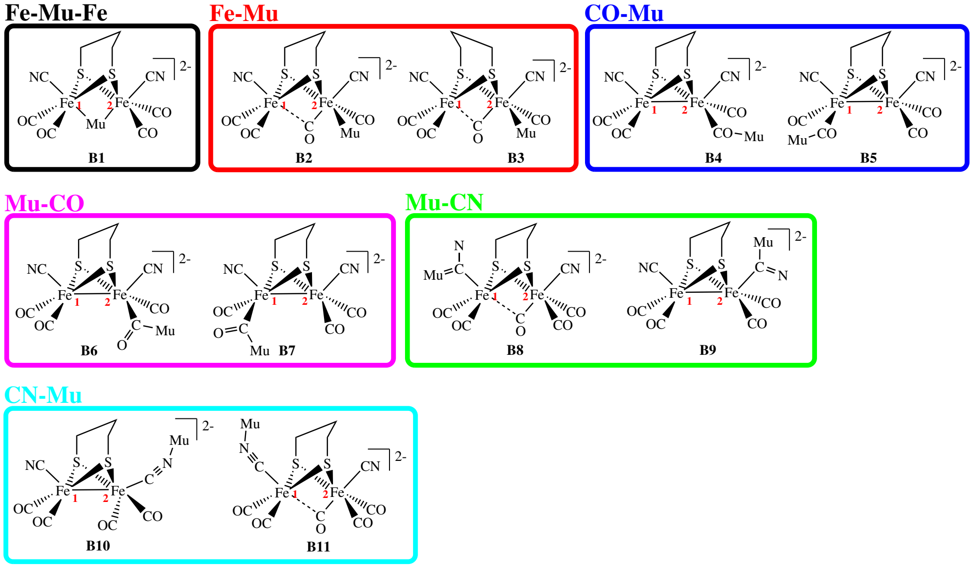

Figure S20: Possible muoniated radicals of the apical/apical structural isomer, **B**, of the $[MuFe_2(pdt)(CO)_4(CN)_2]^{2-}$ model system.

Table S10: DFT calculated isotropic muon-electron hyperfine coupling constants, $A_{e\mu}$, anisotropic muon hyperfine tensors and associated resonance fields for possible apical/apical isomers of $[MuFe_2(pdt)(CO)_4(CN)_2]$ muoniated radicals. Reference structures are displayed in Figure S20.

Radical	$A_{e\mu}$/MHz	Muon dipole tensor/MHz			$\Delta_1 B_{res}$/T
		A_{aa}	A_{bb}	A_{cc}	
B1	−242.669	−22.447	−2.833	25.28	−0.891
B2	−89.422	−10.886	−8.789	19.675	−0.328
B3	−70.876	−9.163	−7.556	16.719	−0.260
B4					
B5	388.939	−4.103	−3.407	7.510	1.428
B6	−56.234	−4.378	−4.008	8.386	−0.207
B7	−59.281	−4.142	−3.610	7.752	−0.218
B8	729.352	−3.067	−2.981	6.048	2.678
B9	765.370	−3.183	−2.894	6.077	2.811
B10	8.157	−1.201	−0.823	2.024	0.030
B11	19.848	−1.112	−0.564	1.609	0.073

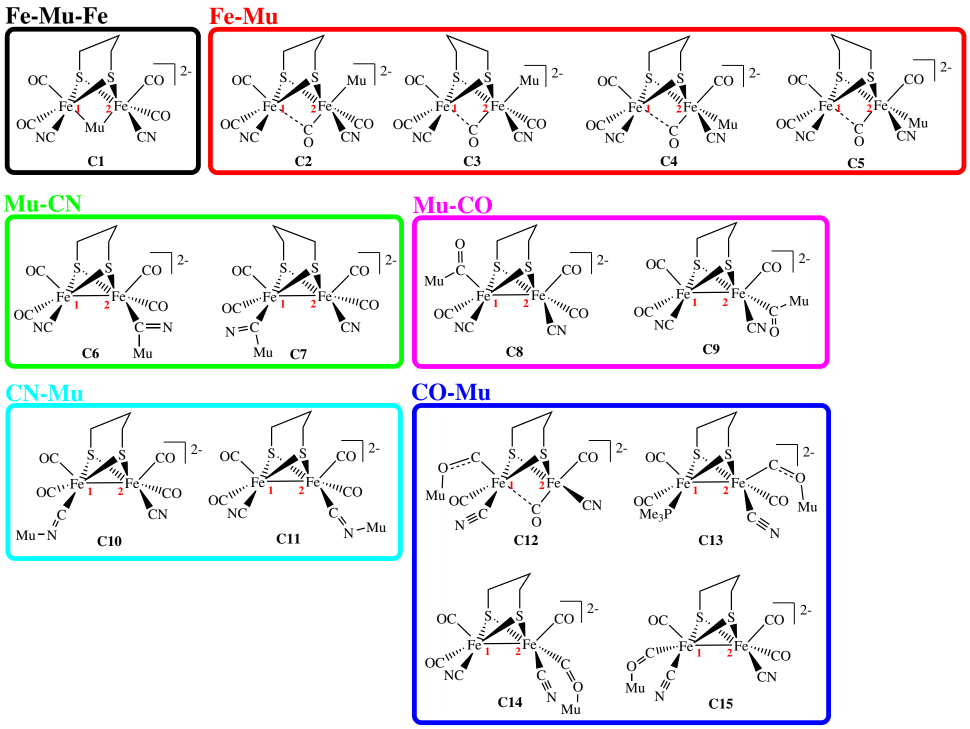

Figure S21: Possible muoniated radicals of the *cis*-basal/basal structural isomer, **C**, of the $[MuFe_2(pdt)(CO)_4(CN)_2]^{2-}$ model system.

Table S11: DFT calculated isotropic muon-electron hyperfine coupling constants, $A_{e\mu}$, anisotropic muon hyperfine tensors and associated resonance fields for possible *cis*-basal/basal isomers of $[MuFe_2(pdt)(CO)_4(CN)_2]$ muoniated radicals. Reference structures are displayed in Figure S21.

Radical	$A_{e\mu}$/MHz	Muon dipole tensor/MHz			$\Delta_1 B_{res}$/T
		A_{aa}	A_{bb}	A_{cc}	
C1	−221.762	−18.341	−2.858	21.200	−0.814
C2	65.622	−2.412	−1.879	4.291	0.241
C3	47.020	−2.358	−1.602	3.960	0.173
C4	−5.441	−2.753	−1.878	4.630	−0.020
C5	6.320	−2.548	−1.892	4.440	0.023
C6	653.323	−3.965	−0.119	4.083	2.399
C7	677.358	−3.982	−0.315	4.297	2.487
C8	370.662	−4.560	0.154	4.406	1.361
C9	83.680	−1.704	−0.921	2.625	0.307
C10	41.562	−0.635	−0.476	1.111	0.153
C11	39.345	−0.639	−0.451	1.090	0.144
C12	505.090	−3.434	−1.972	5.405	1.855
C13	258.029	−4.985	−1.164	6.150	0.948
C14	312.849	−6.342	−1.387	7.729	1.149
C15	309.912	−6.369	−1.331	7.700	1.138

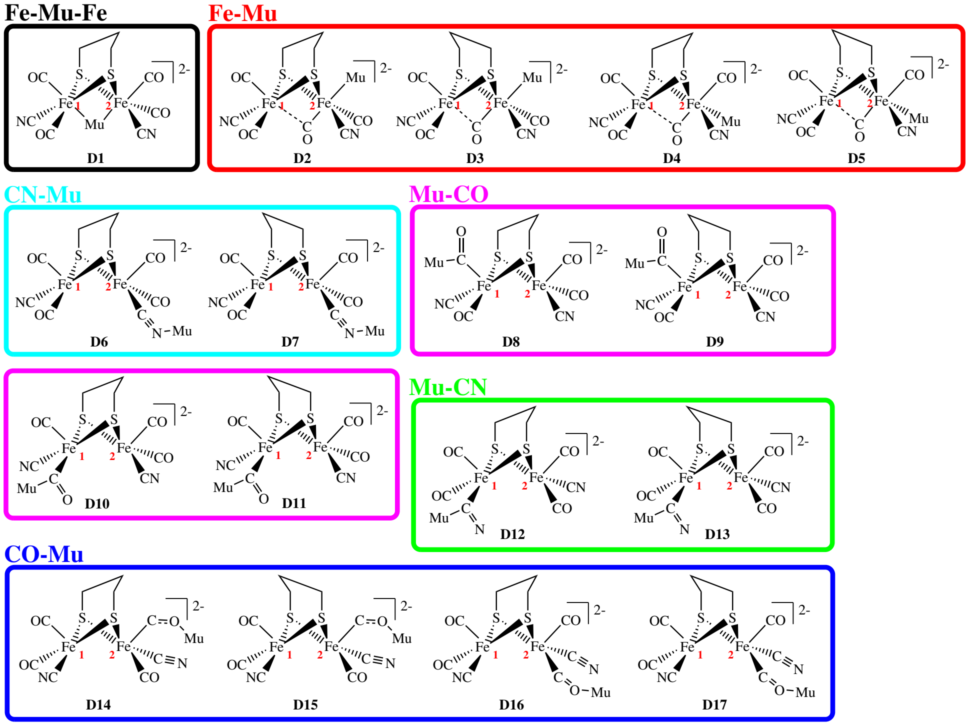

Figure S22: Possible muoniated radicals of the *trans*-basal/basal structural isomer, **D**, of the $[MuFe_2(pdt)(CO)_4(CN)_2]^{2-}$ model system.

Table S12: DFT calculated isotropic muon-electron hyperfine coupling constants, $A_{e\mu}$, anisotropic muon hyperfine tensors and associated resonance fields for possible *trans*-basal/basal isomers of $[MuFe_2(pdt)(CO)_4(CN)_2]$ muoniated radicals. Reference structures are displayed in Figure S22.

Radical	$A_{e\mu}$/MHz	Muon dipole tensor/MHz			$\Delta_1 B_{res}$/T
		A_{aa}	A_{bb}	A_{cc}	
D1	−215.652	−18.201	−2.683	20.884	−0.792
D2	87.523	−2.881	−2.244	5.125	0.321
D3	55.454	−2.549	−1.696	4.245	0.204
D4	−48.341	−5.307	−3.953	9.260	−0.178
D5	5.763	−2.581	−2.004	4.585	0.021
D6	17.127	−0.649	−0.420	1.069	0.063
D7	−12.056	−1.215	−0.897	2.112	−0.044
D8	235.987	−3.533	0.230	3.304	0.867
D9	418.314	−4.587	0.587	4.000	1.536
D10	82.591	−1.958	−0.828	2.786	0.303
D11	80.477	−1.615	−0.977	2.592	0.296
D12	733.576	−3.949	−1.289	5.238	2.694
D13	718.648	−3.906	−1.188	5.094	2.639
D14	290.729	−4.778	−1.655	6.434	1.068
D15	276.445	−4.636	−1.493	6.129	1.015
D16	321.594	−6.128	−1.574	7.703	1.181
D17	343.070	−6.090	−1.779	7.869	1.260

References

(S1) Gloaguen, F.; Lawrence, J. D.; Rauchfuss, T. B. *J. Am. Chem. Soc.* **2001**, *123*, 9476–9477.

(S2) Seyferth, D.; Womack, G. B.; Gallagher, M. K.; Cowie, M.; Hames, B. W.; Fackler, J. P.; Mazany, A. M. *Organometallics* **1987**, *6*, 283–294.

(S3) Jablonskyt, A.; Wright, J. A.; Pickett, C. J. *Dalton Trans.* **2010**, *39*, 3026–3034.

(S4) Arnold, O.; Bilheux, J. C.; Borreguero, J. M.; Buts, A.; Campbell, S. I.; Chapon, L.; Doucet, M.; Draper, N.; Ferraz Leal, R.; Gigg, M. A.; Lynch, V. E.; Markvardsen, A.; Mikkelson, D. J.; Mikkelson, R. L.; Miller, R.; Palmen, K.; Parker, P.; Passos, G.; Perring, T. G.; Peterson, P. F.; Ren, S.; Reuter, M. A.; Savici, A. T.; Taylor, J. W.; Taylor, R. J.; Tolchenov, R.; Zhou, W.; Zikovsky, J. *Nucl. Instrum. Methods Phys. Res., Sect. A* **2014**, *764*, 156–166.

(S5) Frisch, M. J.; Trucks, G. W.; Schlegel, H. B.; Scuseria, G. E.; Robb, M. A.; Cheeseman, J. R.; Scalmani, G.; Barone, V.; Mennucci, B.; Petersson, G. A.; Nakatsuji, H.; Caricato, M.; Li, X.; Hratchian, H. P.; Izmaylov, A. F.; Bloino, J.; Zheng, G.; Sonnenberg, J. L.; Hada, M.; Ehara, M.; Toyota, K.; Fukuda, R.; Hasegawa, J.; Ishida, M.; Nakajima, T.; Honda, Y.; Kitao, O.; Nakai, H.; Vreven, T.; Montgomery, J. A., Jr.; Peralta, J. E.; Ogliaro, F.; Bearpark, M.; Heyd, J. J.; Brothers, E.; Kudin, K. N.; Staroverov, V. N.; Kobayashi, R.; Normand, J.; Raghavachari, K.; Rendell, A.; Burant, J. C.; Iyengar, S. S.; Tomasi, J.; Cossi, M.; Rega, N.; Millam, J. M.; Klene, M.; Knox, J. E.; Cross, J. B.; Bakken, V.; Adamo, C.; Jaramillo, J.; Gomperts, R.; Stratmann, R. E.; Yazyev, O.; Austin, A. J.; Cammi, R.; Pomelli, C.; Ochterski, J. W.; Martin, R. L.; Morokuma, K.; Zakrzewski, V. G.; Voth, G. A.; Salvador, P.; Dannenberg, J. J.; Dapprich, S.; Daniels, A. D.; Farkas, Ö.; Foresman, J. B.; Ortiz, J. V.;

Cioslowski, J.; Fox, D. J. Gaussian 09 Revision C.01. Gaussian, Inc.: Wallingford, CT, 2009.

(S6) Tao, J.; Perdew, J. P.; Staroverov, V. N.; Scuseria, E., G. *Phys. Rev. Lett.* **2003**, *91*, 146401–146405.

(S7) Hay, P. J.; Wadt, W. R. *J. Chem. Phys.* **1985**, *82*, 270–283.

(S8) Hay, P. J.; Wadt, W. R. *J. Chem. Phys.* **1985**, *82*, 284–298.

(S9) Couty, M.; Hall, M. B. *J. Comput. Chem.* **1996**, *17*, 1359–1370.

(S10) Check, C. E.; Faust, T. O.; Bailey, J. M.; Wright, B. J.; Gilbert, T. M.; Sunderlin, L. S. *J. Phys. Chem. A* **2001**, *105*, 8111–8116.

Atomic coordinates for calculated structures

Complex PMe3-A1

Fe	1.09392200	-0.60708700	0.13176800
Fe	-1.45540400	0.36546300	0.42074100
S	0.46429900	1.27583400	1.40591600
S	-0.17331400	0.40133900	-1.58094300
P	3.30314000	-0.04646900	-0.21480800
P	-2.92398600	-0.93651600	-0.61820700
O	1.71335200	-2.11550300	2.55357400
O	-2.38711100	-0.46958900	3.05160700
O	1.24575000	-3.02757200	-1.48829000
O	-3.28478400	2.67149500	0.40132200
C	1.49337900	-1.49292700	1.58448600
C	-2.01751500	-0.13609100	1.98998800
C	1.18409300	-2.04927200	-0.84042300
C	-2.49622300	1.80178200	0.31994300
C	0.99813500	2.79826200	0.48733500
C	0.36606300	2.13685400	-1.93638700
C	0.23344200	3.14810600	-0.79285500
C	4.35701400	-1.56061500	-0.39611200
C	-4.42332600	-1.39613800	0.35898300

C	3.90802800	0.95100700	-1.66268300
C	-3.66204700	-0.22003900	-2.15370700
C	4.14819100	0.78535000	1.21229600
C	-2.30242500	-2.57298300	-1.20143200
H	-1.49429400	-2.42150900	-1.92507800
H	4.06866300	-2.10781900	-1.30096900
H	-4.93592800	-0.48678400	0.69432100
H	4.20462600	-2.22075600	0.46592900
H	-4.12962600	-1.97191600	1.24354300
H	3.59252900	1.99608500	-1.56533700
H	-4.36663200	-0.92657000	-2.61204700
H	3.48398100	0.54245700	-2.58781100
H	-2.86012000	0.00824700	-2.86422500
H	5.00447900	0.92111500	-1.73052900
H	-4.19089500	0.70826600	-1.90889600
H	5.42041800	-1.29393900	-0.45890200
H	-5.11400000	-1.99470300	-0.24899100
H	5.22175200	0.91072700	1.01712400
H	-3.10812600	-3.14896900	-1.67571300
H	4.01470900	0.16821300	2.10852200
H	-1.90298400	-3.13615000	-0.35064800
H	3.70180100	1.76550200	1.41209800
H	0.62474100	4.11270700	-1.15957300
H	-0.82481000	3.31136600	-0.55996600
H	0.89839000	3.61606200	1.21134100
H	2.06656100	2.67208900	0.26527400
H	-0.24522000	2.45891900	-2.78873600
H	1.40596300	2.06969500	-2.27797300
H	-0.49902100	-0.99051500	0.56539100

Complex PMe3-A2

Fe	1.08261100	-0.58799400	0.20941700
Fe	-1.47749100	0.36539800	0.41761600
S	0.39907200	1.29866400	1.44744300
S	-0.15763800	0.36833800	-1.56013800
P	3.28558500	-0.10517600	-0.25789500
P	-2.93818400	-0.92085500	-0.65142900
O	1.68253400	-1.96327400	2.71531400
O	-2.56980700	-0.33605600	3.02542600
O	1.21681600	-3.11044900	-1.25359000
O	-3.07769700	2.82292200	0.16331800
C	1.46728400	-1.39955000	1.71048400
C	-2.13284000	-0.05535100	1.97418900
C	1.15894000	-2.09077400	-0.67206200
C	-2.40146500	1.86063900	0.19183300
C	0.71829200	2.89827800	0.57089200
C	0.22458800	2.15389800	-1.87131400
C	1.19120900	2.81132800	-0.88318400
C	4.37394500	-1.56913500	0.07455900
C	-4.45661800	-1.38619300	0.29334100
C	3.79111500	0.28890800	-2.00131800
C	-3.64853400	-0.19504700	-2.19567400
C	4.15801800	1.18829900	0.74733300
C	-2.31186700	-2.55657900	-1.23235800
H	-1.48786900	-2.40500800	-1.93797400
H	4.05825700	-2.41568900	-0.54648300
H	-4.97766300	-0.47950300	0.62256900
H	4.28664300	-1.86314600	1.12706700
H	-4.17945800	-1.96495500	1.18130600
H	3.33777200	1.22737300	-2.33870900
H	-4.33994000	-0.90076400	-2.67491100
H	3.43547400	-0.51469100	-2.65741800
H	-2.83348500	0.04316900	-2.88784800
H	4.88300100	0.36850300	-2.09069000
H	-4.18769700	0.72824800	-1.95412000
H	5.42443400	-1.33673600	-0.14531000
H	-5.13396600	-1.98372600	-0.33046500
H	5.23300900	1.20725700	0.52241200
H	-3.10925200	-3.12654900	-1.72760600
H	4.01713200	0.95789800	1.81010500
H	-1.93330700	-3.12601300	-0.37629900
H	3.73751900	2.18151100	0.55657500
H	-0.21321500	3.47520400	0.64166400
H	1.47732900	3.41280500	1.17405300

H	-0.73900600	2.68064700	-1.88135800
H	0.64719600	2.19411400	-2.88320500
H	2.15072500	2.28194600	-0.91948300
H	1.38258100	3.83970100	-1.23444800
H	-0.55047200	-0.97314800	0.64660900

Complex PMe3-A3

Fe	1.10704000	-0.54534800	0.17668200
Fe	-1.48497100	0.26165000	0.50199500
S	0.34232500	1.05222500	1.70686100
S	-0.12869800	0.66367800	-1.40823500
O	1.32879100	-2.82614300	-1.64051800
O	-1.18829500	-2.62980700	0.89234800
C	1.22385500	-1.90760900	-0.91674900
C	-2.46238300	0.28302800	1.93180200
C	-1.03382400	-1.47758600	0.67818100
C	0.67660900	2.79462900	1.16597100
C	0.41613900	2.44077200	-1.38118100
C	0.03337500	3.25644100	-0.14465700
H	1.50653500	2.43088600	-1.51329300
H	-0.02617000	2.89081500	-2.27852600
H	0.34664500	4.29994500	-0.32083100
H	-1.05882000	3.25655000	-0.03278600
H	0.31510000	3.41635800	1.99430800
H	1.76782700	2.89461000	1.12388000
C	1.66525100	-1.49729300	1.53858800
O	2.05997800	-2.13981700	2.43531000
O	-3.13485300	0.36464300	2.88686100
P	-3.13948900	-0.23456200	-0.88087100
P	3.24438600	0.10485600	-0.41584900
C	-4.70766400	-0.80607500	-0.09018300
H	-5.08133300	-0.03446400	0.59237800
H	-5.47185500	-1.01931400	-0.84890200
H	-4.51321500	-1.71515600	0.49080200
C	-2.81125500	-1.56370400	-2.12241800
H	-3.67869600	-1.69689200	-2.78232300
H	-1.93664100	-1.29034400	-2.72368600
H	-2.60091900	-2.50793000	-1.60780300
C	-3.70675900	1.16997100	-1.93521700
H	-2.85630700	1.54627600	-2.51453600
H	-4.50416800	0.85375600	-2.62059300
H	-4.07482500	1.97410400	-1.28839800
C	4.09393600	1.58566800	0.31631300
H	3.99271800	1.56026500	1.40785800
H	5.16179700	1.59389300	0.05727200
H	3.63683500	2.50982000	-0.05532200
C	3.54619800	0.34170900	-2.23148600
H	4.60732600	0.54130600	-2.43358500
H	3.24468700	-0.56927200	-2.76188700
H	2.94389700	1.17131200	-2.61837100

C	4.48624100	-1.21678000	-0.03460700
H	4.17822200	-2.15746100	-0.50597300
H	5.48161700	-0.93823600	-0.40588800
H	4.53798100	-1.37757800	1.04838200
H	-2.12678300	1.66399200	0.38688100

Complex PMe3-A4

Fe	1.29044900	-0.56506500	0.09067800
Fe	-1.62851400	0.18116400	0.52624900
S	0.32285200	0.88326000	1.70151400
S	-0.20523600	0.53908600	-1.39342500
O	1.41317300	-2.75870300	-1.83919000
O	-1.44563500	-2.73344500	0.91099900
C	1.37001400	-1.89792900	-1.07576100
C	-2.58126900	0.40416200	1.98218000
C	-1.38658600	-1.60063500	0.72736200
C	0.47962100	2.65363100	1.19510100
C	0.04752000	2.37182700	-1.35920100
C	0.92668100	2.91982200	-0.23899100
H	0.49425600	2.62695000	-2.32328900
H	-0.94727200	2.81790900	-1.31594100
H	1.94355800	2.54684800	-0.37081700
H	0.98379500	4.00971200	-0.37043000
H	-0.49214700	3.11027400	1.38957400
H	1.20427400	3.09321400	1.88495300
C	1.80795800	-1.60196400	1.43546100
O	2.14893300	-2.26575900	2.31058100
O	-3.19494800	0.63364100	2.92422500
P	-3.36779800	-0.12994900	-0.86221100
P	3.49288800	0.18359800	-0.36970900
C	-5.00162800	-0.44977900	-0.07188000
H	-5.26543000	0.38675500	0.57762500
H	-5.78344700	-0.57898100	-0.82428400
H	-4.94536800	-1.35103100	0.54188400
C	-3.21428700	-1.52482700	-2.05371100
H	-4.08327600	-1.57480900	-2.71482600
H	-2.31446800	-1.38138300	-2.65412800
H	-3.12408200	-2.46896300	-1.51389400
C	-3.73905900	1.28762400	-1.97332500
H	-2.85776900	1.50715300	-2.57714700
H	-4.57894700	1.05726400	-2.63398000
H	-3.97961800	2.16600100	-1.37278500
C	4.31827600	1.29813400	0.85398600
H	4.29515300	0.82250000	1.83666200
H	5.35733800	1.49908200	0.57875000
H	3.78190300	2.24521300	0.92888900
C	3.83414100	1.02787300	-1.98089000
H	4.90162200	1.22706200	-2.11020000
H	3.48976500	0.38744700	-2.79558000
H	3.28935600	1.97094400	-2.04311400

C	4.71525300	-1.20091000	-0.45056000
H	4.42282300	-1.90299800	-1.23389800
H	5.72328600	-0.83335600	-0.66061500
H	4.72495300	-1.73844800	0.49970000
H	-2.04078500	1.64674000	0.39462400

Complex PMe3-A5

Fe	-1.06372300	-0.51044300	-0.29201600
Fe	1.48040100	0.30747700	-0.54641900
S	-0.34469800	1.29067600	-1.61466600
S	0.18645800	0.49047500	1.45112400
O	-1.18734400	-3.06699100	1.12562400
O	1.04500400	-2.35518200	-1.66732900
C	-1.12743200	-2.03382600	0.57170000
C	2.55480700	1.69993900	-0.74920700
C	0.91752500	-1.30278800	-1.13616100
C	-0.67862900	2.93397000	-0.82574900
C	-0.40089300	2.24182200	1.65275300
C	-0.02535500	3.21360200	0.53264800
H	-1.49143100	2.20308900	1.77774900
H	0.03317700	2.57930100	2.60205400
H	-0.33894000	4.22437700	0.84612400
H	1.06567600	3.24282600	0.42544600
H	-0.32632900	3.66924500	-1.56002000
H	-1.76903900	3.02885700	-0.75778500
C	-1.74695600	-1.20844200	-1.74768600
O	-2.23052000	-1.67945200	-2.70414300
O	3.35288000	2.50089900	-1.07262300
P	3.02222700	-0.76165500	0.60039200
P	-3.15993700	0.03812900	0.51877200
C	4.38217900	-1.49430500	-0.40980500
H	4.89589700	-0.69752400	-0.96004300
H	5.10579700	-2.02661900	0.22131300
H	3.94607700	-2.19131200	-1.13454100
C	2.43401500	-2.18919500	1.61506900
H	3.26915500	-2.63118800	2.17454700
H	1.67322500	-1.83830400	2.32221400
H	1.98775800	-2.95563400	0.97206300
C	3.90868500	0.25270600	1.86395800
H	3.17036700	0.65073200	2.56931400
H	4.64238400	-0.35598200	2.40841800
H	4.42285800	1.09181500	1.38186800
C	-4.03164400	1.62487600	0.09730800
H	-4.00128200	1.77966500	-0.98778100
H	-5.08065400	1.59122400	0.42327200
H	-3.54125700	2.47469000	0.58505100
C	-3.35492700	-0.04134400	2.36162300
H	-4.39376900	0.16144300	2.65456500
H	-3.07387200	-1.04388300	2.70436600
H	-2.69206600	0.67928500	2.85240600

C	-4.44123300	-1.19141900	-0.01194600
H	-4.12442700	-2.20125100	0.27389500
H	-5.40882000	-0.97235100	0.45876500
H	-4.55720500	-1.16453400	-1.10141600
H	2.16955500	0.10586400	-1.86259500

Complex PMe3-A6

Fe	-1.11888400	-0.50298700	-0.18547300
Fe	1.51867100	0.26106900	-0.60758500
S	-0.38346700	1.16049800	-1.65290900
S	0.25515900	0.51817100	1.41665200
O	-0.97903500	-2.99120500	1.34599200
O	1.20149300	-2.38660200	-1.81531800
C	-1.03686100	-1.98187900	0.74622400
C	2.42365100	1.76975500	-0.75516500
C	1.15602600	-1.35387700	-1.25476400
C	-0.60027500	2.86308400	-0.95534100
C	0.01020700	2.35685200	1.51843300
C	-0.98757800	2.95375200	0.52306500
H	-0.33361900	2.54711800	2.54319100
H	1.00223300	2.81213000	1.39946700
H	0.33560100	3.40378400	-1.14712700
H	-1.38374400	3.32405000	-1.57036300
C	-1.73895400	-1.37583400	-1.57167600
O	-2.18819100	-1.96965500	-2.47830300
O	3.10958300	2.68309500	-1.03155000
P	3.11136500	-0.65123000	0.60579000
P	-3.23557000	0.01203700	0.50098200
C	4.56135700	-1.29553500	-0.33946200
H	5.01965400	-0.47709100	-0.90655900
H	5.30924100	-1.73637200	0.33230500
H	4.21721600	-2.05658100	-1.04914300
C	2.59359300	-2.09693200	1.62946500
H	3.44299300	-2.48201500	2.20922300
H	1.79955900	-1.78343800	2.31650700
H	2.20381000	-2.89545500	0.98867600
C	3.88184000	0.45629800	1.86645400
H	3.09801100	0.82254800	2.53927300
H	4.63617000	-0.08644400	2.45112200
H	4.35734600	1.31379300	1.37686600
C	-4.23667700	1.19191900	-0.52449800
H	-4.23817500	0.83525600	-1.56147700
H	-5.27287600	1.26005500	-0.16594800
H	-3.78762300	2.19121200	-0.51066400
C	-3.50105300	0.62153100	2.23387600
H	-4.57137100	0.69334600	2.47047100
H	-3.02389600	-0.07934900	2.92896500
H	-3.03942800	1.60546300	2.37227000
C	-4.34228000	-1.47502900	0.48853800
H	-3.93542000	-2.24145300	1.15870400

H	-5.35814500	-1.21510000	0.81513300
H	-4.38902600	-1.89313600	-0.52377300
H	-1.11612600	4.02104900	0.77206300
H	-1.96354500	2.47399500	0.66929300
H	2.26393200	0.17199400	-1.90787000

Complex PMe3-A7

Fe	-1.17552100	-0.65102300	-0.16736600
Fe	1.33697300	0.21862000	-0.44459600
S	-0.46484600	0.83171600	-1.81009200
S	-0.07506800	0.79810100	1.37413700
O	0.82397300	-2.75546900	-0.22400800
C	2.32357300	1.69206600	-0.64471700
C	0.33896200	-1.66325100	-0.25114300
C	-0.98184100	2.57282000	-1.43663100
C	-0.73276500	2.51958300	1.14505000
C	-0.41554000	3.22960400	-0.17367600
H	-1.82075500	2.45897100	1.28603600
H	-0.32206200	3.09710400	1.98254300
H	-0.84426100	4.24465600	-0.11016200
H	0.66774400	3.35895100	-0.27455500
H	-0.68380900	3.15635500	-2.31636400
H	-2.07862900	2.55239300	-1.40324100
C	-1.85947200	-1.71010200	-1.35088800
O	-2.31443000	-2.43302800	-2.15573700
O	3.11329400	2.52770400	-0.88488000
P	2.75721300	-0.61741300	1.09758700
P	-3.26239700	-0.18028800	0.62955500
C	4.07477800	-1.72670900	0.43679200
H	4.68353300	-1.19064700	-0.30017200
H	4.72165000	-2.08765100	1.24693100
H	3.59783100	-2.58155800	-0.05603600
C	2.04778300	-1.61760900	2.48108000
H	2.83304800	-1.83052100	3.21841700
H	1.23990600	-1.05453500	2.96130500
H	1.64545300	-2.55795100	2.09317800
C	3.69977100	0.67704200	2.01832700
H	2.98670300	1.35548700	2.50098100
H	4.33848200	0.21803000	2.78404900
H	4.32287700	1.25559700	1.32794800
C	-4.33392300	1.17922500	-0.05805900
H	-4.40909100	1.06068400	-1.14574900
H	-5.34349400	1.14826700	0.37495800
H	-3.89105700	2.15948800	0.15257800
C	-3.34927400	0.12960000	2.45275500
H	-4.39102200	0.26081200	2.77345200
H	-2.91015900	-0.73024900	2.97064300
H	-2.77026200	1.01814900	2.72438200
C	-4.40747000	-1.62486600	0.46809200
H	-3.93638800	-2.50706500	0.91575400

H	-5.36075700	-1.42190100	0.97324100
H	-4.59944800	-1.83808500	-0.58934600
C	2.13213000	-0.54542900	-1.80310600
O	2.69617100	-1.02287200	-2.70947800
H	-1.42972300	-1.61635800	0.97794500

Complex PMe3-A8

Fe	-1.15362100	-0.63952600	-0.10581200
Fe	1.33851600	0.19617700	-0.45564000
S	-0.49488000	0.77530900	-1.81912900
S	-0.01686700	0.83201500	1.38542500
O	0.85974900	-2.73903700	-0.09465500
C	2.19142200	1.74633500	-0.68414500
C	0.39569400	-1.63776900	-0.16494400
C	-0.84673700	2.56203600	-1.47990800
C	-0.40651000	2.61836300	1.07869000
C	-1.36551100	2.90389400	-0.08081500
H	-0.84366300	2.98237800	2.01732900
H	0.55003200	3.13613500	0.92799100
H	0.07435000	3.11725800	-1.70082000
H	-1.59523900	2.84735400	-2.23020800
C	-1.82016700	-1.79475400	-1.20553000
O	-2.27626600	-2.58715300	-1.94283200
O	2.86303000	2.67842900	-0.92458300
P	2.84740700	-0.54935900	1.04558200
P	-3.27670900	-0.20414400	0.61515100
C	4.13111400	-1.69006400	0.37144900
H	4.70242300	-1.19098600	-0.41940900
H	4.81745900	-2.01462800	1.16430400
H	3.62937000	-2.56563000	-0.05630200
C	2.22299400	-1.47949300	2.51587500
H	3.05689000	-1.68295200	3.20057000
H	1.46460800	-0.88055200	3.03184500
H	1.77470300	-2.42490000	2.19603600
C	3.83727100	0.79069500	1.84409000
H	3.15292000	1.49611300	2.32980400
H	4.51789900	0.37169400	2.59636200
H	4.42108900	1.33144800	1.09133900
C	-4.49046400	0.61741400	-0.52737400
H	-4.54445500	0.03174700	-1.45304300
H	-5.49202000	0.67305000	-0.07959700
H	-4.15664000	1.62873800	-0.78514100
C	-3.47870100	0.72607800	2.20461600
H	-4.52098300	0.68452800	2.54787500
H	-2.82547400	0.27399600	2.95957300
H	-3.18615400	1.77442000	2.08024700
C	-4.18325700	-1.76735700	1.01198800
H	-3.64392100	-2.30129500	1.80228900
H	-5.20711400	-1.55198100	1.34510700
H	-4.22009000	-2.41223200	0.12689600

C	2.13826300	-0.54763800	-1.82253500
O	2.70122200	-1.01684500	-2.73402700
H	-1.59974600	3.98208900	-0.06470000
H	-2.30980500	2.37158400	0.09390100
H	-1.33058800	-1.53454000	1.10380300

Complex PMe3-A9

Fe	-1.11128900	-0.64583500	-0.22125800
Fe	1.30244300	0.49138200	-0.35026400
S	-0.62934300	1.41337600	-1.28489800
S	0.00798700	0.23222100	1.63725200
O	0.83095000	-2.17148000	-1.74967000
C	2.22021300	1.93614700	0.14471900
C	0.35006100	-1.29659700	-1.09428400
C	-1.26671500	2.79706400	-0.22428000
C	-0.70198100	1.87220900	2.13325500
C	-0.59355700	3.02619300	1.13200300
H	-1.75205000	1.67365100	2.38072500
H	-0.18390000	2.14252200	3.06164400
H	-1.07770400	3.90430500	1.59343000
H	0.45723600	3.29827800	0.98452000
H	-1.16404500	3.69901300	-0.84016100
H	-2.34062900	2.61124600	-0.08246800
O	2.97120400	2.81952200	0.33282800
P	2.81178000	-0.92502800	0.53398000
P	-3.36060200	-0.26029500	-0.10983000
C	4.04939300	-1.60107000	-0.65457000
H	4.62807300	-0.78292500	-1.09811800
H	4.73273000	-2.29332200	-0.14616100
H	3.51912200	-2.13078600	-1.45398300
C	2.15666700	-2.44741200	1.34830700
H	2.98145600	-3.00093900	1.81647000
H	1.42742400	-2.16812800	2.11720500
H	1.66700300	-3.08877400	0.60936000
C	3.86217000	-0.19933900	1.86860200
H	3.21582300	0.14788600	2.68294700
H	4.56352400	-0.94956100	2.25650300
H	4.42692300	0.65542900	1.48059000
C	-4.06264500	0.65235200	-1.55896800
H	-3.77987000	0.11862000	-2.47303100
H	-5.15695200	0.70564800	-1.48641200
H	-3.65353300	1.66613700	-1.62119500
C	-4.19811400	0.57391900	1.33071900
H	-5.29160500	0.52856600	1.23112900
H	-3.90502800	0.06951100	2.25944500
H	-3.89639700	1.62530900	1.39766900
C	-4.33488500	-1.83247000	-0.18850800
H	-4.15067600	-2.43709900	0.70664700
H	-5.40871500	-1.61632800	-0.26247000
H	-4.01752000	-2.40998700	-1.06393000

C	2.05896600	0.51241800	-1.92361000
O	2.59479000	0.57095100	-2.96173800
C	-1.30756600	-2.18644800	0.53539700
O	-1.43519400	-3.24418800	1.03230200
H	-1.72273200	-1.14242300	-1.51317700

Complex PMe3-A10

Fe	-1.09981600	-0.59788300	-0.24402300
Fe	1.32342500	0.48045400	-0.35196200
S	-0.57421700	1.44902000	-1.31119700
S	0.01117000	0.23058200	1.63856200
O	0.84854100	-2.14127300	-1.75593100
C	2.13885300	1.96097200	0.21081900
C	0.39529900	-1.24607600	-1.10496300
C	-0.97638700	2.92263500	-0.26227300
C	-0.49753200	1.95401300	2.09784300
C	-1.49110900	2.63028900	1.15036800
H	-0.94331500	1.85994400	3.09588200
H	0.42003100	2.54920400	2.19137400
H	-0.07272400	3.54533700	-0.22587100
H	-1.73917200	3.47545600	-0.82597400
O	2.78946400	2.90444000	0.46505800
P	2.83472800	-0.91724000	0.55058200
P	-3.36055400	-0.30424200	-0.10956900
C	4.08402100	-1.59279000	-0.62610200
H	4.66680800	-0.77537800	-1.06546200
H	4.76280500	-2.28559800	-0.11237600
H	3.56027000	-2.12260400	-1.42993200
C	2.17688600	-2.44187200	1.35780400
H	2.99872900	-2.99928200	1.82648800
H	1.44470400	-2.16585100	2.12502800
H	1.68930700	-3.07863600	0.61322000
C	3.87152700	-0.18270400	1.89090500
H	3.21864500	0.16169300	2.70123900
H	4.57726800	-0.92568000	2.28492700
H	4.43175200	0.67568800	1.50358000
C	-4.10285000	1.05941600	-1.11837200
H	-3.70380100	0.99453600	-2.13692600
H	-5.19680200	0.96954100	-1.14705900
H	-3.83613800	2.03826500	-0.70538000
C	-4.20028500	-0.13314900	1.54074000
H	-5.29356900	-0.11561000	1.43434600
H	-3.91622400	-0.98803100	2.16623000
H	-3.87779100	0.78228100	2.04882300
C	-4.25945000	-1.76851800	-0.79724500
H	-3.97912000	-2.67346700	-0.24658400
H	-5.34607700	-1.62649400	-0.73037900
H	-3.97071200	-1.90259400	-1.84567500
C	2.13114700	0.54884900	-1.89717900
O	2.69771800	0.63535200	-2.91675300

H	-1.78827500	3.59050300	1.60562200
H	-2.39697000	2.01355900	1.08647800
C	-1.29800600	-2.16541400	0.45464900
O	-1.43679000	-3.24071200	0.91126500
H	-1.65148600	-1.03136600	-1.58011000

Complex PMe3-A11

Fe	1.10227000	-0.49794800	0.13320400
Fe	-1.41538000	0.39541300	0.38632700
S	0.45542400	1.35892100	1.36623300
S	-0.10966100	0.45663300	-1.61244700
P	3.26959300	-0.08793900	-0.24631500
P	-2.87184500	-0.80666300	-0.77823000
O	1.66979100	-1.95476000	2.60424600
O	-1.76275000	-1.41166400	2.62755800
O	1.09733600	-3.04982300	-1.28371100
O	-3.46381700	2.02134000	1.62654600
C	1.42695500	-1.35330300	1.62460300
C	-1.55636700	-0.70210300	1.70637200
C	1.07510100	-2.01502700	-0.72110800
C	-2.56181200	1.57267900	0.77669600
C	0.85288800	2.93848100	0.46849400
C	0.31585900	2.23517300	-1.94102400
C	0.03955400	3.21303700	-0.79783500
C	4.29736600	-1.62989400	-0.28376800
C	-4.37984100	-1.34061400	0.14730600
C	3.84159800	0.73994200	-1.80783100
C	-3.58503100	0.07060700	-2.23720000
C	4.16909900	0.89016500	1.04905600
C	-2.25652800	-2.38443700	-1.50553000
H	-1.45139300	-2.16551600	-2.21465000
H	3.96211900	-2.27873800	-1.10121800
H	-4.92195100	-0.45823700	0.50764300
H	4.17586000	-2.17664500	0.65895000
H	-4.09211100	-1.95342100	1.00901300
H	3.52401400	1.78915800	-1.81568600
H	-4.30687500	-0.56803200	-2.76344300
H	3.39624400	0.23608300	-2.67368800
H	-2.76764000	0.33981000	-2.91460400
H	4.93669200	0.70374600	-1.89449800
H	-4.08024800	0.98865300	-1.90143800
H	5.36006600	-1.39076900	-0.42525000
H	-5.04448200	-1.92375800	-0.50293400
H	5.24433800	0.94441900	0.82967800
H	-3.06626100	-2.91275500	-2.02672200
H	4.02417400	0.40897500	2.02338100
H	-1.85312800	-3.02443100	-0.71361800
H	3.76522600	1.90689200	1.11099100
H	0.28829500	4.22871100	-1.15024300
H	-1.03184900	3.20153000	-0.55584200

H	0.67933000	3.72980400	1.20823800
H	1.92772700	2.91480000	0.24071500
H	-0.28177100	2.50466700	-2.82085400
H	1.37330200	2.26038500	-2.23387500
H	-3.51476200	1.48173200	2.45322500

Complex PMe3-A12

Fe	1.09586700	-0.48790200	0.19600700
Fe	-1.45095400	0.42525500	0.36054900
S	0.39611200	1.39295700	1.36253500
S	-0.09633500	0.37508800	-1.61105600
P	3.26834300	-0.15866600	-0.25212100
P	-2.89124200	-0.82383400	-0.77836300
O	1.55673600	-1.80030400	2.76765500
O	-1.98315100	-1.15084900	2.73986900
O	1.05937800	-3.13267800	-1.03780900
O	-3.42211400	2.34371600	1.27127700
C	1.36711800	-1.25840800	1.74314500
C	-1.71727300	-0.53862400	1.76616400
C	1.04982600	-2.05794100	-0.55474200
C	-2.51317700	1.71405600	0.55316500
C	0.70027000	2.98412000	0.46334200
C	0.19852100	2.18145300	-1.93448200
C	1.18044000	2.86615300	-0.98371300
C	4.31430200	-1.63853500	0.13612400
C	-4.42333300	-1.31447900	0.13282100
C	3.77465300	0.16608300	-2.00890500
C	-3.56974000	-0.01505400	-2.29272200
C	4.17723900	1.15522700	0.69306300
C	-2.26461100	-2.43521000	-1.41792400
H	-1.44697400	-2.25089700	-2.12255800
H	3.97341400	-2.49675500	-0.45494000
H	-4.96439900	-0.41712600	0.45635600
H	4.21376000	-1.89114000	1.19811600
H	-4.15724300	-1.90144200	1.01887100
H	3.31649300	1.09162900	-2.37516400
H	-4.26111900	-0.68683200	-2.81853900
H	3.41528400	-0.65933000	-2.63496900
H	-2.73496700	0.24488400	-2.95239100
H	4.86651100	0.24249600	-2.10462800
H	-4.09430800	0.90452000	-2.00956000
H	5.37211500	-1.44584600	-0.08809000
H	-5.08149700	-1.91176600	-0.51121500
H	5.25005600	1.14935500	0.45576500
H	-3.06458500	-2.98955800	-1.92711700
H	4.04505400	0.97022800	1.76570500
H	-1.87489800	-3.03355200	-0.58764100
H	3.76741100	2.14554900	0.46566700
H	-0.24547500	3.54066200	0.51067700
H	1.44611200	3.51744100	1.06702000

H	-0.78542400	2.66694600	-1.89116800
H	0.57339600	2.23388000	-2.96457700
H	2.13993700	2.33308600	-1.00937900
H	1.37101700	3.88511600	-1.36230700
H	-3.59256800	1.90392300	2.13921600

Complex PMe3-A13

Fe	1.12502300	-0.55270100	0.02547300
Fe	-1.45764700	0.29707700	0.49827400
S	0.46810400	1.17973700	1.48862200
S	-0.19744000	0.56596400	-1.52170700
P	3.33276100	-0.01252200	-0.25112600
P	-2.99341200	-0.68654100	-0.74048500
O	1.18840200	-2.42064500	2.25877600
O	-1.43312600	-1.66443000	2.68031000
O	1.15713100	-2.81638700	-1.81978400
O	-3.23983000	2.44221700	1.34660400
C	1.27965800	-1.64378700	1.35749600
C	-1.44155400	-0.89760800	1.64120800
C	1.13422200	-1.90137000	-1.07915800
C	-2.49471200	1.65438900	0.87988000
C	0.86023300	2.83755100	0.74649100
C	0.30019800	2.34378800	-1.71779200
C	0.06929300	3.23992300	-0.50065600
C	4.38779500	-1.51615500	-0.50158000
C	-4.50190100	-1.22591900	0.17824600
C	3.94384400	1.06296100	-1.63973700
C	-3.69877100	0.33304500	-2.10936800
C	4.16774200	0.75766600	1.21670200
C	-2.44923300	-2.21738700	-1.61354500
H	-1.64298400	-1.96838300	-2.31208000
H	4.08237800	-2.03090700	-1.42001200
H	-4.95711000	-0.35903100	0.67100700
H	4.24549800	-2.20521300	0.33931700
H	-4.22292500	-1.95143700	0.95082500
H	3.59038100	2.09173400	-1.50297300
H	-4.45363500	-0.23394000	-2.67045600
H	3.55030000	0.68527400	-2.59102700
H	-2.88804300	0.62933600	-2.78371000
H	5.04198000	1.07263500	-1.68564600
H	-4.15968300	1.23646800	-1.69373500
H	5.45133100	-1.25037700	-0.57276600
H	-5.23339300	-1.68222600	-0.50122100
H	5.24759300	0.87207400	1.04898900
H	-3.28293600	-2.66980100	-2.16748400
H	4.00314300	0.11932500	2.09261700
H	-2.06040200	-2.93628100	-0.88403000
H	3.73206400	1.73965000	1.43226200
H	0.36864100	4.26686500	-0.77198700
H	-1.00193900	3.27306000	-0.26557200

H	0.68343000	3.56028000	1.55230400
H	1.93756400	2.82728800	0.52918800
H	-0.28314800	2.71029300	-2.57191800
H	1.35803700	2.33967500	-2.00890600
H	-0.60028200	-2.22406100	2.69674300

Complex PMe3-A14

Fe	1.10350000	-0.53694500	0.04806800
Fe	-1.48180800	0.32225900	0.49549200
S	0.43461600	1.18414300	1.52152400
S	-0.20016800	0.57905500	-1.50893100
P	3.32642400	-0.10482000	-0.25408600
P	-3.00815200	-0.66468300	-0.75356200
O	1.04150800	-2.35979800	2.31988400
O	-1.57337500	-1.55663500	2.74968500
O	1.10290900	-2.84439900	-1.74260000
O	-3.12952100	2.64940500	1.11660900
C	1.17958000	-1.61037200	1.40275500
C	-1.56098300	-0.81254000	1.69409500
C	1.09212100	-1.90922900	-1.02696500
C	-2.43391400	1.77526800	0.73459800
C	0.70665700	2.88105200	0.82991500
C	0.14891200	2.39731500	-1.64233600
C	1.15477300	2.94983400	-0.63148100
C	4.36150400	-1.62899800	-0.04955500
C	-4.55498900	-1.13687900	0.13877100
C	3.94867200	0.49936300	-1.89792100
C	-3.65431200	0.32248200	-2.17506600
C	4.15258400	1.04310200	0.94660900
C	-2.48168900	-2.23574900	-1.56503400
H	-1.65371100	-2.02887600	-2.25163400
H	4.05963100	-2.38109900	-0.78813200
H	-5.00081600	-0.24174800	0.58783300
H	4.20310000	-2.04781400	0.95125800
H	-4.31565900	-1.84119000	0.94353800
H	3.53516300	1.48806200	-2.12764500
H	-4.40000300	-0.25103300	-2.74183200
H	3.61329400	-0.19664300	-2.67631600
H	-2.81784100	0.58419000	-2.83214700
H	5.04583500	0.55863900	-1.91810400
H	-4.11455300	1.24559000	-1.80422300
H	5.42907800	-1.40609000	-0.18176800
H	-5.27935200	-1.59856000	-0.54483700
H	5.24071200	1.07160900	0.79789800
H	-3.31277600	-2.68531000	-2.12519500
H	3.93494000	0.69965700	1.96479300
H	-2.12826500	-2.94093600	-0.80474100
H	3.75050600	2.05677100	0.83848000
H	-0.23300600	3.42911500	0.97648800
H	1.46765100	3.33468500	1.47822200

H	-0.81956400	2.90547800	-1.54359400
H	0.52022100	2.54987800	-2.66378800
H	2.10894100	2.41840500	-0.74660400
H	1.34503000	4.00782800	-0.88045300
H	-0.75349500	-2.13388200	2.77424700

Complex PMe3-A15

Fe	1.05114600	-0.45170600	0.25853800
Fe	-1.37058100	0.33941400	0.33394700
S	0.36662200	1.52030200	1.24659600
S	-0.06165200	0.32452900	-1.62440700
P	3.15641400	-0.21652500	-0.39987600
P	-2.84786700	-1.02849200	-0.67582900
O	1.74403900	-1.41900000	2.92649700
O	-1.37898000	-0.20866600	3.16252200
O	0.91771300	-3.22740800	-0.62414000
O	-3.29821100	2.48173400	0.73233600
C	1.45409200	-1.02525800	1.85864900
C	-1.56655600	-0.42255200	1.88498000
C	0.94264100	-2.09757200	-0.29485500
C	-2.53907900	1.61110200	0.48957600
C	0.67850900	3.07605700	0.28678100
C	0.52451800	2.03435200	-2.06037400
C	0.05553700	3.13788000	-1.11040400
C	4.30336800	-1.47053200	0.33565800
C	-4.34049000	-1.39482000	0.34836400
C	3.49961600	-0.44919500	-2.20644500
C	-3.57257300	-0.35801900	-2.23984700
C	4.06866100	1.35523300	-0.02569700
C	-2.29017500	-2.71017800	-1.19273800
H	-1.48308500	-2.61862300	-1.92723800
H	3.94659500	-2.47904900	0.09528700
H	-4.85535800	-0.45988200	0.59827500
H	4.32116200	-1.36388100	1.42630400
H	-4.01729200	-1.87126900	1.28091100
H	2.96328500	0.29997000	-2.79863600
H	-4.29715500	-1.05977500	-2.67386700
H	3.13977300	-1.43918000	-2.50965600
H	-2.76284900	-0.17989600	-2.95652000
H	4.57560200	-0.37549800	-2.41442500
H	-4.07210600	0.59584300	-2.03323700
H	5.32210400	-1.34710700	-0.05568400
H	-5.03216600	-2.05944100	-0.18567100
H	5.13036200	1.27084800	-0.29613000
H	-3.12075900	-3.27623000	-1.63654800
H	3.98606200	1.57471800	1.04523600
H	-1.90410400	-3.25043900	-0.32209000
H	3.62807900	2.18808600	-0.58545900
H	0.31873700	4.11446900	-1.55090900
H	-1.03909400	3.10703700	-1.03024900

H	0.27781700	3.87980400	0.91648000
H	1.76817700	3.19559400	0.23878700
H	0.13752000	2.21612800	-3.07068500
H	1.62124500	2.01052200	-2.11875300
H	-0.93986600	0.67254900	3.32079100

Complex PMe3-A16

Fe	1.15453900	-0.52012500	0.14696000
Fe	-1.49853800	0.26614600	0.48779500
S	0.36495400	1.17745800	1.54783400
S	-0.13722800	0.49236300	-1.49867700
P	3.31523400	-0.07270500	-0.35032100
P	-2.99087900	-0.67885200	-0.84342800
O	1.75092400	-2.08844900	2.54029300
O	-2.06119300	-1.44495600	2.80770600
O	1.04181500	-3.02160300	-1.35893800
O	-3.16966700	2.57184600	1.05578200
C	1.52138000	-1.44472800	1.58369300
C	-1.73446600	-0.83792100	1.69784300
C	1.07769200	-2.00373000	-0.76656200
C	-2.45926900	1.68613800	0.72199300
C	0.60586800	2.87289400	0.83773600
C	0.14317600	2.32381000	-1.64520300
C	1.09447500	2.92860300	-0.61089300
C	4.39777800	-1.57232700	-0.21778800
C	-4.57940400	-1.17622900	-0.03927600
C	3.75499500	0.49862000	-2.06223200
C	-3.57487100	0.34361500	-2.26676200
C	4.25564100	1.11437700	0.72634500
C	-2.42455700	-2.23451900	-1.65444700
H	-1.56339100	-2.01574900	-2.29468300
H	4.03428100	-2.34883800	-0.90120800
H	-5.04828900	-0.29755900	0.41942100
H	4.35707400	-1.96820600	0.80389000
H	-4.37737100	-1.91519700	0.74463300
H	3.31476100	1.48109800	-2.26756800
H	-4.29367000	-0.21575000	-2.88040400
H	3.34469600	-0.21507800	-2.78657700
H	-2.71019400	0.61994500	-2.87970400
H	4.84414500	0.56029400	-2.19418300
H	-4.05251400	1.25740300	-1.89463200
H	5.44035700	-1.33301100	-0.46855500
H	-5.27165300	-1.61096300	-0.77209800
H	5.31869900	1.15531400	0.45054800
H	-3.22818500	-2.67232100	-2.26196400
H	4.16720000	0.78580200	1.76880500
H	-2.10860500	-2.94939200	-0.88712200
H	3.82962400	2.12138300	0.65103200
H	-0.34987300	3.40068500	0.95008600
H	1.33838600	3.35439900	1.49859800

H	-0.84598400	2.79691400	-1.58715300
H	0.54306000	2.47946300	-2.65555400
H	2.06832800	2.42662700	-0.68224900
H	1.25906600	3.98741000	-0.87489000
H	-2.54056800	-0.85132500	3.43218500

Complex PMe3-A17

Fe	1.07213200	-0.44976600	0.18029600
Fe	-1.30769000	0.27284700	0.44966600
S	0.38312200	1.27281000	1.65098500
S	-0.08025600	0.80946700	-1.43968200
P	3.13213200	-0.11422500	-0.54883000
P	-2.74369100	-0.73566700	-0.91975800
O	1.89741100	-1.98955400	2.50487300
O	-1.65143300	-1.85238200	2.41824300
O	0.58136100	-3.25483900	-0.40808500
O	-3.34378300	2.12393900	1.41631600
C	1.55977200	-1.32782400	1.58958000
C	-1.49351200	-0.98144600	1.64214700
C	0.72465700	-1.95781300	-0.61236400
C	-2.53837400	1.39467000	0.96056100
C	0.64604500	3.01748600	1.07047200
C	0.41776100	2.60159600	-1.45331000
C	-0.03479400	3.41404000	-0.23995800
C	4.30894400	-1.49511900	-0.18209200
C	-4.19703000	-1.52263700	-0.08946700
C	3.32588800	0.04376100	-2.38257300
C	-3.55185600	0.42645500	-2.11174600
C	4.07714000	1.34765300	0.08699800
C	-2.15267000	-2.07361200	-2.04536300
H	-1.29366800	-1.70824700	-2.61941200
H	3.93621900	-2.42067300	-0.63661200
H	-4.72846500	-0.77251800	0.50794600
H	4.38209500	-1.64661000	0.90093900
H	-3.85433100	-2.31808500	0.58157200
H	2.80900200	0.93915500	-2.74344400
H	-4.28301700	-0.10046500	-2.73940500
H	2.86175000	-0.82786600	-2.85746600
H	-2.77696500	0.87014200	-2.74690500
H	4.38677200	0.09701500	-2.66256200
H	-4.05751500	1.22860100	-1.56175900
H	5.30666500	-1.27516400	-0.58485000
H	-4.88585000	-1.94723300	-0.83120400
H	5.10540900	1.35565400	-0.30017900
H	-2.95520900	-2.37510800	-2.73248500
H	4.10282000	1.31202900	1.18226000
H	-1.83070800	-2.94003400	-1.45901100
H	3.57734300	2.27466900	-0.21557700
H	0.18945200	4.47729900	-0.43099800
H	-1.12488200	3.33202800	-0.13273900

H	0.27307700	3.64377300	1.89069900
H	1.73256500	3.16213300	1.00642700
H	-0.02561100	3.00994900	-2.36996100
H	1.51037500	2.64175200	-1.56488200
H	0.67442900	-3.52094800	0.54049100

Complex PMe3-A18

Fe	1.01070400	-0.47997000	0.10052900
Fe	-1.33592100	0.34566900	0.45038500
S	0.43250000	1.27224600	1.58349600
S	-0.17611500	0.80375900	-1.48302000
P	3.15220100	-0.19388500	-0.38259900
P	-2.82093300	-0.73055000	-0.82204700
O	1.46296100	-2.30110100	2.31365700
O	-1.96677400	-1.40961300	2.70065800
O	0.76394800	-3.25552100	-0.75199100
O	-3.13624700	2.55060000	1.08118000
C	1.28256800	-1.49773500	1.46733200
C	-1.69442700	-0.70186900	1.80337900
C	0.76905100	-1.93549000	-0.81783100
C	-2.43875200	1.65164600	0.77633900
C	0.73791600	2.98776600	0.95212100
C	0.24663500	2.60900700	-1.54889500
C	1.23326200	3.10384900	-0.49034500
C	4.14012100	-1.75998400	-0.33560700
C	-4.25555400	-1.48252800	0.07211700
C	3.57411800	0.42031500	-2.07837700
C	-3.66483100	0.36651700	-2.04982600
C	4.16450100	0.88132200	0.73808200
C	-2.25126000	-2.12182100	-1.89275700
H	-1.40578600	-1.78360900	-2.50273500
H	3.73044100	-2.47370600	-1.05988700
H	-4.76828000	-0.71065100	0.65814300
H	4.08711600	-2.20689400	0.66425000
H	-3.90217100	-2.25819400	0.76039100
H	3.17270600	1.42739700	-2.23350900
H	-4.39680700	-0.19879400	-2.64181400
H	3.10788200	-0.24766400	-2.81172500
H	-2.90725500	0.79458100	-2.71562900
H	4.66141800	0.43475200	-2.23509000
H	-4.17554700	1.18325400	-1.52678100
H	5.19204700	-1.56312400	-0.58244100
H	-4.96448100	-1.92538800	-0.63939500
H	5.22076000	0.88748300	0.43588900
H	-3.06577900	-2.46288200	-2.54640900
H	4.08285600	0.49408800	1.76044200
H	-1.91263200	-2.95576500	-1.26944000
H	3.78183600	1.90718800	0.73443500
H	-0.19824700	3.54467100	1.08972900
H	1.48137800	3.41041200	1.64039100

H	-0.70344000	3.15434300	-1.47949800
H	0.65860700	2.77271300	-2.55325300
H	2.18099900	2.55863900	-0.59494300
H	1.45291500	4.16579000	-0.69506700
H	0.92275400	-3.60723800	0.15984000

Complex PMe3-A19

Fe	1.04230800	-0.48711400	0.07781700
Fe	-1.30598900	0.32629800	0.45349200
S	0.49187800	1.30882300	1.49121000
S	-0.19147800	0.72894700	-1.52908800
P	3.19036100	-0.06306000	-0.25489000
P	-2.81068200	-0.79919900	-0.73409000
O	1.40786300	-2.77472000	1.85979700
O	-1.76930500	-1.44872000	2.72590000
O	1.01727100	-2.95715300	-1.44406100
O	-3.25876400	2.34013500	1.24784900
C	1.29420000	-1.52857200	1.45528800
C	-1.54661800	-0.73692800	1.81801400
C	0.98474700	-1.91098300	-0.89017500
C	-2.48135600	1.54250600	0.86294900
C	0.86548900	2.96807600	0.74023700
C	0.21945300	2.53324800	-1.71033400
C	0.01420700	3.38780700	-0.46033800
C	4.21714300	-1.56134200	-0.61410800
C	-4.21000400	-1.52589900	0.23265600
C	3.70291000	1.05733800	-1.63971700
C	-3.69715400	0.23192200	-1.98829200
C	4.09216800	0.65980500	1.19012700
C	-2.24924900	-2.23127500	-1.75550800
H	-1.48475600	-1.89453600	-2.46403500
H	3.86599800	-2.04131700	-1.53479600
H	-4.71050000	-0.73588400	0.80500500
H	4.11969500	-2.27947300	0.20872200
H	-3.82793700	-2.27189000	0.93812000
H	3.34571200	2.07523700	-1.44740000
H	-4.43361600	-0.36974300	-2.53718600
H	3.25848500	0.70466000	-2.57751600
H	-2.96096300	0.64057000	-2.68914400
H	4.79643400	1.08110900	-1.74275900
H	-4.20777100	1.06326000	-1.48884300
H	5.27552700	-1.29190000	-0.72826900
H	-4.93694500	-2.00064600	-0.43900500
H	5.16211400	0.77795300	0.97087100
H	-3.09337300	-2.66218300	-2.31121600
H	3.96321800	-0.00519200	2.05157200
H	-1.81006500	-2.99995400	-1.11135400
H	3.66471200	1.63334100	1.45289200
H	0.27068200	4.43069200	-0.71354700
H	-1.04691600	3.38238400	-0.17987400

H	0.72617700	3.68096500	1.56222700
H	1.93245400	2.96886900	0.47538800
H	-0.42575800	2.89081000	-2.52289500
H	1.25746200	2.58881800	-2.06149200
H	1.24191900	-3.44572700	1.15057300

Complex PMe3-A20

Fe	1.01420600	-0.48001700	0.12057800
Fe	-1.33568500	0.35485200	0.44914800
S	0.43367600	1.31414300	1.54999200
S	-0.17587900	0.75307600	-1.49593100
P	3.16786300	-0.17435400	-0.28732100
P	-2.83254900	-0.74746600	-0.78046600
O	1.27143900	-2.71649900	1.98707200
O	-1.98216400	-1.31212200	2.76354000
O	0.89296600	-3.02351500	-1.26080600
O	-3.13343300	2.57683600	1.01968800
C	1.20711900	-1.48494000	1.53322100
C	-1.69462300	-0.64535500	1.84032600
C	0.90295500	-1.94053000	-0.77949000
C	-2.43488200	1.67093400	0.73792000
C	0.71640600	3.01318900	0.86445000
C	0.24152400	2.55730300	-1.62705900
C	1.21733300	3.09291000	-0.57915800
C	4.17405100	-1.72472100	-0.17050700
C	-4.25458800	-1.48817500	0.14262200
C	3.67660300	0.42713100	-1.96632600
C	-3.69208500	0.31713500	-2.02591700
C	4.09803600	0.92937900	0.87257500
C	-2.27057000	-2.16156200	-1.82745100
H	-1.47976200	-1.82154000	-2.50501300
H	3.79384900	-2.47042000	-0.87845100
H	-4.76217100	-0.70912600	0.72358300
H	4.10110800	-2.13632300	0.84298100
H	-3.89099100	-2.25272300	0.83796400
H	3.25728800	1.41914900	-2.16471900
H	-4.41783200	-0.26740000	-2.60670500
H	3.28260300	-0.26456900	-2.72019900
H	-2.94074300	0.74305700	-2.70017900
H	4.77077500	0.47263700	-2.05470300
H	-4.21194600	1.13648300	-1.51621800
H	5.22858000	-1.52006600	-0.39865300
H	-4.97066100	-1.94284300	-0.55416600
H	5.17250800	0.93169500	0.64324900
H	-3.10718000	-2.55771200	-2.41910800
H	3.94098600	0.56372400	1.89376300
H	-1.86607700	-2.95817200	-1.19482900
H	3.71420200	1.95339700	0.81664900
H	-0.22493600	3.56548900	0.98139700
H	1.45287800	3.46720300	1.54039100

H	-0.71283800	3.09830500	-1.58272900
H	0.65912000	2.68533100	-2.63419700
H	2.17123700	2.55501000	-0.66319800
H	1.42705700	4.15054900	-0.81430200
H	1.10276800	-3.40885000	1.29916400

Complex PMe3-A21

Fe	1.10864500	-0.60672500	0.03912400
Fe	-1.40632300	0.39137800	0.44946000
S	0.54020600	1.20275800	1.44854200
S	-0.15439800	0.54762100	-1.54658100
P	3.33164900	-0.11410600	-0.15627400
P	-2.93604000	-0.67723600	-0.72560400
O	0.62037700	-2.35652200	2.33606400
O	-1.76060300	-1.44720300	2.68148900
O	1.13678900	-2.81700300	-1.87440300
O	-3.18764300	2.62286700	1.11501900
C	0.91370700	-1.63432300	1.33450000
C	-1.66544300	-0.66686500	1.76246100
C	1.12770900	-1.92313400	-1.11264200
C	-2.44140900	1.79861800	0.71931200
C	1.06072700	2.79961500	0.65556400
C	0.47719800	2.27446400	-1.79204000
C	0.29590100	3.21859300	-0.60248400
C	4.32519500	-1.66491900	-0.36355500
C	-4.47136400	-1.12377300	0.20155900
C	4.07733400	0.94388800	-1.49217300
C	-3.61381200	0.24350900	-2.17869200
C	4.11050300	0.59123900	1.37170100
C	-2.42632500	-2.28273400	-1.48385400
H	-1.61208800	-2.10539000	-2.19469700
H	4.05557200	-2.15434900	-1.30657100
H	-4.92754900	-0.21351300	0.60805600
H	4.09277100	-2.35264200	0.45778600
H	-4.22129600	-1.78655300	1.03757200
H	3.77145000	1.98870500	-1.36339800
H	-4.36492500	-0.35897700	-2.70687900
H	3.72156600	0.59737300	-2.46985300
H	-2.79135100	0.48538800	-2.86026500
H	5.17536500	0.89326000	-1.47339800
H	-4.07381300	1.17768900	-1.83645800
H	5.40203700	-1.44862500	-0.36257900
H	-5.19273100	-1.62623100	-0.45582600
H	5.19967500	0.67326900	1.25510600
H	-3.26791500	-2.75411400	-2.00946400
H	3.88388000	-0.06820800	2.21727700
H	-2.06110800	-2.95907200	-0.70282300
H	3.69545400	1.57945700	1.59729200
H	0.65719100	4.21765800	-0.90092800
H	-0.77195300	3.32224900	-0.37182800

H	0.94001400	3.55674800	1.44023800
H	2.13343100	2.70540000	0.43498400
H	-0.06686100	2.65499100	-2.66586400
H	1.53634700	2.18377400	-2.06408100
H	-0.37149400	-2.20737300	2.60692000

Complex PMe3-A22

Fe	1.07247700	-0.57520100	0.02440800
Fe	-1.41545100	0.41508400	0.44930900
S	0.51574900	1.25063300	1.44858200
S	-0.18467200	0.56720100	-1.55447300
P	3.32398300	-0.21023600	-0.10920600
P	-2.94331900	-0.68962400	-0.70570600
O	0.45505400	-2.28863500	2.32682100
O	-1.86948400	-1.28486000	2.76801700
O	1.16031100	-2.81147800	-1.85530700
O	-3.07138600	2.79462500	0.87420500
C	0.77215800	-1.58239900	1.32102700
C	-1.74506600	-0.54284200	1.82083600
C	1.12385100	-1.90498100	-1.10890500
C	-2.37303900	1.89153000	0.57578900
C	0.86541100	2.89742200	0.67496700
C	0.30001500	2.34410000	-1.77764400
C	1.33248400	2.87485200	-0.78230300
C	4.22739900	-1.77252400	0.31704500
C	-4.47689000	-1.13577700	0.22505600
C	4.15571200	0.23341100	-1.71006200
C	-3.63240600	0.20099500	-2.17252600
C	4.08644900	0.97771200	1.09224600
C	-2.42175400	-2.30254400	-1.44086500
H	-1.60252100	-2.13091400	-2.14735000
H	3.95104700	-2.56263600	-0.39108600
H	-4.93931000	-0.22296500	0.61871000
H	3.93495700	-2.09592400	1.32254500
H	-4.22410500	-1.78527700	1.07054100
H	3.84304800	1.23021300	-2.04173300
H	-4.37374300	-0.41996700	-2.69304300
H	3.85565700	-0.49037600	-2.47736100
H	-2.81323700	0.44622400	-2.85686700
H	5.24996000	0.21780600	-1.61097200
H	-4.10809500	1.13243800	-1.84426700
H	5.31553800	-1.62520600	0.28391000
H	-5.19425200	-1.65141700	-0.42651200
H	5.18255500	0.90962700	1.07728100
H	-3.25713600	-2.78291400	-1.96821600
H	3.71915500	0.74373000	2.09803500
H	-2.06129400	-2.96905800	-0.64928800
H	3.78972100	2.00477000	0.85185600
H	-0.05646500	3.48559500	0.77690300
H	1.63435400	3.35790800	1.30881000

H	-0.63108700	2.92354900	-1.71405600
H	0.68810500	2.41771600	-2.80136700
H	2.25228900	2.27936100	-0.86354100
H	1.59179500	3.90652800	-1.07493800
H	-0.51871700	-2.09417200	2.63541900

Complex PMe3-B1

Fe	-1.37603100	-0.58613200	-0.06790700
Fe	1.34898300	-0.58452700	0.00748500
S	0.03702400	0.45469800	-1.65669500
S	-0.06788900	0.57545200	1.52774200
P	-3.30914300	0.63760000	0.07185500
P	3.32372000	0.57964800	0.05364700
O	-2.31413700	-2.32774600	-2.21065900
O	2.12263600	-2.35459300	2.19528500
O	-2.26512400	-2.48963600	1.95652500
O	2.29266700	-2.50755400	-1.97465300
C	-1.95298800	-1.61605600	-1.35273800
C	1.83595300	-1.62850700	1.32114000
C	-1.93176400	-1.70179400	1.15492700
C	1.93141000	-1.72004700	-1.18553900
C	-0.00027300	2.29411600	-1.45861900
C	-0.19759300	2.38126500	1.12403800
C	0.52785800	2.85600600	-0.13665200
C	-3.77866800	1.21354400	1.77180600
C	4.76898800	-0.57852600	0.13997500
C	-3.67159000	2.14397300	-0.95819300
C	3.81999500	1.60299500	-1.41510400
C	-4.78488000	-0.40138800	-0.35146500
C	3.70315200	1.68387900	1.49799100
H	3.01574200	2.53609700	1.53065700
H	-3.02812300	1.90559000	2.16899900
H	4.76540500	-1.24172200	-0.73284900
H	-3.82094100	0.34569600	2.44020700
H	4.69625300	-1.19659900	1.04248600
H	-4.72568800	2.43991200	-0.86303000
H	4.84828200	1.97498700	-1.31116700
H	-3.45903800	1.92404400	-2.01135500
H	3.14545700	2.45598600	-1.54819500
H	-3.04403800	2.98559200	-0.64333900
H	3.75562200	0.97537900	-2.31213300
H	-4.75882600	1.70888500	1.76348900
H	5.71594800	-0.02280100	0.16248300
H	-5.71601700	0.16096000	-0.20090800
H	4.73464600	2.05840400	1.45215200
H	-4.80069800	-1.29606000	0.28208200
H	3.57180100	1.10617400	2.42070900
H	-4.72447700	-0.72395100	-1.39728400
H	0.44889500	3.95603100	-0.18118000
H	1.59434300	2.62121600	-0.04460600

H	0.59231600	2.68947500	-2.29385800
H	-1.04095200	2.59896800	-1.61924500
H	0.20134400	2.90448000	2.00228900
H	-1.26853500	2.61557300	1.05316200
H	-0.02621600	-1.52685700	-0.04927200

Complex PMe3-B2

Fe	-1.26411800	-0.53141100	0.19661900
Fe	1.40251800	-0.60132600	0.04477800
S	0.02065600	0.31617500	-1.61297900
S	0.18014800	0.73590700	1.57916000
O	-2.48704700	-1.60920300	2.62149500
C	-1.98888200	-1.17793400	1.65564000
C	2.11248600	-1.70992800	-1.07760200
C	-0.03599200	2.16782800	-1.51219100
C	0.14007600	2.50812200	1.04728100
C	0.70678300	2.81230800	-0.34072600
H	-0.90093900	2.84134900	1.12280700
H	0.71354600	3.04986600	1.81068500
H	0.68482200	3.90652700	-0.48481300
H	1.76031000	2.50946500	-0.37598800
H	0.39275200	2.51508600	-2.46073100
H	-1.09305700	2.46618000	-1.49749900
C	-1.97231700	-1.76116000	-0.83721300
O	-2.45370800	-2.59596700	-1.50151900
C	0.28441600	-1.95774800	0.54883500
O	0.16514400	-3.08212600	0.91152000
O	2.59128700	-2.46591600	-1.83804900
P	3.41862300	0.43758700	0.18221300
P	-3.16004900	0.77884400	-0.21135100
C	-3.58158100	1.00513100	-2.00351300
H	-3.64626100	0.01901000	-2.47770100
H	-4.54386400	1.52238600	-2.11542400
H	-2.80211500	1.57453600	-2.52030100
C	-3.46968300	2.47483400	0.48594100
H	-4.51462700	2.77348800	0.32382200
H	-3.26725900	2.46795500	1.56366300
H	-2.81712300	3.21538200	0.01028200
C	-4.68527100	-0.09246000	0.38232100
H	-4.66981500	-0.17611700	1.47482700
H	-5.58625500	0.45522200	0.07603600
H	-4.72176500	-1.10385800	-0.03876400
C	4.12688000	1.34248300	-1.28042600
H	5.15371500	1.67836500	-1.08178900
H	4.13378500	0.66127500	-2.13975900
H	3.51171400	2.21128700	-1.53949300
C	3.69227400	1.61213200	1.58895600
H	4.75706100	1.86597900	1.67627000
H	3.11852300	2.53444800	1.44739400
H	3.35439600	1.13248200	2.51463800

C	4.76654900	-0.79180400	0.49675000
H	5.74407500	-0.29446700	0.54712400
H	4.56669800	-1.30664400	1.44298300
H	4.78365200	-1.54042900	-0.30315800
H	2.09832100	-1.17250500	1.25484100

Complex PMe3-B3

Fe	-1.25156700	-0.52000600	0.14633200
Fe	1.44069600	-0.63356800	0.02833400
S	0.06463500	0.18251300	-1.68947500
S	0.19564900	0.80890700	1.45361800
O	-2.40376800	-1.61146700	2.60232000
C	-1.92645700	-1.17766000	1.62556700
C	2.21629000	-1.71827400	-1.07439400
C	0.21735400	2.02763600	-1.76999300
C	0.36296000	2.52640900	0.77320300
C	-0.30477700	2.82183100	-0.57136200
H	-0.05353900	3.18673600	1.54462400
H	1.43911600	2.73598200	0.70679600
H	-1.38463700	2.66409500	-0.47665300
H	-0.16231100	3.89475900	-0.78825600
H	1.28129500	2.23610900	-1.93707800
H	-0.32458400	2.32871500	-2.67591900
C	-1.93396800	-1.78726700	-0.86085700
O	-2.40217200	-2.63287600	-1.52079200
C	0.37240300	-1.99643500	0.54091600
O	0.20987800	-3.10275400	0.93010200
O	2.72829800	-2.45636600	-1.82915700
P	3.38293200	0.51997700	0.26902100
P	-3.17823600	0.73781900	-0.12033800
C	-3.54307600	1.47934500	-1.78150700
H	-3.46128000	0.68779400	-2.53584700
H	-4.55596800	1.90281900	-1.81081300
H	-2.82073300	2.26298500	-2.03147500
C	-3.55421800	2.10234400	1.08240700
H	-4.55370000	2.52127500	0.90401700
H	-3.51595800	1.68749400	2.09681600
H	-2.81347400	2.90618600	1.01683500
C	-4.69123100	-0.31012600	0.10485300
H	-4.70057500	-0.74308300	1.11160900
H	-5.60019300	0.29015100	-0.03338700
H	-4.68993400	-1.12860600	-0.62395100
C	3.92690300	1.90696600	-0.85171700
H	4.95925100	2.21096400	-0.62914700
H	3.87393100	1.57012000	-1.89413900
H	3.27271300	2.77874500	-0.73552900
C	3.64962300	1.24781200	1.95100900
H	4.65331300	1.68724900	2.02158700
H	2.90183300	2.01564700	2.17465800
H	3.54497500	0.44915800	2.69345800

C	4.85628800	-0.59301700	0.14050400
H	5.77198400	-0.04981100	0.40800000
H	4.72563300	-1.44498000	0.81688900
H	4.95147600	-0.97658700	-0.88144500
H	2.16259500	-1.16033400	1.24894900

Complex PMe3-B4

Fe	1.23472300	-0.43498000	0.05403200
Fe	-1.23092000	-0.42328400	-0.04374800
S	0.00107400	0.76526700	1.61258900
S	0.13077900	0.81420100	-1.55750300
P	3.27097900	0.40670700	-0.05808400
P	-3.25719700	0.47605100	-0.05208400
O	1.84368400	-2.34338500	2.17852900
O	-1.82261000	-2.84470900	-1.55994900
O	1.74731000	-2.54238800	-1.90030100
O	-1.80980500	-2.57409500	1.81543300
C	1.59912700	-1.56379600	1.33571000
C	-1.53240400	-1.59108900	-1.29389300
C	1.54323900	-1.67361900	-1.13655100
C	-1.56812200	-1.65454200	1.11626300
C	0.03573500	2.60269300	1.37070300
C	0.28256900	2.62630300	-1.18650000
C	-0.48942900	3.12799000	0.03452100
C	3.81241600	1.03689000	-1.71494200
C	-4.62275700	-0.77385000	-0.05381900
C	3.75422100	1.80523200	1.06048100
C	-3.75623600	1.53142100	1.38765900
C	4.61013000	-0.81892200	0.30745400
C	-3.72189600	1.49987500	-1.52468800
H	-3.08335900	2.38705400	-1.59223400
H	3.17327000	1.86749200	-2.03355100
H	-4.54384500	-1.41067600	0.83509800
H	3.71168600	0.23286300	-2.45301200
H	-4.53766700	-1.40691100	-0.94475400
H	4.82677700	2.02677300	0.97191100
H	-4.79861700	1.86583500	1.29488800
H	3.52531600	1.53869800	2.09884400
H	-3.10279300	2.40681500	1.46348000
H	3.18780900	2.70676300	0.80037000
H	-3.64478200	0.94506500	2.30722500
H	4.85770300	1.37337900	-1.68529100
H	-5.60444200	-0.28154800	-0.05576200
H	5.60237900	-0.36010000	0.20212600
H	-4.77372800	1.81386600	-1.47716000
H	4.53181700	-1.66480600	-0.38574900
H	-3.55970300	0.89728900	-2.42579600
H	4.49555000	-1.20051700	1.32851900
H	-0.43853600	4.23045300	0.05306500
H	-1.54929700	2.86108500	-0.07362000

H	-0.56549500	3.00278500	2.19778400
H	1.07100100	2.92838700	1.53397400
H	-0.07673500	3.12930100	-2.09349000
H	1.35157400	2.85729800	-1.08120800
H	-1.81932800	-3.43556200	-0.76595300

Complex PMe3-B5

Fe	-1.27587400	-0.40936400	-0.07463100
Fe	1.18925200	-0.42241600	0.09797300
S	0.11920100	0.70983900	-1.63346600
S	-0.09315000	0.88123400	1.52845000
P	-3.26569400	0.52775800	0.15024200
P	3.26573300	0.31382400	-0.06712800
O	-2.09650600	-2.25071200	-2.16911200
O	1.83540300	-2.09664500	2.40221600
O	-1.55427800	-3.02076200	1.18279400
O	1.42662500	-2.78176700	-1.59552500
C	-1.74828800	-1.47466300	-1.35174700
C	1.56524000	-1.41500800	1.48565400
C	-1.42537100	-1.71859400	1.06036300
C	1.36063200	-1.80194100	-0.95017900
C	0.18444000	2.55672800	-1.44906700
C	-0.08095600	2.68961400	1.10528700
C	0.73161500	3.09014200	-0.12594500
C	-3.67926900	1.11830100	1.85449600
C	4.51624700	-1.05230400	-0.09312000
C	-3.69566800	2.00192500	-0.89203400
C	3.73960000	1.25181200	-1.59409300
C	-4.69222600	-0.59620400	-0.20613700
C	3.94315100	1.37272800	1.29785800
H	3.37480300	2.30568400	1.37904600
H	-2.98753100	1.91298100	2.15433400
H	4.32160900	-1.71247200	-0.94631100
H	-3.55500100	0.28416100	2.55407200
H	4.43649600	-1.64429500	0.82604000
H	-4.73501700	2.31709100	-0.72497000
H	4.82033000	1.44769000	-1.61621000
H	-3.56074500	1.75137200	-1.95072600
H	3.20267800	2.20550900	-1.63853300
H	-3.03055600	2.83829400	-0.64808000
H	3.45778300	0.66061700	-2.47279700
H	-4.71089600	1.49285300	1.90315400
H	5.53519800	-0.65061100	-0.17376100
H	-5.64835200	-0.08094600	-0.04381200
H	5.00227800	1.60985500	1.12698300
H	-4.63807200	-1.46878700	0.45543000
H	3.84391600	0.83220800	2.24661300
H	-4.64153600	-0.94606700	-1.24351500
H	0.76091200	4.19188500	-0.18271500
H	1.76823100	2.75138100	0.00301800

H	0.80248600	2.91153000	-2.28450400
H	-0.83792000	2.91792700	-1.61934000
H	0.31762500	3.18859200	1.99775700
H	-1.12853500	3.00317100	0.99127300
H	-1.54023200	-3.51034900	0.32321100

Complex PMe3-B6

Fe	-1.26471600	-0.45341300	-0.07832700
Fe	1.15020200	-0.45231400	0.17062500
S	0.03128800	0.72271300	-1.57640700
S	-0.24142800	0.75970600	1.61158600
P	-3.30949300	0.39006500	0.06572100
P	3.23583500	0.38191700	-0.24486700
O	-1.93056200	-2.21754900	-2.31034100
O	3.00155200	-1.48656800	2.17347300
O	-1.69288300	-2.68774600	1.75140100
O	1.46015200	-2.89002100	-1.39731000
C	-1.65335000	-1.50824300	-1.42007900
C	1.89412800	-1.34073400	1.66779600
C	-1.53926100	-1.76377300	1.04365900
C	1.34721000	-1.89394900	-0.78532200
C	0.00648100	2.55562100	-1.29446900
C	-0.28535300	2.58019200	1.26387600
C	0.52635500	3.05044600	0.05648100
C	-3.85931800	0.91034400	1.75595300
C	4.48150300	-0.94880000	-0.55025800
C	-3.75079100	1.87130300	-0.95869700
C	3.47834900	1.44140800	-1.74426300
C	-4.65417900	-0.79024200	-0.40636600
C	4.03232600	1.37371200	1.10037600
H	3.47118000	2.29859600	1.27601300
H	-3.20839100	1.70329200	2.13969100
H	4.18690600	-1.52517300	-1.43472500
H	-3.78401500	0.05462500	2.43664900
H	4.51325300	-1.61308700	0.31915300
H	-4.81948500	2.10858700	-0.86626300
H	4.54581500	1.65167300	-1.89536300
H	-3.51568900	1.67199200	-2.01063200
H	2.94165900	2.38993700	-1.63454600
H	-3.16908600	2.74041500	-0.63086700
H	3.08215700	0.91868600	-2.62192100
H	-4.89762600	1.26786100	1.73632000
H	5.47528000	-0.51162100	-0.71625200
H	-5.64286100	-0.33217500	-0.27070400
H	5.06723300	1.62571700	0.83251100
H	-4.58773200	-1.69015100	0.21645400
H	4.02819000	0.77285900	2.01561000
H	-4.53576300	-1.08948400	-1.45400300
H	0.51973400	4.15389700	0.04484600
H	1.57192700	2.73896900	0.18581200

H	0.61462300	2.97179400	-2.10805900
H	-1.02611300	2.88742800	-1.45999400
H	0.09254200	3.04854800	2.18098300
H	-1.34131300	2.86293100	1.15471700
H	1.01138200	-1.82428500	2.17926000

Complex PMe3-B7

Fe	-1.23004800	-0.41139100	-0.14814800
Fe	1.23935300	-0.44921000	0.09460700
S	0.21329500	0.67655000	-1.66296900
S	-0.13277600	0.77348200	1.48844400
P	-3.33223600	0.45172400	0.09743300
P	3.31004400	0.32504900	-0.03861300
O	-2.08095100	-2.19224400	-2.29905500
O	1.89319200	-2.12581900	2.39062300
O	-2.11537000	-1.88001900	1.86414900
O	1.47155700	-2.80455300	-1.60768200
C	-1.73122700	-1.47813600	-1.43258000
C	1.62926300	-1.43881100	1.47510600
C	-1.29900600	-1.96320800	0.94196600
C	1.40778400	-1.82667900	-0.96152200
C	0.17961100	2.51818600	-1.41892900
C	-0.15753600	2.59462000	1.12736400
C	0.67801300	3.04159600	-0.07250700
C	-3.75541500	1.11810100	1.76846900
C	4.58617300	-1.01587800	-0.08659200
C	-3.82483600	1.82620700	-1.04339100
C	3.77211500	1.30501300	-1.54220300
C	-4.67458300	-0.79006100	-0.16581700
C	3.95529900	1.37295800	1.35022600
H	3.36490800	2.29143600	1.44213200
H	-3.12912100	1.98605600	2.00068600
H	4.40545000	-1.66203600	-0.95342100
H	-3.54761700	0.33592700	2.50626600
H	4.51568500	-1.62787200	0.82015400
H	-4.87884600	2.09827900	-0.89626300
H	4.84923600	1.52041700	-1.55591200
H	-3.67528400	1.51212300	-2.08275300
H	3.21887400	2.25022800	-1.56553200
H	-3.20255800	2.70886400	-0.85466200
H	3.50298100	0.73017100	-2.43551200
H	-4.81323900	1.41015900	1.81726900
H	5.59747900	-0.59353300	-0.15672800
H	-5.66205600	-0.33644500	-0.00721600
H	5.00997600	1.63758400	1.19280400
H	-4.53469300	-1.61227200	0.54506200
H	3.86107100	0.81453500	2.28908700
H	-4.61692200	-1.19229800	-1.18330400
H	0.67487600	4.14447600	-0.10747300
H	1.72078200	2.73107900	0.07791400

H	0.79438500	2.92323400	-2.23334000
H	-0.85569100	2.83676400	-1.59924500
H	0.20718800	3.07313900	2.04472000
H	-1.20816400	2.88715600	0.99174600
H	-0.73740700	-2.90602200	0.78388000

Complex PMe3-C1

Fe	1.37798700	0.27376000	0.37593700
Fe	-1.40206200	0.30161600	0.28662800
S	-0.02250800	1.96951800	1.11255400
S	0.06583000	0.31159200	-1.59524100
P	2.49229200	-1.48997100	-0.37857700
P	-2.42135900	-1.56930800	-0.35104200
O	3.64444500	2.07743600	-0.14491500
O	-2.39682800	-0.11462700	2.99679300
O	2.17746100	-0.27990000	3.12643900
O	-3.74002600	1.95843500	-0.39824100
C	2.71985400	1.35805200	-0.03466600
C	-1.98963600	0.03580400	1.90636600
C	1.85793300	-0.06422600	2.01828400
C	-2.76269400	1.32557500	-0.23179200
C	0.11922900	3.37161200	-0.09464100
C	0.25631900	2.01378600	-2.30384200
C	-0.41517400	3.14047200	-1.51275300
C	4.06506300	-1.84932200	0.52631800
C	-4.26574200	-1.50470100	-0.20654200
C	1.66564000	-3.14385800	-0.35563400
C	-2.21697300	-2.14059600	-2.09919900
C	3.08019400	-1.37896100	-2.12869300
C	-2.04855200	-3.09032400	0.63490600
H	-0.97901000	-3.31318400	0.59320000
H	3.84925600	-2.06543600	1.57857200
H	-4.66254000	-0.72097100	-0.86113100
H	4.72159500	-0.97235700	0.48320000
H	-4.54888500	-1.26519100	0.82516700
H	2.33301200	-3.92800400	-0.73783600
H	-2.81102300	-3.04743100	-2.27444100
H	0.76429800	-3.10950600	-0.97702800
H	-1.16388700	-2.33993000	-2.32243600
H	1.37759700	-3.38750200	0.67349000
H	-2.55482800	-1.34626100	-2.77421500
H	4.58416700	-2.70765600	0.08007100
H	-4.70970000	-2.46925700	-0.48553500
H	3.65075500	-2.27557300	-2.40474700
H	-2.61547100	-3.95300600	0.25998100
H	3.72205500	-0.49707300	-2.23923600
H	-2.31922400	-2.91221700	1.68229400
H	2.21861700	-1.26687500	-2.79536900
H	1.33614600	2.19332400	-2.39801200
H	-0.17396600	1.96411200	-3.31171900

H	-0.26082100	4.07721500	-2.07479700
H	-1.49761100	2.97045000	-1.47894900
H	-0.42764300	4.19918600	0.37341600
H	1.18391300	3.63932300	-0.11808100
H	-0.02118200	-0.60516500	0.79546700

Complex PMe3-C2

Fe	1.34518500	0.30608600	0.28714500
Fe	-1.35179600	0.36338200	0.38639100
S	0.04727600	2.06988500	1.03722300
S	-0.06622600	0.28127800	-1.60907100
O	-0.15664100	-2.01081900	1.63837500
C	-2.20623500	0.54665900	1.88374500
C	2.65943300	1.33511600	-0.33856400
C	-0.29041200	-0.96771600	1.07061800
C	0.06121000	3.41115300	-0.24146700
C	0.02759200	1.96453500	-2.38428400
C	-0.61298300	3.09995600	-1.58097200
H	1.09265700	2.16370300	-2.56538000
H	-0.47245200	1.86464900	-3.35565200
H	-0.56261800	4.01345300	-2.19757100
H	-1.67484500	2.87472000	-1.41088000
H	-0.44579100	4.25536000	0.24165500
H	1.11507200	3.68555800	-0.38557400
O	3.59984700	1.99038000	-0.59480000
C	2.05188800	0.16368100	1.88017400
O	2.56762500	0.08907000	2.92910800
O	-2.79403800	0.70156900	2.88512600
P	2.30981400	-1.59048100	-0.43397000
P	-2.91360500	-0.94440100	-0.47848700
C	3.56501700	-1.33496700	-1.76695700
H	3.99367100	-2.29393900	-2.08682000
H	3.08442700	-0.84949700	-2.62448800
H	4.36895100	-0.68458200	-1.40545500
C	3.24788200	-2.53864600	0.84165200
H	2.56125900	-2.81955600	1.64846200
H	3.68912300	-3.44394100	0.40502400
H	4.04457600	-1.91564000	1.26330400
C	1.25170000	-2.91007700	-1.18075200
H	0.67875800	-2.49044500	-2.01484300
H	1.88397000	-3.72845600	-1.54980600
H	0.56384800	-3.29879200	-0.42369600
C	-3.31729700	-0.64447400	-2.25703700
H	-2.42758600	-0.81421600	-2.87218100
H	-4.12621100	-1.31115500	-2.58346500
H	-3.63178000	0.39840700	-2.37633000
C	-2.68308000	-2.78116800	-0.43862100
H	-2.46820900	-3.10379800	0.58619900
H	-3.58236700	-3.29501500	-0.80364400
H	-1.83288400	-3.05807400	-1.07177400

C	-4.57359000	-0.76102700	0.31379700
H	-4.87392100	0.29218300	0.27927100
H	-5.32043800	-1.37228800	-0.20922200
H	-4.52644500	-1.07273400	1.36315000
H	-2.41991000	1.34279900	-0.19622800

Complex PMe3-C3

Fe	1.51750000	0.37024100	0.24384800
Fe	-1.46973400	0.34343100	0.41064700
S	0.07086000	2.03064800	0.88304700
S	-0.07610400	-0.00433000	-1.47620800
O	-0.59655500	-1.84881100	2.16932700
C	-2.50954400	0.97962900	1.64785700
C	2.89679800	1.33300000	-0.30934500
C	-0.80527100	-0.95595600	1.43361000
C	-0.14066300	3.24714300	-0.50331900
C	-0.29256500	1.55149900	-2.47167400
C	0.36260600	2.80329500	-1.88149300
H	0.14527000	1.33662900	-3.45466700
H	-1.37385900	1.69310100	-2.58955300
H	1.45009900	2.66286300	-1.84632300
H	0.17531900	3.63427300	-2.58296300
H	-1.21103400	3.48632300	-0.52757800
H	0.41211800	4.13964900	-0.18466700
O	3.85814000	1.98236600	-0.51629500
C	2.04951900	0.18997800	1.90194900
O	2.46982600	0.09778100	2.99518400
O	-3.22145700	1.45921800	2.44288700
P	2.48403100	-1.54552800	-0.32293100
P	-3.01554800	-0.95127000	-0.50629100
C	3.05993500	-1.60359100	-2.08006300
H	3.52462900	-2.57217100	-2.30795400
H	2.20131900	-1.44446600	-2.74178000
H	3.78854800	-0.80343800	-2.25352000
C	4.01857700	-1.97850900	0.61065000
H	3.78135400	-2.10934600	1.67213300
H	4.46361200	-2.90381200	0.22220500
H	4.74579900	-1.16339000	0.51845700
C	1.49586500	-3.10468400	-0.20500200
H	0.60510900	-3.01121700	-0.83609900
H	2.08791300	-3.96463400	-0.54661100
H	1.17638200	-3.26986200	0.82921200
C	-3.59303500	-0.41540300	-2.17546300
H	-2.73508700	-0.37417700	-2.85593500
H	-4.33879300	-1.11462700	-2.57593100
H	-4.03247300	0.58528200	-2.09906600
C	-2.56288900	-2.71766400	-0.80828100
H	-2.26878400	-3.19364500	0.13386700
H	-3.40648400	-3.26873900	-1.24466300
H	-1.71501600	-2.75296500	-1.50172400

C	-4.60328800	-1.10227300	0.42756200
H	-5.04758200	-0.10963400	0.56210200
H	-5.31152600	-1.74517300	-0.11122900
H	-4.41139400	-1.52978000	1.41861400
H	-2.22662300	1.38513200	-0.43502300

Complex PMe3-C4

Fe	-1.28826700	0.35105100	-0.30157600
Fe	1.40612400	0.23126500	-0.49549900
S	0.07752800	1.98645300	-1.22406000
S	0.24444900	0.32910300	1.54276900
O	3.86444100	1.81210100	-0.78302400
C	0.23931600	-0.93204100	-1.28927700
C	-2.45200200	1.48843100	0.41415600
C	2.87017900	1.23488300	-0.53797600
C	0.15830100	3.38798600	-0.01108500
C	0.23485600	2.05805500	2.21769200
C	0.87262200	3.12149500	1.31973800
H	-0.81140600	2.30872900	2.43804300
H	0.77859900	2.00179100	3.16876100
H	0.87538500	4.07060700	1.88251300
H	1.92248800	2.86144800	1.13341100
H	0.67811000	4.18929100	-0.55070200
H	-0.87659300	3.71205400	0.15972700
O	-3.27935200	2.25713500	0.73897800
C	-2.12407700	0.24574800	-1.83128100
O	-2.72281200	0.21759500	-2.83790400
O	0.03306300	-1.87579700	-1.98874100
P	-2.45775000	-1.39636900	0.49670300
P	2.61095400	-1.45664200	0.22259300
C	-2.49006600	-1.58219500	2.33587400
H	-3.17682600	-2.38792800	2.62692100
H	-1.48260400	-1.79801500	2.70594200
H	-2.82129800	-0.63998100	2.78740100
C	-4.26141600	-1.26702800	0.09876000
H	-4.40329900	-1.27166300	-0.98799700
H	-4.81186400	-2.11123600	0.53407900
H	-4.66759000	-0.33065700	0.49763600
C	-2.09542400	-3.11880100	-0.06962300
H	-1.11617500	-3.43788800	0.29886400
H	-2.86367900	-3.80507700	0.31033000
H	-2.07712500	-3.15515300	-1.16361100
C	3.76622600	-1.04597600	1.60693300
H	4.32848800	-1.93330400	1.92678600
H	4.47147000	-0.26794500	1.29364800
H	3.17819800	-0.66522800	2.45025700
C	3.70615000	-2.23431800	-1.04420000
H	4.28198900	-3.06812100	-0.62157200
H	3.08622700	-2.59820400	-1.87173800
H	4.39590900	-1.47845500	-1.43678000

C	1.72231800	-2.91250600	0.93542300
H	1.12906700	-3.40362600	0.15656200
H	2.43519400	-3.63733400	1.35027000
H	1.05683100	-2.56536500	1.73501100
H	1.93191100	0.06663500	-1.89386600

Complex PMe3-C5

Fe	1.49541100	0.37243000	0.24168300
Fe	-1.47827300	0.21627900	0.60168600
S	0.06095900	1.86145500	1.24621200
S	-0.20341700	0.23832200	-1.41143300
O	-3.63984000	2.18636100	0.51156100
C	-0.72505500	-1.05162800	1.58796300
C	2.79766200	1.43391500	-0.32034600
C	-2.73804000	1.43676600	0.43706800
C	-0.21730300	3.29418700	0.09664500
C	-0.55813300	1.93201400	-2.09678300
C	0.14595900	3.08673300	-1.37879700
H	-0.23570000	1.90117000	-3.14503100
H	-1.64904700	2.05300100	-2.07253900
H	1.23144700	2.96662300	-1.47921800
H	-0.11616600	4.01680000	-1.91174700
H	-1.27034100	3.58088400	0.20766700
H	0.39712800	4.10310600	0.51081400
O	3.72519200	2.12268100	-0.55213500
C	2.18494800	0.01752600	1.81018400
O	2.70219200	-0.18317500	2.84622600
O	-0.46143500	-1.89350800	2.36413400
P	2.48185900	-1.43821200	-0.57688600
P	-2.84940400	-1.25157300	-0.30335200
C	2.65797000	-1.49629900	-2.41738900
H	3.19570500	-2.40143000	-2.72937500
H	1.66650100	-1.47355300	-2.88134400
H	3.21415600	-0.61200000	-2.74985000
C	4.23247300	-1.66174000	-0.02262900
H	4.26600900	-1.78238200	1.06582500
H	4.67711700	-2.54635500	-0.49694000
H	4.82007300	-0.77622000	-0.29071000
C	1.75530700	-3.09488200	-0.18755100
H	0.75456700	-3.17023800	-0.62408100
H	2.38392200	-3.90089300	-0.58992500
H	1.66821000	-3.20990900	0.89856500
C	-3.68290600	-0.68187800	-1.85054200
H	-4.31772200	-1.47544400	-2.26600900
H	-4.29985500	0.20051600	-1.64645000
H	-2.91223600	-0.41312300	-2.58250600
C	-4.25476200	-1.79322900	0.76673600
H	-4.90025900	-2.51283000	0.24643100
H	-3.85822900	-2.25359000	1.67908100
H	-4.84718700	-0.91693900	1.05401000

C	-2.10991300	-2.84802400	-0.86222800
H	-1.64622800	-3.36658300	-0.01551400
H	-2.87635400	-3.49780200	-1.30526600
H	-1.34392500	-2.63192600	-1.61568300
H	-2.19278300	0.26335800	1.92701900

Complex PMe3-C6

Fe	-1.29511600	-0.30578400	0.42812900
Fe	1.43849100	-0.34503900	0.12754200
S	0.13863800	-1.99620500	1.01490000
S	-0.19114200	-0.32136300	-1.60669000
P	-2.63652600	1.26804400	-0.36849300
P	2.21381100	1.65965300	-0.46048200
O	-3.55230400	-2.12384000	0.78791500
O	2.32773900	0.26184000	2.82346800
O	-1.29021300	0.95881500	3.07171300
O	3.99806500	-1.67655600	0.02742100
C	-2.66789000	-1.37279900	0.57063000
C	1.91883500	0.03045500	1.74083500
C	-1.24741900	0.44735900	2.01159300
C	2.83204500	-1.19805500	-0.35878000
C	-0.05487000	-3.39484200	-0.19248200
C	-0.33237400	-2.02272700	-2.35211600
C	0.42888200	-3.12668600	-1.61846900
C	-3.98105900	1.81503000	0.77854800
C	4.02548100	1.87080900	-0.14972400
C	-1.98510700	2.89729200	-0.96747500
C	2.08247100	2.15307500	-2.23497500
C	-3.58524600	0.71455200	-1.85890900
C	1.51792300	3.09781700	0.46598600
H	0.42939700	3.09790700	0.37332900
H	-3.54064500	2.26121300	1.67735000
H	4.58939500	1.14916300	-0.75157700
H	-4.57709800	0.94715800	1.08368800
H	4.24239400	1.69098000	0.90996500
H	-2.78070800	3.47375500	-1.45918300
H	2.54429000	3.13591800	-2.39735600
H	-1.17416500	2.72909400	-1.68457600
H	1.03334100	2.17635800	-2.54424700
H	-1.60491200	3.48106700	-0.12179400
H	2.59545400	1.39977300	-2.84362800
H	-4.63596100	2.54912000	0.29137200
H	4.34653700	2.88781400	-0.40988900
H	-4.25977200	1.50453900	-2.21533100
H	1.92410900	4.04851900	0.09498900
H	-4.17023100	-0.17835800	-1.61120400
H	1.76435700	2.98806400	1.52848100
H	-2.87062600	0.45681000	-2.64889900
H	-1.40680300	-2.24472400	-2.39967400
H	0.04958200	-1.91970300	-3.37540500

H	0.31584100	-4.06027400	-2.19558100
H	1.49921100	-2.87963100	-1.60414600
H	0.51253500	-4.22411800	0.24802500
H	-1.12020700	-3.65999900	-0.17466900
H	4.17139800	-1.55315400	0.99365500

Complex PMe3-C7

Fe	-1.16139900	-0.33053200	0.41713900
Fe	1.38382600	-0.25847400	0.12015200
S	0.25993400	-2.02650400	0.98391300
S	-0.15795400	-0.42552900	-1.65685700
P	-2.53755600	1.20768600	-0.41084400
P	1.96247300	1.87076700	-0.29708400
O	-3.40660500	-2.08129100	1.03846300
O	2.16871200	-0.75695800	2.84162700
O	-0.92465800	1.09285400	2.96004600
O	3.82002600	-1.45372500	-0.95927100
C	-2.53588100	-1.35370400	0.71597700
C	2.03911100	-0.06538000	1.74586000
C	-0.97398500	0.50889700	1.93819600
C	2.83185500	-0.96104300	-0.55373200
C	0.06392500	-3.46810900	-0.16140000
C	-0.27744800	-2.14791200	-2.34662600
C	0.51791500	-3.22245500	-1.60181200
C	-3.58828500	2.09426500	0.82632100
C	3.75220600	2.20264900	0.03500200
C	-1.94707000	2.59804500	-1.48598700
C	1.79711300	2.53943300	-2.01526000
C	-3.79326700	0.47300500	-1.55703700
C	1.15640100	3.13229200	0.78544500
H	0.06930900	3.06065500	0.69208500
H	-2.95227900	2.69091400	1.48998800
H	4.37342800	1.61236200	-0.64852300
H	-4.12690100	1.36236300	1.43947200
H	3.98173100	1.90143100	1.06341100
H	-2.80491300	3.19482300	-1.82510400
H	2.10764600	3.59184300	-2.05834000
H	-1.44141900	2.17463100	-2.36061900
H	0.76812400	2.44451700	-2.37110400
H	-1.25418100	3.25374700	-0.95081500
H	2.43803900	1.94763700	-2.67960100
H	-4.31238500	2.75144200	0.32751700
H	3.98179300	3.26834500	-0.09626900
H	-4.44569300	1.25092700	-1.97556700
H	1.49219600	4.14986600	0.54351900
H	-4.40348000	-0.26633100	-1.02701300
H	1.41426500	2.89728700	1.82469700
H	-3.26164800	-0.03256000	-2.37172600
H	-1.34712000	-2.39486000	-2.36488200
H	0.07817200	-2.07086200	-3.38158700

H	0.40608000	-4.17104100	-2.15360300
H	1.58625400	-2.97240500	-1.61551800
H	0.65441900	-4.27319900	0.29235100
H	-0.99561000	-3.75065300	-0.11846600
H	1.69029700	-1.63794100	2.74557500

Complex PMe3-C8

Fe	1.20907500	0.21851500	0.42930000
Fe	-1.36149700	0.38724500	0.20729800
S	-0.06175500	1.96592400	1.20006400
S	0.15551800	0.56825500	-1.59814800
P	2.49165900	-1.30365500	-0.54801700
P	-2.15452300	-1.58457700	-0.45804600
O	3.56409900	1.71799900	1.26534300
O	-1.94410000	-0.41299600	2.97741800
O	0.56341300	-1.31468700	2.81729600
O	-3.61345100	1.89412100	-0.90085300
C	2.64713400	1.10501100	0.84679000
C	-2.12769300	0.05054500	1.78342500
C	0.82052700	-0.72396100	1.80445200
C	-2.70559700	1.28775300	-0.46813500
C	0.24745900	3.46260200	0.14916800
C	0.40717400	2.32210800	-2.15728200
C	-0.25046700	3.40180400	-1.29560200
C	3.61940900	-2.21842000	0.59803500
C	-3.98213600	-1.75339000	-0.22174900
C	1.79262600	-2.67642200	-1.57680200
C	-1.98767800	-2.04956300	-2.24092000
C	3.66728600	-0.56344200	-1.77149600
C	-1.53937900	-3.07588200	0.44778200
H	-0.44665800	-3.10587700	0.41525500
H	3.02360000	-2.80651500	1.30580400
H	-4.50504600	-1.02086600	-0.84733600
H	4.21724100	-1.49983700	1.17047400
H	-4.23252500	-1.54994300	0.82571600
H	2.60551800	-3.25288300	-2.03880300
H	-2.46193600	-3.02110500	-2.43376400
H	1.17063200	-2.24528600	-2.36827700
H	-0.93515200	-2.08978200	-2.53184100
H	1.18519700	-3.35324300	-0.96758300
H	-2.47502200	-1.27881200	-2.84902500
H	4.29045500	-2.88687900	0.04288700
H	-4.31855600	-2.76326100	-0.49085400
H	4.29778100	-1.33592700	-2.23190400
H	-1.95290800	-3.99957900	0.02094600
H	4.30471600	0.17782700	-1.27672900
H	-1.84078900	-3.00451100	1.49913900
H	3.08238400	-0.05746900	-2.54838300
H	1.49406000	2.46842200	-2.20414300
H	0.00766900	2.36361100	-3.17821300

H	-0.03978300	4.37828100	-1.76390000
H	-1.34036300	3.27834600	-1.31062100
H	-0.25424600	4.27849800	0.68396500
H	1.32949700	3.64285900	0.18914200
H	-1.01170900	-0.77718500	3.13714500

Complex PMe3-C9

Fe	1.36659500	0.35453100	0.11592000
Fe	-1.16462300	0.23807700	0.42727000
S	0.12409200	2.05330600	0.95217300
S	-0.17046000	0.36887400	-1.66940300
P	2.17455000	-1.70747300	-0.30656300
P	-2.44315900	-1.37651600	-0.37890100
O	3.49994800	1.90385700	-1.12970200
O	-0.61305200	-1.25111600	2.87547500
O	2.22568100	1.03069700	2.77486300
O	-3.53332200	1.68330300	1.31437300
C	2.65767600	1.24069300	-0.64577700
C	-0.76849400	-0.63562300	1.88147500
C	2.11406100	0.28494700	1.71319200
C	-2.60992200	1.08735300	0.88524600
C	-0.11253600	3.45356400	-0.23664300
C	-0.29075800	2.07717200	-2.39439300
C	-0.93899400	3.11788600	-1.47993300
C	3.99755600	-1.81618500	-0.01102600
C	-3.72377500	-0.73919400	-1.55493000
C	1.54691200	-3.05798700	0.78690700
C	-1.73726800	-2.74829800	-1.40180500
C	2.04650900	-2.40248200	-2.01693100
C	-3.45220700	-2.29253100	0.87100000
H	-2.78796300	-2.81713400	1.56706000
H	4.20611300	-1.47875400	1.01074300
H	-3.21049400	-0.24991000	-2.39096100
H	4.52642000	-1.15778800	-0.71012300
H	-4.35882400	0.00035400	-1.05475300
H	2.01254800	-4.02324100	0.54616600
H	-2.54336200	-3.39612100	-1.77212000
H	0.45950900	-3.13398400	0.69497600
H	-1.03820500	-3.35557700	-0.81842800
H	1.77306500	-2.78919300	1.82528300
H	-1.20969100	-2.31062700	-2.25639400
H	4.35924900	-2.84518700	-0.13897800
H	-4.35070100	-1.55499600	-1.93945400
H	2.51229600	-3.39527200	-2.07243200
H	-4.11687000	-3.01894800	0.38549800
H	2.55874800	-1.72387300	-2.70918300
H	-4.05570700	-1.58113500	1.44677800
H	1.00091600	-2.47216900	-2.32867700
H	0.72640800	2.37766100	-2.67695700
H	-0.87992200	1.95943000	-3.31210000

H	-1.07668100	4.04912200	-2.05403600
H	-1.93742900	2.77067800	-1.17793500
H	-0.60461500	4.23887800	0.34969800
H	0.89254300	3.80049300	-0.50992700
H	1.65579100	1.85460700	2.67258400

Complex PMe3-C10

Fe	1.35723600	0.18997700	0.50724300
Fe	-1.53853000	0.39505600	0.16357800
S	-0.10451200	1.89920700	1.14019900
S	0.10715100	0.30431200	-1.52979000
P	2.72275600	-1.20630800	-0.52774600
P	-2.33623500	-1.58690700	-0.41548600
O	3.58475100	1.98053300	1.02519600
O	-2.69881000	-0.03752500	2.80835100
O	1.08122000	-1.29901000	3.03222000
O	-3.84637900	1.94738000	-0.81624900
C	2.67417200	1.29164700	0.71309800
C	-2.21250900	0.12073700	1.74715300
C	1.08875500	-0.75038800	1.84218500
C	-2.88296400	1.34654500	-0.49997600
C	0.23487500	3.32890800	0.00647200
C	0.47073900	2.00335700	-2.19794800
C	-0.20014200	3.15764600	-1.45119600
C	4.17747500	-1.77113800	0.46442100
C	-4.13887900	-1.81503900	-0.06411600
C	2.01035000	-2.80034400	-1.13081200
C	-2.25314900	-2.07866900	-2.19778000
C	3.51404200	-0.53567400	-2.05710300
C	-1.59173500	-3.03919000	0.45737600
H	-0.50576800	-3.01808500	0.32582200
H	3.82534600	-2.31262700	1.35035200
H	-4.71672100	-1.07420100	-0.62876400
H	4.75190800	-0.89847100	0.79599100
H	-4.33131300	-1.66184300	1.00373800
H	2.75128200	-3.36522700	-1.71221100
H	-2.71279700	-3.06444900	-2.34952300
H	1.14246200	-2.58614500	-1.76358300
H	-1.21285700	-2.09704600	-2.53720900
H	1.68959200	-3.40429400	-0.27451000
H	-2.78840800	-1.33207900	-2.79591600
H	4.82978100	-2.42959900	-0.12412000
H	-4.46750800	-2.82333600	-0.34888200
H	4.19215200	-1.27353400	-2.50621500
H	-1.99756300	-3.98972600	0.08484500
H	4.07777900	0.37190400	-1.81267000
H	-1.79888100	-2.95145200	1.53042900
H	2.72679100	-0.28053600	-2.77517400
H	1.56348000	2.11079200	-2.18316800
H	0.13279300	1.98793600	-3.24173600

H	0.05132800	4.09065500	-1.98432100
H	-1.29063200	3.04913100	-1.50642200
H	-0.30301400	4.17205800	0.45792500
H	1.31115300	3.53263000	0.07628100
H	1.64079900	-0.80278400	3.67286900

Complex PMe3-C11

Fe	-1.40375700	-0.28761600	0.41477900
Fe	1.48203900	-0.40586900	0.17938000
S	0.05996000	-1.99371900	1.01169200
S	-0.08482100	-0.21615700	-1.58024100
P	-2.66664500	1.27721200	-0.50411100
P	2.35508600	1.57339200	-0.29455500
O	-3.62091200	-1.82469200	1.54147900
O	2.60210200	-0.13352400	2.86399400
O	-0.82467300	1.35401700	2.74381100
O	3.79055000	-1.94174200	-0.80694800
C	-2.70851200	-1.22719200	0.81004800
C	2.11528200	-0.21991600	1.79604700
C	-0.95620000	0.71288600	1.75894600
C	2.83010300	-1.33701400	-0.48889200
C	-0.25240000	-3.34435100	-0.22101900
C	-0.47135700	-1.87506100	-2.33550800
C	0.19843600	-3.07287300	-1.65864400
C	-4.05313500	1.90209900	0.54831000
C	4.15784400	1.71131600	0.10285800
C	-1.87923500	2.85098000	-1.06885800
C	2.33518300	2.13275500	-2.05889800
C	-3.53276800	0.71509900	-2.03578400
C	1.66165300	3.02715900	0.61592500
H	0.58855200	3.10192300	0.41836400
H	-3.64751000	2.31904300	1.47762300
H	4.71782900	0.96959600	-0.47835500
H	-4.72034900	1.07014700	0.80242500
H	4.32012000	1.50955300	1.16769300
H	-2.59526300	3.45926000	-1.63759300
H	2.82421800	3.11091200	-2.15835600
H	-1.02156100	2.61331200	-1.70689100
H	1.30714900	2.19105100	-2.43064300
H	-1.53736000	3.42632400	-0.20114000
H	2.87002900	1.39499200	-2.66832700
H	-4.63076500	2.67693500	0.02736500
H	4.53404800	2.71550100	-0.13399600
H	-4.17627100	1.50955100	-2.43683600
H	2.15824600	3.96112200	0.31874100
H	-4.13756400	-0.16927900	-1.80714200
H	1.79289400	2.87132000	1.69284600
H	-2.77978500	0.44283400	-2.78387400
H	-1.56452400	-1.97910200	-2.31761600
H	-0.14005000	-1.80354200	-3.37894500

H	-0.04221600	-3.96997800	-2.25477900
H	1.28885800	-2.95644700	-1.69441100
H	0.28027700	-4.21708500	0.17728700
H	-1.33142300	-3.54446400	-0.18087900
H	-3.56763700	-1.56174000	2.49021300

Complex PMe3-C12

Fe	1.39211600	0.41471000	0.22472300
Fe	-1.13874700	0.12870800	0.44875500
S	0.02596500	2.00844000	1.12617000
S	-0.02269200	0.33175400	-1.60276900
P	2.34338200	-1.50232900	-0.46765100
P	-2.75996900	-1.15950100	-0.39890800
O	3.40712900	2.21766700	-0.85803300
O	-0.08546500	-2.03744500	2.07076200
O	2.70539700	0.19317400	2.82740700
O	-3.32295000	1.32497400	1.43555100
C	2.60115300	1.47374900	-0.43778100
C	-0.36640500	-1.10578500	1.38419000
C	2.15371200	0.26397900	1.79807300
C	-2.33213400	0.67423100	1.80380300
C	-0.30073300	3.38225400	-0.07462400
C	-0.28058200	2.04620200	-2.27306300
C	-1.05324900	2.99201700	-1.34948000
C	3.42591400	-2.36114900	0.75638400
C	-3.97361000	-0.14095900	-1.35372200
C	1.24273400	-2.87473200	-1.02818800
C	-2.35658700	-2.49743000	-1.61019900
C	3.45802700	-1.29016400	-1.92647000
C	-3.83065700	-2.05511800	0.81254900
H	-3.21224000	-2.72689700	1.41925100
H	2.82937800	-2.63104000	1.63516600
H	-3.46941100	0.28401300	-2.22981500
H	4.23672800	-1.69715100	1.07617000
H	-4.32059600	0.67473300	-0.70992700
H	1.84442100	-3.71935900	-1.39022200
H	-3.28129000	-2.94424500	-1.99982800
H	0.60179400	-2.51489900	-1.83916000
H	-1.76453200	-3.28124000	-1.12557400
H	0.61996900	-3.20444400	-0.19049100
H	-1.78002400	-2.07717800	-2.44121500
H	3.85664900	-3.26936900	0.31548500
H	-4.82693400	-0.74868100	-1.68469800
H	3.89721100	-2.25043300	-2.22746500
H	-4.60140900	-2.64081300	0.29452800
H	4.26143900	-0.58729100	-1.67870300
H	-4.31297100	-1.32819900	1.47438800
H	2.87991100	-0.87660100	-2.76110600
H	0.70913200	2.45198900	-2.51770500
H	-0.82874000	1.89633100	-3.21120600

H	-1.26537800	3.91678900	-1.91217000
H	-2.02504600	2.54876000	-1.08330800
H	-0.88847400	4.10615800	0.50268000
H	0.66894300	3.84030300	-0.31107100
H	-2.17807500	0.50470300	2.89522500

Complex PMe3-C14

Fe	1.14777000	0.13447600	0.47255200
Fe	-1.40859800	0.38931400	0.18323100
S	-0.08939900	1.92635600	1.23282000
S	0.08279400	0.41162700	-1.56335600
P	2.69852500	-1.17252000	-0.44283100
P	-2.24886500	-1.55397100	-0.51618000
O	3.22925600	1.79900400	0.71620800
O	-2.57750200	-0.17156800	2.80253600
O	0.63391700	-1.82693700	2.56345200
O	-3.66609000	1.99998500	-0.72618700
C	2.51267200	1.05068500	1.39972900
C	-2.08735400	0.04066200	1.75887800
C	0.78054500	-1.01197300	1.71799700
C	-2.74570400	1.34958900	-0.39072200
C	0.37648300	3.35703400	0.14991100
C	0.52612600	2.14336300	-2.11071700
C	-0.10280100	3.27702700	-1.30064200
C	3.94956700	-1.88793600	0.71430600
C	-4.04739800	-1.74942400	-0.12713500
C	2.14665600	-2.66342500	-1.39136800
C	-2.22463000	-1.90104600	-2.33246300
C	3.74357500	-0.29481200	-1.68941100
C	-1.53087100	-3.09198900	0.20942300
H	-0.46967300	-3.14874300	-0.05040500
H	3.44239700	-2.48038300	1.48440300
H	-4.61884600	-0.94790500	-0.60933600
H	4.49614700	-1.07400400	1.20379800
H	-4.20310700	-1.68264500	0.95544700
H	3.00262100	-3.14815800	-1.88036900
H	-2.74316200	-2.84530200	-2.54444500
H	1.42114400	-2.36046800	-2.15451900
H	-1.19293500	-1.95794200	-2.69302500
H	1.67568500	-3.38416200	-0.71329200
H	-2.72485700	-1.08284800	-2.86309600
H	4.66301300	-2.52542700	0.17619900
H	-4.41592800	-2.72015300	-0.48341800
H	4.57102600	-0.93282700	-2.02777700
H	-2.05199800	-3.98458800	-0.16248600
H	4.13854400	0.62154800	-1.23746900
H	-1.60627800	-3.05055700	1.30142400
H	3.11768400	-0.02652500	-2.54844300
H	1.62136600	2.20325900	-2.07580900
H	0.20239500	2.19615100	-3.15706200

H	0.17198300	4.22432000	-1.79468900
H	-1.19738200	3.20657700	-1.33833200
H	-0.05154500	4.23615800	0.64603400
H	1.47227000	3.42539100	0.19560800
H	2.68390300	0.99992800	2.49963500

Complex PMe3-C15

Fe	1.27542600	0.33104800	0.42295400
Fe	-1.47253600	0.31561200	0.18296100
S	-0.21315900	1.88550700	1.24074800
S	0.01638200	0.52624000	-1.56465200
P	2.63633800	-1.25475400	-0.34981800
P	-2.01934500	-1.74833200	-0.46012600
O	3.37639900	2.30654100	0.27584200
O	-2.65694400	-0.27428100	2.79021600
O	0.70723700	-1.17836100	2.35586100
O	-3.87303400	1.66246800	-0.80807400
C	2.52943300	1.48108000	0.33356800
C	-2.15857200	-0.05916600	1.75176800
C	1.54182300	-0.27905300	2.13376000
C	-2.89571900	1.12935300	-0.42819600
C	-0.04596400	3.43057000	0.22449300
C	0.21707300	2.30379200	-2.08709700
C	-0.51375500	3.33829000	-1.22978600
C	4.32719000	-1.23056300	0.40543900
C	-3.84317800	-2.01646500	-0.63891200
C	2.16274400	-3.02504700	-0.07792200
C	-1.40612600	-2.37473100	-2.08984300
C	3.02363100	-1.20872200	-2.15765800
C	-1.56502200	-3.09756500	0.71800000
H	-0.50091600	-3.03418300	0.95924900
H	4.24742000	-1.40154100	1.48566200
H	-4.24154400	-1.35015500	-1.41239300
H	4.79395000	-0.25227900	0.24505700
H	-4.34346300	-1.78162000	0.30790300
H	2.99229300	-3.69321200	-0.34627700
H	-1.74228700	-3.40784700	-2.25007600
H	1.28974900	-3.28382300	-0.68603800
H	-0.31306100	-2.33278600	-2.13008500
H	1.90737200	-3.16626500	0.97803000
H	-1.79225600	-1.73488300	-2.89094300
H	4.96327400	-2.01060000	-0.03309800
H	-4.05962400	-3.05856800	-0.90952300
H	3.71772000	-2.01297000	-2.43518200
H	-1.80650700	-4.08383700	0.29912900
H	3.47716900	-0.24106000	-2.40292500
H	-2.12074000	-2.95653600	1.65225900
H	2.09551000	-1.30173700	-2.73222400
H	1.29689400	2.49589100	-2.10063300
H	-0.15628100	2.34179600	-3.11782600

H	-0.35079600	4.32678000	-1.69240200
H	-1.59425600	3.15193600	-1.26474200
H	-0.64272600	4.17659100	0.76305300
H	1.00796700	3.72892000	0.28441200
H	2.22293300	0.04560700	2.95102300

Complex PMe3-C16

Fe	1.38683100	0.24691400	0.44824900
Fe	-1.46825400	0.38783400	0.19491900
S	-0.02945400	1.90845900	1.13789800
S	0.10666700	0.31000100	-1.54247800
P	2.63847200	-1.39269300	-0.35920200
P	-2.40844600	-1.54770500	-0.38438800
O	3.72080400	1.89409100	0.83119500
O	-2.81386400	0.24062400	2.78746200
O	0.39589400	-1.29916800	2.16578500
O	-3.44621800	2.19776100	-0.98783800
C	2.76898100	1.20856200	0.66753200
C	-2.25701300	0.26630400	1.75567100
C	1.37128100	-0.52244000	2.10182100
C	-2.65540500	1.45064700	-0.53818700
C	0.12135500	3.35235900	-0.02228800
C	0.14732200	2.03552800	-2.24696000
C	0.81505400	3.08752100	-1.36153600
C	4.08789800	-1.89700800	0.67256700
C	-4.24828400	-1.56217200	-0.18269000
C	1.80990200	-3.01553300	-0.67491700
C	-2.22568200	-2.11959700	-2.13377600
C	3.41506100	-1.02018100	-1.99447900
C	-1.91642700	-3.02835500	0.60544100
H	-0.83519200	-3.17133400	0.53787400
H	3.73395000	-2.27744000	1.63812300
H	-4.69697200	-0.78852000	-0.81634000
H	4.73048200	-1.02814400	0.85621700
H	-4.50530600	-1.34602300	0.86071100
H	2.51385300	-3.74118800	-1.10444100
H	-2.74801200	-3.07376700	-2.28305900
H	0.97702500	-2.86856700	-1.37157800
H	-1.16715300	-2.23123300	-2.38912600
H	1.41874500	-3.40904400	0.26985800
H	-2.65174800	-1.36275900	-2.80245800
H	4.67552400	-2.67918200	0.17435200
H	-4.66248400	-2.54092400	-0.45876100
H	4.03621900	-1.85778200	-2.33845700
H	-2.44011300	-3.92883800	0.25668000
H	4.03741900	-0.12171500	-1.90853500
H	-2.15638500	-2.84715300	1.65907100
H	2.62009600	-0.82524300	-2.72290700
H	0.69499000	1.93848800	-3.19230200
H	-0.89075700	2.30635400	-2.47521000

H	1.86375700	2.81102000	-1.18952900
H	0.82308700	4.03871400	-1.92106500
H	-0.90163800	3.72003500	-0.17185400
H	0.67921900	4.10816000	0.54360500
H	2.02538300	-0.39702200	2.99186500

Complex PMe3-D1

Fe	-1.28822700	0.10134800	0.57040400
Fe	1.28869600	0.07269400	-0.57438200
S	-0.60945700	1.12477800	-1.50359800
S	0.65117200	1.16962300	1.44514000
P	-2.69755000	-1.21407100	-0.63003300
P	2.72591000	-1.17627000	0.64770100
O	-1.44850100	-1.61803500	2.92005000
O	-3.46787200	1.85843300	1.57224700
O	1.47696500	-1.67398900	-2.89765700
O	3.33513800	1.99655700	-1.49135000
C	-1.36775800	-0.95572500	1.97926500
C	-2.60626400	1.23052600	1.13712900
C	1.39729400	-0.98968300	-1.97514700
C	2.53234300	1.26307300	-1.11647100
C	-0.37291500	2.94505000	-1.30375600
C	-0.41248800	3.50159800	0.11794300
C	0.64744100	2.98164400	1.08769400
C	-3.61976000	-2.51447300	0.29716300
C	-4.05851900	-0.30546000	-1.47708300
C	-1.96142500	-2.18423700	-2.01151700
C	1.99886600	-2.10320200	2.06037100
C	4.08552700	-0.23258300	1.45628900
C	3.64901800	-2.49844100	-0.24665700
H	4.22475400	-2.06303600	-1.06595500
H	0.07716900	-0.87449700	-0.02782500
H	-1.16572300	3.40872400	-1.89510700
H	0.57824300	3.18842700	-1.78495300
H	-1.40540300	3.34858100	0.54424100
H	-0.27868600	4.59011400	0.04503900
H	1.64891200	3.23124500	0.72558300
H	0.51786500	3.46878700	2.05654700
H	-4.30353300	-3.05544100	-0.36217900
H	-2.91511600	-3.22205700	0.73768600
H	-4.19349300	-2.05945900	1.10708900
H	-4.67913900	0.20054300	-0.73524700
H	-3.62685100	0.44724800	-2.13826600
H	-4.68426800	-0.98575700	-2.06117800
H	-2.72887600	-2.75542000	-2.54060000
H	-1.47337500	-1.50712100	-2.71375800
H	-1.21087300	-2.87096900	-1.61678500
H	2.77224200	-2.64817400	2.60784300
H	1.50524300	-1.40570300	2.73813400
H	1.25426000	-2.80935800	1.68982800

H	4.70344700	0.24835400	0.69551100
H	3.65202900	0.54134300	2.09123800
H	4.71519400	-0.88852800	2.06349300
H	4.33123400	-3.02341500	0.42698300
H	2.94570200	-3.21708400	-0.67128500

Complex PMe3-D2

Fe	1.48236400	0.33671100	0.51912700
Fe	-1.43159200	-0.21844800	-0.54793100
S	-0.58874800	1.28834400	1.12078400
S	0.54951300	0.50576000	-1.65389000
O	-0.51929900	-2.74345100	0.65796600
C	2.59688200	1.76738800	0.63566000
C	-0.75960100	-1.70531500	0.22162000
C	-0.55887800	2.98386800	0.37061300
C	0.38161600	2.32541500	-1.96870000
C	-0.66204200	3.08220300	-1.14973400
H	1.37879800	2.74085000	-1.80560500
H	0.14797200	2.42659600	-3.03041400
H	-0.57816100	4.14527000	-1.41573700
H	-1.65724500	2.75984400	-1.46189300
H	-1.39120600	3.52931800	0.82148900
H	0.36386200	3.44314900	0.73069900
O	3.26081900	2.70472900	0.73118700
C	1.68629600	-0.28498400	2.16816100
O	1.82216800	-0.66744900	3.24626700
C	-2.01056700	-0.99198100	-2.01452800
O	-2.40134700	-1.42709600	-3.00230100
H	-2.20901800	0.91271000	-1.20568400
P	-3.35979600	-0.36833800	0.59190400
P	3.01401000	-1.12179500	-0.32090300
C	-4.22557500	1.23280600	0.85226700
H	-5.14387600	1.09158100	1.42798200
H	-3.56417500	1.91265000	1.39017900
H	-4.46720300	1.67383400	-0.11586100
C	-3.23924700	-1.02996000	2.30577300
H	-4.21271600	-1.01902400	2.80271800
H	-2.86292000	-2.05417500	2.28381600
H	-2.53824600	-0.41644300	2.87444500
C	-4.67967400	-1.41456800	-0.15560500
H	-5.58011400	-1.41361000	0.46357900
H	-4.92943400	-1.03771800	-1.14905700
H	-4.32154900	-2.44043800	-0.26169600
C	4.23976100	-1.82008600	0.86524300
H	3.72540400	-2.41617200	1.62083300
H	4.97063400	-2.44852900	0.35044700
H	4.76241300	-1.00674200	1.37245800
C	2.39285700	-2.63765800	-1.16955400
H	1.71726600	-2.35106100	-1.97745500
H	3.22167100	-3.21617900	-1.58644300

H	1.84311900	-3.25907000	-0.46136600
C	4.10075300	-0.38186000	-1.61189700
H	4.80285300	-1.12057600	-2.00747400
H	3.48008500	-0.00059500	-2.42365300
H	4.66203200	0.45052600	-1.18358100

Complex PMe3-D3

Fe	-1.46665900	0.33886600	-0.50756800
Fe	1.44444900	-0.19338100	0.55399200
S	0.59199200	1.33733600	-1.07727200
S	-0.53868900	0.46841300	1.68946000
O	0.59400400	-2.74845300	-0.63357200
C	-2.68868700	1.66613300	-0.74972700
C	0.82217900	-1.70485700	-0.20416000
C	0.71022800	2.97626300	-0.22119900
C	-0.21027900	2.24079000	2.10366700
C	-0.25587600	3.22257100	0.93576900
H	-0.97065500	2.51841000	2.83725500
H	0.76304800	2.26031900	2.59625800
H	-1.27582600	3.28011600	0.55196700
H	-0.02926400	4.21988100	1.33838500
H	1.74373300	3.06610200	0.11796200
H	0.53664100	3.72630000	-0.99619800
O	-3.48209900	2.47536200	-0.95933200
C	-1.56588100	-0.36002600	-2.13520400
O	-1.64050800	-0.79036700	-3.20124800
C	2.05399400	-0.93610100	2.02356300
O	2.45906700	-1.34672900	3.01616600
H	2.15123400	0.99184700	1.20768100
P	3.37008100	-0.30598500	-0.59900900
P	-2.97797400	-1.14492000	0.31570500
C	4.24816700	1.29510000	-0.81232500
H	5.16534900	1.16197400	-1.39170000
H	3.59453000	1.99600600	-1.33251200
H	4.49342600	1.70679200	0.16768900
C	3.23686900	-0.91111400	-2.33250800
H	4.20747400	-0.88631000	-2.83463100
H	2.85890400	-1.93495900	-2.34120600
H	2.53378800	-0.27808000	-2.87641100
C	4.68634800	-1.38559500	0.10701200
H	5.58220600	-1.37441400	-0.51868600
H	4.94703400	-1.04080200	1.10927400
H	4.31959500	-2.41096500	0.18570200
C	-4.19021600	-1.83947600	-0.88595300
H	-3.66646800	-2.42061000	-1.64664700
H	-4.91675700	-2.48214700	-0.38265900
H	-4.71990100	-1.02523400	-1.38441100
C	-2.34019600	-2.66291200	1.14634100
H	-1.66961900	-2.37772600	1.95884100
H	-3.16357800	-3.25539900	1.55407000

H	-1.78195500	-3.26915100	0.43183700
C	-4.07706800	-0.42952900	1.60973900
H	-4.77670700	-1.17847100	1.98992900
H	-3.46381700	-0.05568900	2.43034800
H	-4.64058700	0.40520700	1.18917800

Complex PMe3-D4

Fe	1.45110200	0.07885300	0.57215200
Fe	-1.42535900	-0.05414900	-0.68004400
S	-0.63279300	0.71257100	1.48120400
S	0.55302200	1.08797300	-1.39049800
O	-3.18837400	1.78035000	-2.11299900
C	2.51594300	1.33409400	1.33761100
C	-2.47693400	1.15023100	-1.46299900
C	-0.59690600	2.56247400	1.47209500
C	0.37299100	2.87801900	-0.93308700
C	-0.67882100	3.25091300	0.11241400
H	1.36517700	3.19351600	-0.60235900
H	0.15132300	3.40425500	-1.86387600
H	-0.58460200	4.33198000	0.28699200
H	-1.67673700	3.10614300	-0.30358800
H	-1.43939700	2.88637400	2.08763000
H	0.31668800	2.84588600	1.99822400
O	3.14130400	2.14247100	1.87101900
C	1.60204000	-1.19588500	1.79837200
O	1.70453700	-2.00764800	2.60985900
C	-0.67307000	-1.67172600	-0.72886200
O	-0.36934300	-2.76629300	-0.92486100
H	-1.73671800	-0.58656800	-2.04989000
P	-3.22655800	-0.95532000	0.32365500
P	3.08700000	-0.82849500	-0.71802800
C	-4.31170900	0.26066600	1.17624000
H	-4.69118300	0.99379100	0.46250700
H	-5.15562900	-0.23947200	1.65774200
H	-3.72250000	0.78169100	1.93197300
C	-4.38721500	-1.87163100	-0.77194800
H	-3.85519700	-2.69279400	-1.25585100
H	-5.23367000	-2.27343300	-0.20938500
H	-4.75844700	-1.20137900	-1.54954400
C	-2.86773700	-2.15519900	1.67001500
H	-3.79223700	-2.50099800	2.13932400
H	-2.32521200	-3.01612600	1.27597600
H	-2.24456300	-1.66551100	2.41990300
C	2.59620500	-1.86851700	-2.16061300
H	1.93566300	-1.29283900	-2.81128100
H	3.47631000	-2.18054700	-2.72932100
H	2.05686500	-2.75191700	-1.81752800
C	4.32062100	-1.90238500	0.13287700
H	3.82708400	-2.78710600	0.53836000
H	5.10762500	-2.21682500	-0.55699700

H	4.77139200	-1.35485200	0.96296300
C	4.16385100	0.43547800	-1.51825100
H	4.92362500	-0.03687400	-2.14641600
H	3.54712300	1.09378000	-2.13151200
H	4.65706100	1.03581900	-0.75187300

Complex PMe3-D5

Fe	1.42346100	0.07081300	0.56577300
Fe	-1.44989400	-0.01500900	-0.70408300
S	-0.64845900	0.76671100	1.43527000
S	0.55218600	1.06814400	-1.44395300
O	-3.17945100	2.03711700	-1.85661000
C	2.55453700	1.14067500	1.50107900
C	-2.47428800	1.29975700	-1.32412200
C	-0.76483600	2.61055700	1.27993900
C	0.27782000	2.86569200	-1.09810700
C	0.25751800	3.28838700	0.36931500
H	1.09520500	3.38282800	-1.60550800
H	-0.64784100	3.15043300	-1.60140200
H	1.25598700	3.16629100	0.79250600
H	0.04983200	4.36744800	0.39389100
H	-1.78271400	2.83315100	0.95116100
H	-0.65188800	3.00050600	2.29362400
O	3.24264400	1.77176000	2.17730500
C	1.41498500	-1.31896300	1.67331600
O	1.41996600	-2.20547800	2.41007000
C	-0.75529200	-1.64706900	-0.87864200
O	-0.47063400	-2.73198900	-1.14263000
H	-1.85122600	-0.45328100	-2.08691200
P	-3.22946800	-0.92953200	0.32804500
P	3.08016400	-0.82423200	-0.69327100
C	-4.30993400	0.26623100	1.21414900
H	-4.73095300	0.98806300	0.51231200
H	-5.12576900	-0.25051200	1.72546600
H	-3.70707200	0.80230000	1.94848300
C	-4.40474700	-1.85077400	-0.74779500
H	-3.87490800	-2.66180900	-1.25080500
H	-5.23374600	-2.26657100	-0.16951300
H	-4.80039500	-1.17812600	-1.51118100
C	-2.82812800	-2.13332400	1.65907700
H	-3.73868600	-2.49781200	2.14145200
H	-2.27730900	-2.98248400	1.25106600
H	-2.20125200	-1.63828300	2.40228600
C	2.61638200	-1.87881500	-2.13259600
H	1.97096200	-1.30872300	-2.80296900
H	3.50755700	-2.19933700	-2.67875500
H	2.06708600	-2.75687500	-1.79126400
C	4.30059500	-1.88190600	0.19459000
H	3.80216300	-2.76291800	0.60204700
H	5.10211700	-2.20330000	-0.47510800

H	4.73355400	-1.32124600	1.02531700
C	4.16232300	0.44096700	-1.48320900
H	4.94790800	-0.03202200	-2.07826200
H	3.55482900	1.07714300	-2.12802200
H	4.62191800	1.06324200	-0.71356700

Complex PMe3-D6

Fe	-1.42921400	-0.04879600	0.58359000
Fe	1.45876100	0.18348500	-0.57664200
S	-0.54624300	1.07191500	-1.41278200
S	0.54863500	0.84453300	1.51799600
P	-3.14501000	-0.70491400	-0.69262100
P	3.03393300	-1.00201500	0.55523200
O	-0.69815100	-2.81914900	0.96634500
O	-2.97569300	0.34963100	3.01561100
O	1.64565200	-1.56207400	-2.91987300
O	3.20989500	2.38656900	-1.50628000
C	-0.89261800	-1.69839000	0.73325400
C	-2.37856300	0.39093100	1.86162000
C	1.56946200	-0.88602100	-1.98886800
C	2.57442600	1.51216900	-1.10414800
C	-0.52949900	2.87312500	-0.97602400
C	-0.72299500	3.21801700	0.49761200
C	0.33571300	2.68437500	1.46022700
C	-4.36807400	-1.82633900	0.10760600
C	-4.19125300	0.67576500	-1.30870600
C	-2.71239200	-1.61887700	-2.22933800
C	2.48879400	-2.24437500	1.80334800
C	4.15713900	0.06519800	1.55415900
C	4.22732000	-1.97622200	-0.45896700
H	4.71533900	-1.31998300	-1.18226700
H	-1.33077200	3.32800400	-1.56300700
H	0.41646100	3.27105900	-1.34970100
H	-1.70671200	2.87342700	0.82531100
H	-0.72427300	4.31350500	0.58705300
H	1.32115300	3.09228200	1.22055200
H	0.08458100	2.98601400	2.47933400
H	-5.16020800	-2.10922300	-0.59009000
H	-3.86273300	-2.72940400	0.45557300
H	-4.81528100	-1.32616300	0.96871700
H	-4.58474500	1.23748900	-0.46007700
H	-3.57355600	1.34249800	-1.91132800
H	-5.02010500	0.30263900	-1.91622800
H	-3.60896300	-1.86158500	-2.80575900
H	-2.04700700	-1.00252000	-2.83479500
H	-2.19188800	-2.54316000	-1.97227100
H	3.35090500	-2.69109200	2.30564300
H	1.85540400	-1.75533600	2.54525100
H	1.90467900	-3.02808200	1.31995400
H	4.68550200	0.75494300	0.89356200

H	3.56112800	0.64855800	2.25747000
H	4.88704600	-0.53240300	2.10678700
H	4.98948700	-2.44539300	0.16863600
H	3.69446000	-2.75187900	-1.01153100
H	-2.77861500	-0.47129500	3.50290100

Complex PMe3-D7

Fe	1.38494000	-0.13385300	-0.55821200
Fe	-1.46521300	0.22601700	0.56235400
S	0.54042100	1.17434300	1.33754200
S	-0.53760800	0.76191000	-1.57092600
P	3.05482200	-0.77681400	0.79008800
P	-2.99570300	-1.09379600	-0.46172000
O	1.13594300	-2.70364200	-1.88576600
O	3.22223300	0.99469200	-2.47672500
O	-1.66282600	-1.29680800	3.05342500
O	-3.29714700	2.44790600	1.26990500
C	1.07306700	-1.67276100	-1.09007300
C	2.48356500	0.63528500	-1.65777100
C	-1.58562400	-0.71044200	2.06323700
C	-2.61410700	1.57212700	0.95893600
C	0.47926600	2.95186000	0.81938500
C	0.65530600	3.23296700	-0.66907700
C	-0.37611500	2.60878200	-1.60621200
C	4.28519900	-1.94379200	0.06998200
C	4.12527700	0.57413500	1.43504900
C	2.50416000	-1.63995500	2.31585300
C	-2.38376100	-2.56996800	-1.38154000
C	-4.00801000	-0.23819100	-1.74306600
C	-4.29676700	-1.82561000	0.62135500
H	-4.81513900	-1.02930400	1.15926200
H	1.27431700	3.44951300	1.37922300
H	-0.47314200	3.34598800	1.18020500
H	1.65796900	2.92883000	-0.97743500
H	0.60753200	4.32188100	-0.81059200
H	-1.37526900	2.99551800	-1.39023800
H	-0.13271900	2.86823500	-2.63853900
H	5.06422300	-2.19361400	0.79463400
H	3.78201100	-2.86186900	-0.23904900
H	4.75137700	-1.49110100	-0.80761600
H	4.59442900	1.09820900	0.60022200
H	3.50591000	1.28167800	1.98702900
H	4.90314000	0.18116800	2.09515200
H	3.35798900	-1.91271600	2.94148200
H	1.83974700	-0.98114000	2.87658000
H	1.94903900	-2.53811500	2.04127100
H	-3.21314300	-3.13722000	-1.81335000
H	-1.71606900	-2.24660100	-2.18068700
H	-1.81646900	-3.21337500	-0.70729800
H	-4.55373700	0.58785300	-1.28302200

H	-3.34741500	0.16909200	-2.50953500
H	-4.72244900	-0.92429400	-2.20603000
H	-5.02477800	-2.39181700	0.03493900
H	-3.83905200	-2.48922000	1.35683900
H	1.66263700	-2.51852600	-2.68554600

Complex PMe3-D8

Fe	1.37437500	-0.18744300	-0.53569500
Fe	-1.49154700	0.24824600	0.55943200
S	0.52365700	1.20330300	1.30398000
S	-0.54716400	0.67283100	-1.58517300
P	3.11310800	-0.67168900	0.78398300
P	-3.02338500	-1.08904100	-0.43137300
O	1.31048000	-2.84837000	-1.64137500
O	3.13498100	0.79217900	-2.60162300
O	-1.72292700	-1.17276500	3.10739000
O	-3.29229500	2.52901000	1.15270400
C	1.09523900	-1.77566200	-0.92083000
C	2.43020000	0.48630600	-1.73555200
C	-1.63324800	-0.62372400	2.09685900
C	-2.62484000	1.62635300	0.88874500
C	0.48312400	2.95733700	0.70930700
C	0.67273400	3.16682400	-0.78951900
C	-0.36194600	2.51401700	-1.70380100
C	4.38905200	-1.78625600	0.06366000
C	4.11713500	0.76850800	1.33821200
C	2.67016000	-1.49702100	2.36653600
C	-2.40224100	-2.59158300	-1.30827500
C	-4.02457700	-0.27217600	-1.74558100
C	-4.33194300	-1.79967200	0.65687400
H	-4.85822800	-0.99111700	1.16815400
H	1.27952400	3.47220900	1.25144000
H	-0.46737300	3.37739800	1.04479400
H	1.67253500	2.83204500	-1.07525600
H	0.64254300	4.24836000	-0.98271700
H	-1.35661400	2.92487800	-1.51324000
H	-0.10777300	2.72079200	-2.74534800
H	5.19521700	-1.97745300	0.77634100
H	3.93180600	-2.73412200	-0.22432500
H	4.80801700	-1.32738900	-0.83396700
H	4.52152400	1.28847800	0.46768300
H	3.47496800	1.45678100	1.88858400
H	4.94285300	0.45036900	1.98013300
H	3.55669100	-1.67977700	2.97957600
H	1.97489000	-0.86056100	2.91574400
H	2.17390500	-2.44534000	2.15389100
H	-3.22019300	-3.15256800	-1.76877900
H	-1.69547100	-2.28691700	-2.08182800
H	-1.88816300	-3.23338800	-0.58943700
H	-4.57238300	0.56677100	-1.31209700

H	-3.35727500	0.11325100	-2.51743000
H	-4.73668700	-0.96989900	-2.19445600
H	-5.05291400	-2.38426000	0.07989900
H	-3.87843700	-2.43990700	1.41540500
H	0.77873500	-3.59662000	-1.32654500

Complex PMe3-D9

Fe	1.43496100	0.15981800	-0.56753600
Fe	-1.44393800	0.00247800	0.59376700
S	0.54234900	0.84979900	1.54503000
S	-0.54603000	1.10088200	-1.39490600
P	2.99800000	-1.02366600	0.57047900
P	-3.13360000	-0.67860400	-0.70896000
O	1.39891000	-1.78650400	-2.75604400
O	3.34850400	2.05502500	-1.80480900
O	-0.83405400	-2.75432700	1.21523300
O	-3.07886800	0.59966500	2.91951900
C	1.40565800	-1.03057400	-1.88490900
C	2.63528400	1.32925300	-1.26202400
C	-0.99545000	-1.65011500	0.89490200
C	-2.42411500	0.57055300	1.79501500
C	0.26234500	2.67735300	1.53290300
C	0.32738800	3.35927100	0.17030400
C	-0.67281900	2.87886900	-0.87766100
C	4.17554300	-2.01224800	-0.44714600
C	4.13410600	0.03949400	1.55811200
C	2.43824500	-2.25300100	1.82336300
C	-2.66829900	-1.64115800	-2.20568500
C	-4.15537300	0.68837400	-1.39254000
C	-4.38841200	-1.76466900	0.09198300
H	-4.85022800	-1.23517900	0.92765000
H	1.03190200	3.09531500	2.18620400
H	-0.71027400	2.83954700	2.00191800
H	1.34127700	3.28424500	-0.22816700
H	0.14240800	4.43078700	0.33140000
H	-1.69520900	3.02456800	-0.52123400
H	-0.54377900	3.46139500	-1.79230300
H	4.93498400	-2.48900400	0.17795500
H	3.63217200	-2.78296000	-0.99627200
H	4.66819300	-1.36194400	-1.17263600
H	4.65679600	0.72923700	0.89301100
H	3.54653600	0.62253800	2.26851700
H	4.86824900	-0.56109000	2.10184000
H	3.29500600	-2.71684300	2.31914200
H	1.82019800	-1.75052300	2.56897800
H	1.83453200	-3.02511700	1.34514600
H	-3.55268300	-1.90387900	-2.79206900
H	-1.99179500	-1.04371600	-2.81746100
H	-2.15060300	-2.55462700	-1.90806200
H	-4.59305600	1.25700300	-0.57060800

H	-3.51413700	1.35278500	-1.97262100
H	-4.95117800	0.30185000	-2.03478200
H	-5.16754600	-2.05690200	-0.61642100
H	-3.90403600	-2.66393300	0.47741400
H	-2.94195300	-0.20936600	3.44578500

Complex PMe3-D10

Fe	-1.43506700	0.20438700	0.56671300
Fe	1.38977500	-0.09224100	-0.58243900
S	-0.54597600	0.79141500	-1.58722200
S	0.55044200	1.18833700	1.32505200
P	-2.93566400	-1.11770700	-0.48695200
P	3.03637200	-0.78649700	0.76803700
O	-1.41636100	-1.55022800	2.90933500
O	-3.43048100	2.13221600	1.61201300
O	1.24044700	-2.56766000	-2.11095600
O	3.22153600	1.36944500	-2.27333900
C	-1.41461200	-0.86989600	1.97756400
C	-2.67232300	1.39695900	1.14864400
C	1.16423700	-1.58852600	-1.25621800
C	2.47859900	0.86995900	-1.53638500
C	-0.29294700	2.62329100	-1.65706800
C	-0.35738100	3.36871400	-0.32761400
C	0.64745500	2.94530000	0.74066100
C	-4.13546000	-2.01579700	0.58775800
C	-4.05572700	-0.22165800	-1.64398400
C	-2.28075800	-2.46991200	-1.55370500
C	2.46475700	-1.72373100	2.24188700
C	4.08504000	0.53773700	1.49852400
C	4.29358800	-1.90985100	0.02425700
H	4.77205200	-1.41458100	-0.82331000
H	-1.07554200	2.99722400	-2.32119800
H	0.66650100	2.78793100	-2.15178800
H	-1.36904500	3.30230100	0.07735500
H	-0.18318500	4.43328100	-0.53856400
H	1.66976300	3.09730100	0.38519400
H	0.50533900	3.56035400	1.63173800
H	-4.85086500	-2.58428200	-0.01191600
H	-3.60277300	-2.70066900	1.24947900
H	-4.68019100	-1.30102500	1.20784400
H	-4.61197100	0.53996900	-1.09453300
H	-3.45753000	0.27278200	-2.41045200
H	-4.76198800	-0.90667900	-2.12069200
H	-3.09511800	-3.03732600	-2.01262600
H	-1.65669400	-2.03836800	-2.33697500
H	-1.66054500	-3.14144100	-0.95831700
H	3.30994100	-2.02031200	2.86872300
H	1.78695200	-1.09739700	2.82290100
H	1.92065600	-2.61159900	1.91684600
H	4.59769300	1.07968500	0.70160700

H	3.44637200	1.23559900	2.04072200
H	4.82776800	0.11894600	2.18249800
H	5.06045100	-2.18252400	0.75363300
H	3.80689200	-2.81835800	-0.33514900
H	1.76264900	-2.32531300	-2.89805300

Complex PMe3-D11

Fe	-1.44390600	0.20656200	0.56535600
Fe	1.38544700	-0.11512800	-0.57772300
S	-0.55056200	0.75313500	-1.59322100
S	0.54262900	1.19775700	1.30951200
P	-2.96060700	-1.09905900	-0.47297800
P	3.06904200	-0.73737200	0.76002500
O	-1.40554600	-1.55342400	2.90478700
O	-3.42144000	2.14996400	1.61433800
O	1.38065600	-2.61000100	-2.05077100
O	3.18668000	1.31115900	-2.33059000
C	-1.41379900	-0.86762200	1.97659400
C	-2.67241100	1.40684500	1.14872600
C	1.18557400	-1.63686000	-1.20120800
C	2.45727300	0.82358100	-1.57537500
C	-0.30251800	2.58453300	-1.69308400
C	-0.36815900	3.35162500	-0.37612700
C	0.63629700	2.94642200	0.69961100
C	-4.21909500	-1.91027000	0.60336700
C	-4.01567200	-0.22950100	-1.70805500
C	-2.31988100	-2.52381900	-1.45798000
C	2.55652600	-1.64934000	2.27210600
C	4.09022600	0.63632300	1.43677300
C	4.34332300	-1.83667500	0.01280900
H	4.78967500	-1.34038900	-0.85109800
H	-1.08597400	2.94581700	-2.36312700
H	0.65619000	2.74272600	-2.19113400
H	-1.38014200	3.29166000	0.02924600
H	-0.19421100	4.41267500	-0.60426200
H	1.65854600	3.09464800	0.34229800
H	0.49236700	3.57479700	1.58104800
H	-4.93271700	-2.48910300	0.01157300
H	-3.72844800	-2.57259200	1.31847200
H	-4.76157500	-1.14781800	1.16608600
H	-4.55279600	0.58371200	-1.21642500
H	-3.37912400	0.19667000	-2.48440500
H	-4.73816500	-0.91232000	-2.16308000
H	-3.13351300	-3.07759100	-1.93440400
H	-1.64356900	-2.14630800	-2.22667300
H	-1.76843300	-3.19455700	-0.79547600
H	3.42299800	-1.91084900	2.88520400
H	1.87869700	-1.02262300	2.85295600
H	2.02419600	-2.55765200	1.98606200
H	4.55794300	1.18092900	0.61434000

H	3.44266500	1.32110300	1.98517200
H	4.86859200	0.25877600	2.10509300
H	5.12956000	-2.07758500	0.73269300
H	3.87765800	-2.76097200	-0.33269500
H	0.85100700	-3.39126700	-1.82333100

Complex PMe3-D12

Fe	1.16479700	0.02712200	-0.49595500
Fe	-1.19785100	0.14409000	0.55321100
S	0.64780600	1.29945200	1.44119600
S	-0.64404300	1.13154400	-1.51452200
P	3.05560900	-0.78955600	0.42558300
P	-2.76297100	-1.12203400	-0.55452200
O	0.16701700	-2.65009400	-0.21736100
O	2.71489000	0.30950600	-2.65576800
O	-1.31789400	-1.44846100	3.00290600
O	-3.09392600	2.18419900	1.41670700
C	0.30658300	-1.47867500	-0.19618500
C	1.82749700	-0.41086500	-2.22359800
C	-1.26773300	-0.83669100	2.03513500
C	-2.36155200	1.37373900	1.06289400
C	0.51547800	3.09526100	1.02437200
C	0.64044800	3.45569000	-0.45202000
C	-0.50429300	2.96313700	-1.33059500
C	3.76028800	-2.29620000	-0.36055600
C	4.44359800	0.41622700	0.34347100
C	2.98577200	-1.25603700	2.20472800
C	-2.30467000	-1.79823000	-2.20388000
C	-4.31147900	-0.20187100	-0.93745900
C	-3.37352900	-2.61219900	0.33502400
H	-3.81008500	-2.32503000	1.29332900
H	1.32257500	3.56364400	1.59141900
H	-0.42683600	3.46506400	1.43592200
H	1.59234700	3.08667900	-0.84861700
H	0.67334400	4.55121000	-0.52782900
H	-1.46386200	3.33788100	-0.96390000
H	-0.37354700	3.33028100	-2.35016600
H	4.64940500	-2.64147800	0.17324000
H	3.00978500	-3.08918900	-0.35940300
H	4.02764500	-2.07314900	-1.39436500
H	4.56001700	0.74282300	-0.69155500
H	4.20260800	1.28497300	0.95878000
H	5.37906100	-0.02631200	0.69589100
H	3.96616600	-1.57920300	2.56426400
H	2.64348500	-0.40219900	2.79031900
H	2.27303800	-2.07246500	2.33760000
H	-3.17597300	-2.26462000	-2.67046500
H	-1.94369200	-0.98824900	-2.83834100
H	-1.51488900	-2.54019700	-2.09226100
H	-4.78221700	0.14573600	-0.01642700

H	-4.06787500	0.66718400	-1.55098200
H	-5.01768000	-0.83561700	-1.47935000
H	-4.12744400	-3.13635400	-0.25728900
H	-2.53331900	-3.28293800	0.52216600
H	1.44842900	-1.23295600	-2.88321200

Complex PMe3-D13

Fe	1.40247300	0.11116000	-0.58969300
Fe	-1.37751900	0.05725900	0.56927700
S	0.60712200	0.94579700	1.52406300
S	-0.55283100	1.09663500	-1.41692200
P	3.03706700	-1.01423300	0.50260400
P	-2.99535600	-0.94141600	-0.67147200
O	0.58884400	-2.17484500	-1.38891700
O	3.28197400	1.63964600	-2.12967400
O	-1.33942300	-2.11418900	2.53474500
O	-3.21700700	1.87138400	2.00164800
C	1.23265800	-1.23974300	-1.85139100
C	2.52171500	1.03373700	-1.50122600
C	-1.35367800	-1.27179100	1.75001000
C	-2.51713900	1.16821200	1.41559300
C	0.39984300	2.78187900	1.39093200
C	0.50891600	3.37732600	-0.00731800
C	-0.52811100	2.90947500	-1.02404000
C	4.18851300	-2.01676700	-0.52702800
C	4.17161900	0.02470300	1.50921900
C	2.38919100	-2.24194600	1.70712400
C	-2.48976100	-1.92877400	-2.13971600
C	-4.18253300	0.26063700	-1.40707600
C	-4.11517000	-2.09992000	0.22300400
H	-4.55840700	-1.59498500	1.08356000
H	1.18278200	3.19910900	2.02758600
H	-0.56125100	3.02921500	1.84607200
H	1.51363400	3.20538600	-0.40073600
H	0.39891500	4.46677500	0.08918100
H	-1.53646900	3.16592000	-0.69075900
H	-0.35444700	3.40934800	-1.97877500
H	4.94353100	-2.51302100	0.08805100
H	3.61983100	-2.77393300	-1.06913200
H	4.68928900	-1.37399900	-1.25402000
H	4.69341000	0.73177000	0.86109000
H	3.58191100	0.58754100	2.23367900
H	4.90766100	-0.58857000	2.03528000
H	3.20555700	-2.78661600	2.18860200
H	1.80125700	-1.72687400	2.46816300
H	1.74009000	-2.94441200	1.18212000
H	-3.36537700	-2.36956600	-2.62425600
H	-1.97664200	-1.27646100	-2.84771900
H	-1.79208600	-2.71083100	-1.84076100
H	-4.69884100	0.80356400	-0.61347100

H	-3.63249300	0.97816300	-2.01785800
H	-4.92159800	-0.25029900	-2.02952100
H	-4.91368300	-2.45697300	-0.43205900
H	-3.54505500	-2.95599100	0.58785600
H	1.56387400	-1.29549800	-2.91354100

Complex PMe3-D14

Fe	-1.32260000	0.12184700	0.57562300
Fe	1.27349700	-0.06370400	-0.58690200
S	-0.63580400	0.90726900	-1.55339200
S	0.61893500	1.19100000	1.35544400
P	-2.96211900	-1.01532500	-0.48922900
P	3.03238100	-0.85442500	0.58244800
O	-0.95708000	-2.06266600	2.49135500
O	-3.16347500	1.76131400	2.18763600
O	0.98272700	-2.56097900	-2.01251000
O	2.83810200	1.79119800	-1.60439200
C	-1.08018600	-1.20445600	1.73109900
C	-2.47272200	1.12659500	1.52008300
C	1.09630400	-1.54874200	-1.46363800
C	2.37921800	0.69490600	-1.87346400
C	-0.36524600	2.73935100	-1.51136200
C	-0.47449000	3.40053200	-0.14450900
C	0.58692100	2.98066600	0.86483700
C	-4.04974300	-2.06164200	0.56739600
C	-4.17346300	0.07906500	-1.33994400
C	-2.46520700	-2.17439000	-1.82967700
C	2.63166600	-1.77373200	2.12655100
C	4.17352800	0.46526100	1.16223700
C	4.16579300	-2.01234600	-0.29579300
H	4.57822400	-1.52209800	-1.18011600
H	-1.11887400	3.14688100	-2.18773300
H	0.61816900	2.92280100	-1.94689900
H	-1.47336500	3.24139400	0.26972300
H	-0.37073300	4.48466900	-0.29168600
H	1.58437200	3.19660200	0.47691300
H	0.44947600	3.52863100	1.79876500
H	-4.83864900	-2.53100800	-0.02561000
H	-3.45985000	-2.84047300	1.05319800
H	-4.50802700	-1.44701000	1.34476600
H	-4.64527700	0.74007900	-0.61057300
H	-3.64613700	0.69146900	-2.07219800
H	-4.94630700	-0.50612200	-1.84505600
H	-3.34440200	-2.63008800	-2.29263900
H	-1.90274100	-1.62847100	-2.58814700
H	-1.82407400	-2.96015500	-1.42764100
H	3.54442900	-2.08684200	2.64014000
H	2.04960100	-1.13063200	2.78774200
H	2.03420400	-2.65581500	1.88989200
H	4.46701900	1.08597800	0.31478100

H	3.64868000	1.09600000	1.88066300
H	5.06200800	0.03667100	1.63295900
H	4.98937200	-2.32078600	0.35272900
H	3.61856000	-2.89850000	-0.62189200
H	2.61055900	0.25170600	-2.86954500

Complex PMe3-D15

Fe	-1.37757800	0.06920500	0.56844700
Fe	1.40192700	0.08648400	-0.60856900
S	-0.57194100	1.05099300	-1.46311900
S	0.58678600	0.98579100	1.47963800
P	-2.98157700	-0.96035500	-0.65421000
P	3.07199400	-0.96898500	0.50574600
O	-1.13606600	-2.24867200	2.34361500
O	-3.26192800	1.58771400	2.25871500
O	0.71725400	-2.12721300	-1.51442300
O	3.21261600	1.90340400	-1.89581200
C	-1.21232000	-1.34420000	1.63381100
C	-2.55424500	1.01124600	1.55548200
C	1.36174100	-1.17958900	-1.95220400
C	2.48091600	1.17674500	-1.36920700
C	-0.38412000	2.87555900	-1.19998300
C	-0.47254100	3.36694100	0.24028100
C	0.57497300	2.82221000	1.20613100
C	-4.05460100	-2.15054100	0.25508500
C	-4.20914400	0.21077500	-1.37125700
C	-2.45804600	-1.93239700	-2.12585100
C	2.46699200	-2.19364100	1.73598400
C	4.16900000	0.12499100	1.49484100
C	4.25664700	-1.95426200	-0.50361300
H	4.74782200	-1.30581400	-1.23218600
H	-1.17921600	3.33227700	-1.79248000
H	0.56824400	3.16137300	-1.65089500
H	-1.47285700	3.16877600	0.63153300
H	-0.36252600	4.46045100	0.22165000
H	1.57991400	3.09163500	0.87217000
H	0.42165100	3.26138200	2.19380800
H	-4.84385800	-2.54085400	-0.39217300
H	-3.45268600	-2.98326700	0.62260100
H	-4.51076300	-1.65524200	1.11448000
H	-4.71889400	0.74668000	-0.56864900
H	-3.68632400	0.93619100	-1.99618800
H	-4.95033200	-0.31826000	-1.97583000
H	-3.32262600	-2.39923200	-2.60571100
H	-1.96708600	-1.26461800	-2.83481500
H	-1.73657200	-2.69479700	-1.83146300
H	3.30299800	-2.69979500	2.22578900
H	1.86446500	-1.68373300	2.48878700
H	1.84093400	-2.93178500	1.23219400
H	4.68632000	0.82231100	0.83269900

H	3.55595300	0.69747100	2.19201700
H	4.90985800	-0.45508500	2.05079400
H	5.01809000	-2.42788900	0.12141900
H	3.70908400	-2.72796000	-1.04447400
H	1.72501100	-1.21147600	-3.00302200

Complex CN-A1

Fe	-1.37257300	-0.44115300	-0.00548700
Fe	1.39170600	-0.30200900	-0.00726900
S	-0.01119600	0.68701000	-1.59811500
S	-0.07630400	0.68259900	1.63238400
O	-2.56820700	-2.10963400	2.05890400
O	3.86328700	1.29230500	-0.05422000
C	-2.06232100	-1.42948000	1.23800500
C	2.03238900	-1.40386800	-1.17987200
C	-2.78160900	0.90288400	-0.05285900
C	2.79722800	0.77353000	0.01985100
C	-0.24227200	2.49741100	-1.28270900
C	-0.32544900	2.48631100	1.30399400
C	0.33349800	3.03871500	0.03227800
H	-1.41043600	2.65632900	1.27097300
H	0.09037500	3.00336200	2.18142000
H	0.18601700	4.13674200	0.02913800
H	1.41661800	2.85760400	0.06670400
H	0.23430200	3.00979700	-2.13134300
H	-1.32612800	2.67277900	-1.32125400
N	-3.66122400	1.70017400	-0.08785300
C	-2.00950100	-1.44476300	-1.26219100
O	-2.45114000	-2.14473300	-2.10560000
C	2.05375400	-1.41490700	1.38889900
N	2.50800700	-2.11424300	2.23220000
O	2.53398000	-2.12571800	-1.97117800
H	-0.01336400	-1.36242200	0.02170300

Complex CN-A2

Fe	1.40410800	-0.29437600	-0.00898800
Fe	-1.35572700	-0.43314200	-0.00466900
S	-0.04403100	0.67894100	1.62712200
S	0.00447600	0.71518500	-1.59469400
O	2.60267000	-2.04584200	-2.00252400
C	2.08702000	-1.34920100	-1.19795800
C	-1.98605300	-1.47968900	1.22637500
C	2.74420200	0.85853600	0.03124400
C	-2.90364500	0.76647700	-0.02895900
C	-0.00201300	2.50587900	1.32772200
C	0.05385800	2.53653700	-1.26516400
C	-0.62076600	3.02249800	0.02306900
H	1.11794400	2.81925700	-1.27423100
H	-0.43762900	3.00794800	-2.12829800
H	-0.54802300	4.12828500	0.03710900
H	-1.68530900	2.76337600	-0.00323900
H	-0.52978600	2.96094500	2.17797800
H	1.05971700	2.79309300	1.38778200
C	2.10308400	-1.40194200	1.37515100
C	-1.95196500	-1.46035200	-1.26477100
O	-2.45124500	-2.19595400	2.03950400
O	-2.36134800	-2.17223300	-2.11328000
O	3.76676800	1.45976900	-0.02867900
N	-3.90734300	1.40092400	-0.04610100
N	2.58636800	-2.09746800	2.20542900
H	0.04781700	-1.34600400	0.00344300

Complex CN-A3

Fe	-1.16065000	-0.50551500	-0.00612500
Fe	1.50882400	0.09068000	-0.01482400
S	-0.05329600	0.84855200	-1.59896200
S	-0.04837400	0.79209300	1.62941200
O	-2.16263900	-2.30511200	2.05926300
O	0.93563100	-2.79511800	0.00798300
C	-1.71737300	-1.58129800	1.24094200
C	2.66528600	-0.24539400	-1.23951100
C	-2.78096600	0.59181400	-0.00483100
C	0.82507700	-1.60936700	0.01932900
C	-0.54827100	2.59893600	-1.24652900
C	-0.55427000	2.54912500	1.33463400
C	-0.00912700	3.19856500	0.05742900
H	-1.65295300	2.55954400	1.33180700
H	-0.19290100	3.10502200	2.21189300
H	-0.29427300	4.26909100	0.07603300
H	1.08906200	3.14016700	0.06075000
H	-0.18064900	3.18379900	-2.10231400
H	-1.64691000	2.61119200	-1.24981700
N	-3.77505200	1.24154500	-0.00862400
C	-1.69820500	-1.55671200	-1.28113600
O	-2.11838100	-2.26556900	-2.12685600
C	2.74282300	-0.32461900	1.36630700
N	3.56312900	-0.55188000	2.19224600
O	3.48304300	-0.42562000	-2.07317300
H	2.28520300	1.44397600	0.02621700

Complex CN-A4

Fe	-1.40985700	-0.37200100	-0.00535600
Fe	1.60289600	-0.02550600	-0.01835800
S	-0.03167200	0.74720500	-1.55238300
S	-0.01079300	0.66758200	1.58808800
O	-2.25736800	-2.30353700	2.01156400
O	1.16509300	-2.93829700	-0.00502700
C	-1.89327200	-1.51599300	1.20703500
C	2.79071800	-0.14251300	-1.25716700
C	-3.05127800	0.69837200	0.00716500
C	1.19367400	-1.76168600	0.00639400
C	-0.06532200	2.57592100	-1.23770800
C	-0.05447100	2.50830900	1.36790300
C	-0.71256600	3.01930700	0.07989000
H	-0.61508900	2.89932400	2.22923500
H	0.99037300	2.84004700	1.44362500
H	-1.76907200	2.72347400	0.07562200
H	-0.68219800	4.12674300	0.10736000
H	0.97915200	2.91274000	-1.30048000
H	-0.63072100	3.00917000	-2.07539800
N	-4.07071200	1.30910000	0.00795100
C	-1.88216700	-1.47232900	-1.26113600
O	-2.22915600	-2.23232200	-2.10038500
C	2.88485100	-0.23763200	1.36731900
N	3.72931900	-0.33577200	2.19361300
O	3.61753200	-0.16024600	-2.09947500
H	2.14791000	1.41848200	0.02275600

Complex CN-A5

Fe	1.26715400	-0.18187500	0.01719200
Fe	-1.36364300	-0.51238300	-0.30608600
S	-0.39527300	0.40064100	1.64397900
S	-0.04887300	0.98393300	-1.56828100
O	3.07361400	-1.05891200	-2.09837900
O	0.19370900	-2.91383700	-0.96592000
C	2.31376600	-0.71564300	-1.26314500
C	2.40221100	1.34512800	0.44520900
C	-0.12859000	-1.81585600	-0.64099500
C	-0.43872200	2.25128000	1.61801500
C	-0.18257600	2.71617100	-0.92284300
C	-1.01852200	2.90226500	0.35299900
H	0.84703700	3.06074200	-0.75609900
H	-0.62771900	3.30154600	-1.74061300
H	-1.08607900	3.99172600	0.54259800
H	-2.04224400	2.54165300	0.18545600
H	-1.05374500	2.53588500	2.48500300
H	0.58937700	2.59842000	1.78464400
N	3.14057700	2.24290700	0.68626500
C	1.99620700	-1.24533400	1.18620000
O	2.56012500	-1.93268900	1.96084000
C	-2.25229300	-1.81323000	0.73997300
N	-2.86965900	-2.61443900	1.36069100
C	-2.92002900	0.30240600	-0.51480000
O	-4.02146200	0.65581600	-0.76863000
H	-1.98462200	-1.09787000	-1.55658900

Complex CN-A6

Fe	-1.49030600	-0.46464500	-0.26274300
Fe	1.39691500	-0.16923800	0.04610400
S	-0.00211600	0.86272700	-1.55159200
S	-0.28190100	0.43067900	1.58919400
O	-0.34309300	-3.05906200	-1.00852600
C	-2.58823700	-1.46343300	0.91330700
C	-2.85647900	0.61090600	-0.52055000
C	-0.63984200	-1.98065500	-0.64050500
C	-0.34244600	2.61895100	-1.05679300
C	-0.61019400	2.25521100	1.49943900
C	0.07603400	3.02077900	0.36223100
H	-1.70372200	2.36711100	1.44662500
H	-0.26830800	2.66502700	2.46062200
H	-0.16755800	4.09487500	0.48661700
H	1.16358200	2.91879700	0.46325700
H	0.20894200	3.23518800	-1.78116100
H	-1.41814900	2.79127200	-1.21089300
C	2.25944700	-0.93967600	-1.24777300
C	2.76879900	1.17933500	0.40345800
N	3.64072000	1.95442000	0.62951800
N	-3.32901900	-2.07719900	1.60743000
O	-3.84875800	1.17194200	-0.83586200
O	2.88183700	-1.46080100	-2.10892300
C	1.97500800	-1.36048700	1.17124800
O	2.42085600	-2.16256300	1.91746600
H	-2.16352200	-0.98806500	-1.51032400

Complex CN-A7

Fe	-1.48247400	-0.58018100	0.30260000
Fe	1.39532300	-0.06224700	-0.06391900
S	-0.35227700	0.41673300	-1.54156800
S	-0.05084900	0.84009300	1.59618400
O	0.01384200	-3.00049700	0.97966700
C	-2.94161000	0.67796200	0.42242100
C	-0.41085500	-1.96557400	0.64958900
C	-0.81594600	2.21187400	-1.42074300
C	-0.55023000	2.56129700	1.13053200
C	-0.16094400	2.99403100	-0.28302900
H	-1.63260600	2.62707200	1.27288000
H	-0.05312000	3.21405300	1.85909400
H	-0.45122600	4.05506100	-0.39845800
H	0.92782600	2.94510600	-0.38873700
H	-0.53304600	2.65799800	-2.38214900
H	-1.90583600	2.23610900	-1.32064300
N	-3.86651600	1.40081200	0.51043500
C	-2.51376000	-1.60328700	-0.62924500
O	-3.21975400	-2.31155400	-1.23643000
C	2.44177600	-0.77183200	1.36489600
N	3.13063800	-1.18962400	2.22116600
C	2.60028300	1.13152600	-0.49946800
O	3.50879900	1.78569700	-0.87703900
C	1.97316000	-1.31890600	-1.13484600
O	2.43193600	-2.10761500	-1.87076800
H	-2.09470700	-1.05060400	1.59312800

Complex CN-A8

Fe	1.35948700	-0.61623500	-0.29728600
Fe	-1.26974100	-0.08048500	0.03496300
S	0.42202400	0.39256500	1.62540900
S	0.13848400	0.96936500	-1.57878000
O	-0.39306800	-2.85087900	-0.97467700
C	3.06406900	0.30305100	-0.51666900
C	-0.02377000	-1.77639400	-0.61601900
C	0.61667800	2.23602400	1.60708100
C	0.42416800	2.68575400	-0.94094200
C	1.24714400	2.83528600	0.34546100
H	0.94032800	3.21587500	-1.75389700
H	-0.57193800	3.13824800	-0.81410400
H	2.24268700	2.40238700	0.19067500
H	1.38139100	3.92073600	0.52594600
H	-0.37906600	2.66913400	1.78796200
H	1.25241100	2.46927300	2.47371700
N	4.13945300	0.77485800	-0.69237600
C	2.16115500	-1.86223200	0.57099900
O	2.72953500	-2.73012100	1.13813700
C	-2.44286400	-0.57215400	-1.38889500
N	-3.24708300	-0.84258000	-2.21651400
C	-2.32777800	1.28561500	0.43211500
O	-3.19440400	2.03164300	0.73811500
C	-2.06383700	-1.18483400	1.11389900
O	-2.66131500	-1.89039300	1.84725400
H	1.81030800	-1.20045100	-1.60654700

Complex CN-A9

Fe	-1.39913900	-0.62107800	-0.31632000
Fe	1.31478200	-0.05213500	0.04612600
S	-0.06473100	0.90901300	-1.57741300
S	-0.43726700	0.41872000	1.59501500
O	0.19239900	-2.95986100	-1.03657300
C	-2.93390400	0.54874500	-0.47929000
C	-0.21466000	-1.91171900	-0.66765400
C	-0.59010000	2.61266500	-1.06743800
C	-0.94796500	2.19661100	1.46353700
C	-0.25938900	3.02635900	0.37182400
H	-2.03589400	2.20460500	1.31167000
H	-0.72398100	2.63726600	2.44626600
H	-0.58652600	4.07785900	0.49509300
H	0.82834800	3.00745700	0.52327600
H	-0.09040800	3.29651400	-1.76942200
H	-1.67427800	2.65820600	-1.23614500
N	-3.90057200	1.22351400	-0.61897100
C	1.97044100	-1.30519000	1.32754700
N	2.45092400	-2.05719600	2.10885100
C	2.48999600	1.18993200	0.47373900
O	3.43730900	1.86910900	0.69932400
C	2.33459300	-0.75183300	-1.17098400
O	3.10731300	-1.16766300	-1.96276100
C	-2.35143300	-1.72414800	0.59419700
O	-3.03162200	-2.48617400	1.18615300
H	-1.97314500	-1.11812700	-1.61848600

Complex CN-A10

Fe	-1.35711500	-0.61607000	-0.28334900
Fe	1.28018400	-0.07315300	0.00345300
S	-0.12415000	0.97529700	-1.56092300
S	-0.42404400	0.38621500	1.63811000
O	0.37492300	-2.86229000	-0.97100400
C	-3.05039000	0.31634400	-0.53340700
C	0.01160000	-1.78708100	-0.60527000
C	-0.40148500	2.69570300	-0.92935800
C	-0.62381800	2.22901400	1.61314300
C	-1.23810000	2.83941000	0.34850000
H	-1.26752700	2.46433700	2.47316700
H	0.37070900	2.66026100	1.80671500
H	-2.23325000	2.41149600	0.17868800
H	-1.37007700	3.92444800	0.53301600
H	0.59485000	3.14348400	-0.79105700
H	-0.90793300	3.22678200	-1.74798300
N	-4.11889400	0.79441100	-0.73302000
C	2.09627300	-1.24178100	1.27572100
N	2.68456000	-1.92926700	2.04144300
C	2.32941700	1.27862800	0.45483500
O	3.20224400	2.04970300	0.67112900
C	2.35469500	-0.61735100	-1.24120700
O	3.14450300	-0.94037100	-2.05595000
C	-2.17783300	-1.85239300	0.58116900
O	-2.76840500	-2.71150500	1.13632700
H	-1.80340500	-1.19849200	-1.59796200

Complex CN-A11

Fe	1.39668700	-0.25294200	0.04039900
Fe	-1.42473200	-0.34807200	-0.02054800
S	-0.00724800	0.73432700	-1.58885600
S	-0.06887300	0.73698300	1.60547800
N	3.55648800	2.03308700	0.09145300
O	-4.03109000	0.75512200	-0.78721200
C	2.73612700	1.17180400	0.07358000
C	-1.13223800	-1.78153800	-0.97894400
C	2.05223800	-1.31353600	-1.15538600
C	-2.92742300	0.47234200	-0.43194300
C	0.00284000	2.56311300	-1.27729700
C	-0.02710500	2.56643200	1.29762400
C	-0.69938800	3.03340300	0.00226100
H	1.03459500	2.85096900	1.30204500
H	-0.52735400	3.02857000	2.16222600
H	-0.70385400	4.14176600	-0.00173600
H	-1.74735000	2.69998100	-0.00746500
H	-0.47913000	3.02193000	-2.15365700
H	1.06296000	2.85321000	-1.25655300
O	2.51488900	-2.07174700	-1.94831700
C	1.98282600	-1.30469600	1.28253400
O	2.42599800	-2.02578500	2.11709800
C	-2.09644800	-1.31369300	1.24839900
O	-2.58856200	-1.97414000	2.10011900
N	-1.04935000	-2.78241200	-1.71124900
H	-0.16118700	-3.29512600	-1.70681200

Complex CN-A12

Fe	-1.50053400	-0.30799000	0.03914800
Fe	1.46644700	-0.19372400	-0.06653600
S	0.03480600	0.57734400	1.66231800
S	-0.08340500	0.78302600	-1.49928700
N	-3.88763800	1.21340300	-0.93525200
O	3.94249100	1.37969700	-0.21768600
C	-2.86191700	0.63899400	-0.49760100
C	2.14199000	-1.42877500	1.21491100
C	-2.40292800	-1.02246900	1.33283800
C	2.88304700	0.84498900	-0.10249700
C	-0.31018100	2.39473300	1.46226500
C	-0.27929600	2.58778100	-1.12163500
C	0.30155000	3.05286700	0.21941400
H	-1.35746700	2.79627700	-1.17083300
H	0.22134200	3.12583100	-1.94116100
H	0.13911300	4.14719500	0.29406400
H	1.38790900	2.88686100	0.22228100
H	0.10132000	2.87798300	2.36222600
H	-1.40284800	2.51759800	1.46433800
O	-3.05212600	-1.49571600	2.20317000
C	-1.31452100	-1.83456800	-0.80299200
O	-1.41135400	-2.85273800	-1.40580000
C	1.97360900	-1.28695500	-1.31862800
O	2.42032100	-1.99226000	-2.15986800
N	2.62860600	-2.19123300	1.98463500
H	-4.41852400	0.71916000	-1.66632300

Complex CN-A13

Fe	-1.29417400	-0.38422700	-0.06066300
Fe	1.27942700	-0.28389700	0.13823400
S	0.05066000	0.84108200	-1.45785200
S	-0.14923600	0.68393100	1.69981400
O	-2.16409100	-2.49865400	1.75504500
O	3.43579800	1.13922300	-1.20517700
C	-1.79032400	-1.63274600	1.04296300
C	1.65928800	-1.52425800	-0.99860000
C	-2.82009200	0.76423200	-0.00058500
C	2.80857300	0.69366400	-0.23991400
C	-0.12955700	2.64926700	-1.10103700
C	-0.38816600	2.50671300	1.44725700
C	0.37550500	3.11470300	0.26745500
H	-1.46832400	2.67207500	1.33579400
H	-0.05340800	2.96899200	2.38741500
H	0.26799000	4.21626400	0.31560500
H	1.44846400	2.89098800	0.35948200
H	0.42453500	3.16193100	-1.89997200
H	-1.20057800	2.87087100	-1.20835300
N	-3.74236400	1.51332600	0.02774400
C	-1.78793400	-1.35290600	-1.40938200
O	-2.16664500	-2.02107400	-2.30930100
C	2.00149500	-1.37197400	1.53614400
N	2.51129700	-2.03539400	2.37782700
O	1.94126000	-2.39087000	-1.75027000
H	3.21614300	0.93520100	0.79463600

Complex CN-A14

Fe	-1.36975400	-0.30087000	0.04494600
Fe	1.43072500	-0.34347100	-0.04599200
S	0.01332000	0.72845100	-1.53051800
S	0.07929500	0.64203500	1.63735300
O	-2.41920900	-2.15478100	2.04132800
O	3.88308700	1.04405400	-0.64811900
C	-1.96294500	-1.40511800	1.25074700
C	1.71733900	-1.69135200	-1.27818300
C	-2.73269800	1.03406000	0.18053500
C	2.86167600	0.48285100	-0.38928600
C	-0.08779100	2.55071100	-1.19029400
C	-0.06244200	2.47693600	1.38415100
C	0.57961300	3.02242300	0.10550400
H	-1.13276400	2.72018000	1.41260400
H	0.42869900	2.92163900	2.26202900
H	0.50971000	4.12801200	0.13504900
H	1.64809900	2.76531800	0.08801900
H	0.38732200	3.03803400	-2.05395700
H	-1.15753100	2.79892600	-1.18321800
N	-3.55128200	1.89282000	0.25219700
C	-2.15113200	-1.19764700	-1.22449700
O	-2.73669600	-1.79195300	-2.05863300
C	2.25905800	-1.43948400	1.25556500
N	2.84478400	-2.12274400	2.03168600
O	0.77258100	-2.47858300	-1.46736900
H	2.67267800	-1.88772800	-1.83299600

Complex CN-A15

Fe	-1.25811600	-0.37922300	-0.00228000
Fe	1.26490600	-0.24217400	-0.00185000
S	0.02537000	0.86378400	-1.58154000
S	-0.02925900	0.72427800	1.62993200
O	-1.97284400	-2.06851000	2.10298600
O	3.84530000	1.10716600	-0.19684400
C	-2.39562300	-1.31381700	1.22347800
C	1.60114100	-1.49983600	-1.16022200
C	-2.70884900	0.86254300	-0.29779200
C	2.78901400	0.58081800	-0.07753700
C	-0.15529700	2.66793400	-1.20932400
C	-0.27822900	2.54606100	1.35729700
C	0.42556000	3.13438100	0.12952500
H	-1.36164300	2.71438300	1.29252100
H	0.10184900	3.03113700	2.26833800
H	0.32883000	4.23710600	0.17452300
H	1.49979200	2.90295500	0.17242300
H	0.34439900	3.19511600	-2.03550500
H	-1.23273200	2.87960900	-1.25263700
N	-3.60583500	1.63388500	-0.40904500
C	-1.58565800	-1.63591400	-1.15573400
O	-1.84783100	-2.48783600	-1.93732500
C	1.85170900	-1.45555800	1.35353600
N	2.32964200	-2.19423100	2.14835800
O	1.89789400	-2.35557200	-1.91492000
H	-3.50421400	-1.17218900	1.12772200

Complex CN-A16

Fe	-1.21286800	-0.32369000	0.02766500
Fe	1.29101400	-0.33015700	-0.07464100
S	0.00471000	0.88298400	-1.56065200
S	0.18679000	0.71832900	1.64486700
O	-1.95868100	-2.48677200	1.83959400
O	3.92522500	0.80052400	-0.63494800
C	-1.62773500	-1.61527100	1.11429200
C	1.35212800	-1.65157400	-1.20233600
C	-2.58419500	0.95677800	0.40158400
C	2.85740000	0.36803400	-0.35201000
C	-0.10448600	2.68468400	-1.14180600
C	0.07057800	2.55347500	1.41589000
C	0.66098500	3.10825300	0.11565100
H	-0.99661600	2.80614100	1.48852500
H	0.59838500	2.98857800	2.27737500
H	0.63645300	4.21423300	0.17258200
H	1.71657000	2.81141100	0.02748000
H	0.28791700	3.21819700	-2.01958800
H	-1.17381200	2.91113500	-1.03278600
N	-3.39455300	1.80548900	0.58433300
C	-2.23006900	-1.15381800	-1.31015900
O	-3.38586800	-1.34302700	-1.67769200
C	1.92294100	-1.54118200	1.25795800
N	2.39090400	-2.29433900	2.04573700
O	1.46356400	-2.56673100	-1.94169700
H	-1.39834200	-1.63695700	-1.92660000

Complex CN-A17

Fe	-1.14733400	-0.42884400	-0.14537400
Fe	1.40631800	-0.02914700	0.02082400
S	-0.10515500	0.78162400	1.54917500
S	0.02664100	0.94137500	-1.63450900
N	-3.75236500	1.24769700	-0.10935600
O	4.21148300	0.53839900	0.29175400
C	-2.76248400	0.58961400	-0.12074600
C	2.26467600	-0.95863500	1.39792700
C	-1.56072700	-1.66754300	0.99701500
C	2.96471000	0.74410000	-0.11940400
C	-0.46716100	2.58454500	1.29465700
C	-0.38526700	2.70609300	-1.26059500
C	0.18309600	3.22508700	0.06444100
H	-1.48134000	2.78759700	-1.27019300
H	0.02415100	3.28769500	-2.10008100
H	0.01350700	4.31923400	0.11370500
H	1.27020800	3.05333900	0.09031500
H	-0.10682100	3.07794100	2.20947300
H	-1.56113900	2.67834200	1.24678200
O	-1.87803500	-2.52927100	1.74300200
C	-1.53624100	-1.47867300	-1.46857400
O	-1.85189300	-2.20121000	-2.35144800
C	1.56893000	-1.44338200	-0.98427600
O	1.80985700	-2.40893300	-1.62375700
N	3.07369900	-1.42880400	2.14000000
H	4.16674600	-0.20507500	0.97859400

Complex CN-A18

Fe	-1.14964100	-0.45573100	-0.12949500
Fe	1.35102400	-0.12180800	0.07736200
S	-0.11498900	0.79638700	1.60449900
S	0.12680200	0.94023200	-1.57760000
N	-3.72857400	1.24736200	-0.31869100
O	3.89507400	1.30064700	-0.06901700
C	-2.74762500	0.58210800	-0.23276800
C	1.91025800	-1.27207700	1.49266400
C	-1.68561800	-1.61724500	1.03040000
C	2.84433200	0.75627100	0.03828500
C	-0.42431000	2.60560400	1.32678100
C	-0.16426900	2.73387300	-1.21688800
C	0.32358000	3.23197300	0.14687300
H	-1.24896600	2.88735600	-1.31027700
H	0.33988300	3.29093500	-2.02082800
H	0.17756500	4.33004400	0.18549600
H	1.40261200	3.04379700	0.24945300
H	-0.12789100	3.10179100	2.26324800
H	-1.50943100	2.72117500	1.19564700
O	-2.10416000	-2.42420800	1.78501700
C	-1.22726800	-1.65186800	-1.36045100
O	-1.61950900	-2.93341700	-1.58366500
C	1.73734600	-1.39276800	-1.04396100
O	2.06265400	-2.26254600	-1.77824200
N	2.33946400	-1.98369800	2.33989900
H	-1.08459900	-3.27090000	-2.32828500

Complex CN-B1

Fe	-1.39442000	-0.40669500	-0.00044000
Fe	1.38328200	-0.38968500	0.00035000
S	-0.01433600	0.66721500	-1.61355700
S	-0.01512500	0.66815500	1.61364700
O	-2.57060400	-2.06738600	2.08291600
N	3.85123300	1.56571600	-0.00490800
C	-2.08342400	-1.38740600	1.24664300
C	2.03249400	-1.40934100	-1.23972200
C	-2.75944900	0.99199200	0.00002200
C	2.87642600	0.88741900	-0.00087100
C	-0.18256900	2.48362500	-1.29777000
C	-0.18299800	2.48438800	1.29696900
C	0.44061400	3.01543600	-0.00046500
H	-1.26195100	2.69037700	1.31997900
H	0.29634300	2.97541700	2.15576800
H	0.30188600	4.11445400	-0.00082900
H	1.51926500	2.81935100	-0.00027900
H	0.29675700	2.97434500	-2.15674300
H	-1.26158100	2.68925700	-1.32101800
N	-3.61930900	1.81118500	0.00105800
C	-2.08333600	-1.38764900	-1.24732100
O	-2.57063500	-2.06790300	-2.08333800
C	2.03323700	-1.40655800	1.24235900
O	2.49208500	-2.10663400	2.07739600
O	2.49026900	-2.11214400	-2.07306900
H	-0.02809600	-1.36045300	0.00065000

Complex CN-B2

Fe	1.26849200	-0.16844000	0.01593100
Fe	-1.37706100	-0.57915200	-0.28917600
S	-0.40249100	0.38895100	1.63092500
S	-0.08949600	0.96815800	-1.57240200
O	3.11556100	-0.91983900	-2.11040100
N	-4.10886400	0.90853700	-0.71913500
C	2.34432300	-0.62516500	-1.27010200
C	-2.21404000	-1.81097400	0.56132900
C	2.35482300	1.40093100	0.45897800
C	-3.05142700	0.40291300	-0.52859200
C	-0.49680600	2.23930100	1.61708500
C	-0.25316100	2.69568600	-0.92302100
C	-1.08923200	2.87104100	0.35180800
H	0.76998100	3.06448100	-0.76809600
H	-0.72006900	3.26316900	-1.74058500
H	-1.17095200	3.96021200	0.53856900
H	-2.10314300	2.48895600	0.18168500
H	-1.12560800	2.49820700	2.48137200
H	0.52288200	2.60582200	1.79606200
N	3.07848000	2.30749000	0.71081700
C	2.03839900	-1.19836700	1.18700500
O	2.60494300	-1.88114700	1.96379000
C	-0.00766600	-1.76853100	-0.58994200
O	0.31124900	-2.86930700	-0.94736400
O	-2.80554000	-2.67416900	1.11346100
H	-1.82680100	-1.15467300	-1.60595500

Complex CN-B3

Fe	1.25951800	-0.13318700	0.03881500
Fe	-1.38409700	-0.60870800	-0.31059400
S	-0.44216800	0.41755400	1.60443700
S	-0.08724500	0.93937000	-1.59308900
O	3.09389100	-0.99780200	-2.05757100
N	-3.94500000	1.18054600	-0.57108300
C	2.32630600	-0.65971400	-1.22983500
C	-2.27746800	-1.80121200	0.53684700
C	2.48238200	1.32951600	0.50630000
C	-2.96235800	0.52441900	-0.45374500
C	-0.89553500	2.21218100	1.49766200
C	-0.59465800	2.63437900	-1.04268500
C	-0.22786500	3.03692700	0.39052600
H	-0.11156100	3.32591700	-1.74764400
H	-1.68292200	2.67688300	-1.18845100
H	0.86056800	3.01527900	0.51762600
H	-0.55733700	4.08617100	0.52585500
H	-1.98866500	2.23551600	1.38579000
H	-0.62690800	2.63861800	2.47461800
N	3.32439600	2.12097300	0.77796000
C	1.95791400	-1.21790900	1.20722000
O	2.47515500	-1.93594600	1.98700900
C	-0.07444000	-1.82012800	-0.61895400
O	0.29984700	-2.90111700	-0.96773400
O	-2.91037500	-2.64097500	1.07958200
H	-1.91308700	-1.13455300	-1.62154800

Complex CN-B5

Fe	1.28472800	0.13607600	-0.06909300
Fe	-1.09177100	-0.67474200	0.00902200
S	-0.27491600	0.82027700	1.58280800
S	-0.36435100	0.82173800	-1.61202300
O	2.99436700	-1.81746300	-1.40193200
N	-4.14255700	-0.13826400	0.10831800
C	2.06366500	-0.86015000	-1.26286700
C	-1.05499500	-1.89696100	1.23502700
C	2.30207200	1.75664500	-0.06511000
C	-2.96863500	-0.31647300	0.06931600
C	-0.80125500	2.57132100	1.28427500
C	-0.88778200	2.56685700	-1.27731400
C	-1.64811600	2.80429000	0.03051400
H	0.03245800	3.16824600	-1.30596300
H	-1.52088600	2.85189700	-2.13013500
H	-1.98716300	3.85924000	0.04060200
H	-2.54337200	2.16896600	0.06155500
H	-1.37042400	2.86318800	2.17896800
H	0.12493600	3.16272700	1.24557500
N	2.93167900	2.76394800	-0.06065200
C	2.17243300	-0.76878900	1.08100600
O	2.88063500	-1.46259200	1.74543300
C	-1.13968200	-1.89540100	-1.22054000
O	-1.17871200	-2.76032200	-2.02534500
O	-1.02677500	-2.76162600	2.04193100
H	3.36962300	-2.07299900	-0.52150200

Complex CN-B6

Fe	-1.24546700	-0.24446200	-0.01560900
Fe	1.22992400	-0.38692400	-0.01589400
S	-0.01442400	0.91511100	-1.57785900
S	0.03732800	0.74645600	1.64597400
O	-2.15074400	-2.28535200	1.86602600
N	3.93838200	1.05638000	-0.64981800
C	-1.75384800	-1.44933600	1.13446000
C	1.32349100	-1.73939200	-1.08938100
C	-2.70461300	0.97084300	0.14146700
C	2.88362400	0.54925600	-0.44926000
C	0.00295800	2.71805100	-1.16268400
C	0.01630800	2.58334300	1.40248200
C	0.71256600	3.10130200	0.13999400
H	-1.04182400	2.87941200	1.41439200
H	0.50794200	2.99597500	2.29542700
H	0.73828600	4.20762400	0.19786500
H	1.75222000	2.74880700	0.11119000
H	0.50151500	3.20359200	-2.01370500
H	-1.04885000	3.03645700	-1.14045200
N	-3.58898900	1.75792700	0.23738800
C	-1.89268700	-1.19996000	-1.31231400
O	-2.37763800	-1.85913000	-2.16458100
C	2.18295600	-1.45470900	1.26102100
O	1.65383600	-2.17243700	2.11673400
O	1.40633300	-2.68329300	-1.80096500
H	3.30379900	-1.41576100	1.21911900

Complex CN-B7

Fe	1.26405300	-0.31464500	-0.02896100
Fe	-1.21637100	-0.32620200	0.00251000
S	0.03759600	0.76267700	1.62155500
S	-0.07665300	0.87779100	-1.60007800
O	1.78053400	-2.49517800	-1.90003700
N	-3.90876400	1.17472800	0.31985300
C	1.56272600	-1.60328100	-1.14912100
C	-1.53379600	-1.58285300	1.16402600
C	2.71549700	0.92418900	-0.36355900
C	-2.86761400	0.61662100	0.19880000
C	0.14075500	2.59413600	1.33898000
C	0.02043100	2.68744300	-1.22479000
C	-0.59879700	3.12366600	0.10618300
H	1.08764900	2.94846600	-1.26274500
H	-0.49711200	3.18703200	-2.05635200
H	-0.56841500	4.23030900	0.14573400
H	-1.65334500	2.82004000	0.14671200
H	-0.27886900	3.05071400	2.24656600
H	1.21136900	2.83584100	1.28184600
N	3.61386000	1.69047600	-0.49306800
C	2.37685000	-1.24855500	1.21493000
O	1.93656800	-1.99197400	2.10021900
C	-1.75528100	-1.36920900	-1.27486500
O	-2.14225700	-2.10032500	-2.11740500
O	-1.78797900	-2.46826300	1.89942500
H	3.48866800	-1.12132000	1.13370200

Complex CN-B8

Fe	-1.30090000	-0.26722500	-0.00252300
Fe	1.24309500	-0.40584200	0.00248600
S	0.02373800	0.79336400	-1.58302200
S	0.01822300	0.79803000	1.57913200
O	-2.26849900	-2.12229700	2.02709100
N	4.00166000	1.02320300	0.00778700
C	-1.86113400	-1.35007100	1.22208600
C	1.60488700	-1.57282600	-1.22320700
C	-2.94937200	0.89622600	-0.01550900
C	2.93070300	0.50765500	0.00574800
C	0.05389600	2.62187700	-1.28270600
C	0.05454900	2.62591300	1.27597900
C	0.76434800	3.07476300	-0.00425100
H	-0.99846300	2.94226000	1.26351200
H	0.55717700	3.05539800	2.15492400
H	0.79280600	4.18266100	-0.00591700
H	1.80247800	2.71736800	-0.00391200
H	0.55359500	3.05163300	-2.16316900
H	-0.99998500	2.93528300	-1.26896900
N	-2.97173200	2.13598400	-0.00087900
C	-1.84425500	-1.36603700	-1.22051400
O	-2.23991200	-2.14988800	-2.02017800
C	1.59697800	-1.57157500	1.23156100
O	1.85352500	-2.39093100	2.04691900
O	1.86703300	-2.39278300	-2.03615800
H	-3.88632500	0.29017000	-0.03812000

Complex CN-B9

Fe	1.29935300	-0.36252900	0.05504000
Fe	-1.24814400	-0.29675400	-0.06756700
S	-0.03072100	0.81847700	1.54892300
S	0.13888300	0.77096100	-1.60922700
O	2.25608100	-2.28804200	-1.91631800
N	-3.86493500	1.08343500	0.29664500
C	1.84932500	-1.49831300	-1.13184400
C	-1.89878000	-1.30731600	1.17951800
C	2.81501300	0.80271200	0.13285300
C	-2.91157300	0.79036900	-0.44018600
C	0.16569900	2.63443000	1.23701600
C	0.29881800	2.59717100	-1.32102000
C	-0.42253100	3.14015100	-0.08297700
H	1.37723400	2.80014000	-1.25866500
H	-0.09897900	3.07474800	-2.22871400
H	-0.34349100	4.24535000	-0.09659400
H	-1.49106600	2.88670200	-0.12643000
H	-0.32989900	3.13145500	2.08347800
H	1.24485200	2.83749900	1.28440900
N	3.73671600	1.55251100	0.17690600
C	1.68371300	-1.48104100	1.31824100
O	1.96907000	-2.26637600	2.15867400
C	-1.59662300	-1.51602800	-1.24081400
O	-1.83792100	-2.38785100	-2.00850300
O	-2.33139200	-2.03646400	2.00546000
H	-2.86819300	1.13988600	-1.50099000

Complex CN-B10

Fe	-1.47827000	-0.23773600	0.00780300
Fe	1.47077200	-0.39593000	-0.03436800
S	0.02444300	0.73612400	-1.52788200
S	0.02887400	0.54283300	1.64291300
O	-2.66913000	-2.07244900	1.94054600
N	3.87655500	0.58075900	-1.50999000
C	-2.16168000	-1.33002900	1.16449100
C	0.96867400	-1.97502400	-0.62451000
C	-2.72581300	1.26246600	0.12539200
C	2.85533600	0.29151200	-0.84165000
C	0.10522000	2.54535200	-1.12367500
C	0.08459000	2.38973100	1.45563200
C	0.79529900	2.90504400	0.19871000
H	-0.96115000	2.72715600	1.47000100
H	0.59834400	2.77012500	2.35146200
H	0.84593700	4.01040600	0.26363900
H	1.82821300	2.52403700	0.18618500
H	0.64000000	3.02103000	-1.95930200
H	-0.93684900	2.89419900	-1.11714000
N	-3.47594200	2.18271300	0.19952300
C	-2.21662200	-1.12102400	-1.28569800
O	-2.76356200	-1.71836700	-2.15334700
C	2.47584200	-1.02272900	1.22552000
O	3.18415200	-1.44278500	2.07931300
O	0.89095400	-3.07254700	-1.06882800
H	4.29873900	1.49814600	-1.31810700

Complex CN-B11

Fe	-1.37515600	-0.21106000	0.01819300
Fe	1.34727200	-0.37537400	-0.01015300
S	0.04022700	0.76647000	-1.59077200
S	0.08716200	0.75497500	1.61132300
O	-2.24935300	-2.08571000	2.07252400
N	4.02817000	1.25012800	0.01653800
C	-1.86973100	-1.32142500	1.24942600
C	1.77049400	-1.51333400	-1.24309100
C	-2.77110200	0.92259700	0.00011900
C	2.99142000	0.66743500	0.00481700
C	-0.02670300	2.59004700	-1.26903600
C	0.02276400	2.57937300	1.30225100
C	0.67842200	3.06627500	0.00572600
H	-1.04482800	2.85057600	1.31924100
H	0.52358300	3.04229800	2.16513700
H	0.65805600	4.17478800	0.01060000
H	1.73127600	2.75527100	-0.01434300
H	0.43733700	3.06424500	-2.14678000
H	-1.09469200	2.85591500	-1.24235400
N	-3.95792900	1.31235500	-0.07927500
C	-1.84786800	-1.34028300	-1.20253100
O	-2.21841800	-2.11031900	-2.02638200
C	1.75511000	-1.55226700	1.19116600
O	2.06500700	-2.37143000	1.99157600
O	2.08681700	-2.30397100	-2.06944600
H	-4.70490800	0.60462600	-0.16947400

Complex CN-C1

Fe	-1.36846500	-0.23512700	-0.27122700
Fe	1.39640200	-0.19293500	-0.25818200
S	-0.01139300	1.67642700	-0.34404400
S	-0.00165500	-0.93913600	1.54899900
N	-2.64515300	-3.04486800	-0.38480600
O	3.88774700	0.87105000	0.88607300
C	-2.09893300	-1.99440500	-0.32886600
C	1.96872700	0.08505300	-1.86926900
C	-2.75207100	0.43011600	0.60043000
C	2.82341500	0.46849200	0.54848000
C	-0.13731400	2.49536000	1.31323700
C	-0.17781900	0.39272600	2.82121600
C	0.47668000	1.74261100	2.50044700
H	-1.25643300	0.53208600	2.99607600
H	0.26733500	-0.01066000	3.74289200
H	0.37988300	2.39217800	3.39324500
H	1.55013000	1.59388700	2.32373200
H	0.35646300	3.47231400	1.20451800
H	-1.20918500	2.67053800	1.49217400
O	-3.77972000	0.85186600	1.01753900
C	-1.98132900	0.06315900	-1.86256500
O	-2.43622800	0.26930900	-2.93276600
C	2.13283800	-1.94897000	-0.31350700
N	2.67619800	-3.00106100	-0.36444300
O	2.40731900	0.28343900	-2.94773400
H	0.00250500	-0.85789000	-1.03837500

Complex CN-C2

Fe	1.47354000	-0.59695200	0.02889100
Fe	-1.58481200	0.24591500	-0.00278200
S	0.19524100	0.68070800	1.49296900
S	0.18655800	0.57943600	-1.57878900
N	2.23056400	-2.69248800	-2.11194400
O	-1.64827300	-2.69694300	0.04271300
C	1.90409800	-1.87942600	-1.31150500
C	-2.78339400	0.34694400	1.22613200
C	3.04523100	0.18019000	0.07730300
C	-1.47078000	-1.53641500	0.00084800
C	0.73355200	2.43862900	1.21405700
C	0.75539900	2.33769200	-1.39309300
C	0.33291400	3.08159000	-0.11976100
H	1.85092000	2.30178600	-1.47527700
H	0.36146500	2.88107900	-2.26434600
H	0.78001300	4.09501100	-0.15337600
H	-0.75886400	3.20317600	-0.13572900
H	0.30417900	3.02704900	2.03859200
H	1.82652200	2.42789500	1.32852000
O	4.16512600	0.57056200	0.17182000
C	1.81095100	-1.78167100	1.25809700
O	2.12129800	-2.56773600	2.08701200
C	-2.88171300	0.23774100	-1.39296300
N	-3.72780400	0.27777100	-2.22213400
O	-3.60592600	0.47870100	2.06313200
H	-1.93488600	1.74156600	-0.05661700

Complex CN-C3

Fe	1.54878700	-0.64565800	0.05002300
Fe	-1.45542900	0.25839800	-0.00275200
S	0.30978500	0.66978500	1.51525400
S	0.30409100	0.56015000	-1.58513500
N	2.17466600	-2.77503500	-2.09669800
O	-1.66589400	-2.67982000	0.00686100
C	1.89748100	-1.94776300	-1.29245900
C	-2.64894900	0.41384400	1.22588300
C	3.19961200	-0.05045100	0.14407500
C	-1.44595700	-1.52638300	-0.01511800
C	0.69374400	2.46218400	1.21191300
C	0.67375100	2.36812400	-1.39638300
C	1.40489600	2.77702700	-0.11016700
H	1.30185200	2.64042600	-2.25782400
H	-0.28966500	2.88753100	-1.48398300
H	2.39777800	2.30709900	-0.10062800
H	1.56945300	3.87248700	-0.14811900
H	-0.26533800	2.99317100	1.27663400
H	1.33429000	2.78101400	2.04792000
O	4.35782300	0.18755000	0.27373300
C	1.73503500	-1.88058300	1.26173800
O	1.95121600	-2.71125100	2.07624300
C	-2.74509900	0.34209900	-1.39601700
N	-3.58567100	0.44844300	-2.22510000
O	-3.46361000	0.58578900	2.06330600
H	-1.67387500	1.78360700	-0.05290900

Complex CN-C4

Fe	1.37590300	-0.54442100	0.00239200
Fe	-1.53983200	0.39571500	-0.15447500
S	0.15541000	0.81851500	1.44468900
S	0.28932100	0.72470200	-1.67782300
N	2.02814200	-2.73044600	-2.08267200
O	-1.69715900	-2.50036500	0.28907800
C	1.73727000	-1.88012200	-1.30783900
C	2.99614900	0.12409200	0.07219200
C	-1.46086300	-1.37266200	0.06036900
C	0.79867600	2.53862300	1.16665200
C	0.95312000	2.43978600	-1.43057600
C	0.47398100	3.19103300	-0.18335800
H	2.04900500	2.34802900	-1.42551600
H	0.66209400	3.01418700	-2.32258000
H	0.94310300	4.19531100	-0.18879400
H	-0.60916200	3.34797100	-0.25791000
H	0.38231000	3.15946600	1.97397300
H	1.88698100	2.47197300	1.30778700
O	4.14174300	0.42770500	0.17664000
C	1.61207200	-1.73261400	1.25076200
O	1.85316600	-2.52627200	2.09488700
C	-2.73590300	0.10026300	-1.59417700
N	-3.53411400	-0.04547800	-2.45899200
C	-2.42879500	1.87482200	0.15629400
O	-3.18382300	2.73044300	0.47125700
H	-2.55242800	0.07910200	0.91874300

Complex CN-C5

Fe	1.43930300	-0.57213500	0.00977500
Fe	-1.43970700	0.39819500	-0.22860300
S	0.21529700	0.82728300	1.41755200
S	0.40018200	0.66487300	-1.73061400
N	1.99451900	-2.79295900	-2.05467800
O	-1.69301700	-2.46624500	0.33215600
C	1.75153300	-1.93747300	-1.28653100
C	3.11043300	-0.03067200	0.12809900
C	-1.44913300	-1.35985700	0.06971700
C	0.72396600	2.58200700	1.08903900
C	0.80808900	2.45948200	-1.49762200
C	1.51438700	2.82014100	-0.19381700
H	1.45624200	2.73358900	-2.33971900
H	-0.13317600	3.01313700	-1.59453200
H	2.45568500	2.26523200	-0.13860600
H	1.77212800	3.89508900	-0.23022100
H	-0.19274900	3.18219700	1.09445200
H	1.33418300	2.88150200	1.95039800
O	4.25900300	0.19238000	0.26604700
C	1.55490200	-1.77203800	1.27307200
O	1.71889200	-2.56205300	2.12329400
C	-2.63710900	0.10787200	-1.67488900
N	-3.42034900	-0.03205000	-2.53915700
C	-2.18433800	1.97550800	-0.03733900
O	-2.81887200	2.93528200	0.18467500
H	-2.52194600	0.19858100	0.80624500

Complex CN-C6

Fe	-1.29724100	-0.17476100	-0.18242900
Fe	1.26959200	-0.10582600	-0.21837100
S	-0.09369500	1.78600700	-0.19991900
S	0.02668800	-0.79054400	1.59834200
N	-1.99846500	-3.16912100	-0.50583800
O	3.89427600	0.89206300	0.58371800
C	-1.71005700	-2.02545800	-0.36538600
C	1.55859900	0.03246600	-1.92583700
C	-2.81235800	0.31912500	0.49831500
C	2.82938000	0.44110400	0.29934200
C	-0.17814200	2.60075800	1.46622900
C	-0.03104600	0.49870300	2.93332700
C	0.56209100	1.86860400	2.59004700
H	-1.08689100	0.60090500	3.22494700
H	0.51570100	0.06219900	3.78247300
H	0.53054200	2.49998300	3.50108800
H	1.62015200	1.75181900	2.31548200
H	0.25458100	3.60329100	1.32872300
H	-1.24223200	2.71963900	1.71919000
O	-3.86639200	0.69906500	0.89958200
C	-1.64618600	0.00937800	-1.87727900
O	-1.96733000	0.11964000	-3.01010200
C	1.69138700	-2.10900500	-0.44348200
N	2.79804200	-2.63858400	-0.35538000
O	1.83182200	0.12645700	-3.07497200
H	0.72890400	-2.61858900	-0.64267200

Complex CN-C7

Fe	-1.29104700	-0.13458400	-0.28355400
Fe	1.27072900	-0.13438000	-0.13052400
S	0.00004200	1.79256900	-0.19671700
S	-0.12774400	-0.79776500	1.58787700
N	-2.74768200	-2.71424200	-0.53283700
O	3.90211300	0.77194400	0.75757200
C	-1.65896200	-2.14088100	-0.55921000
C	1.53500400	-0.00202300	-1.84264300
C	-2.82760200	0.38532000	0.31437900
C	2.81434700	0.39225500	0.45856000
C	-0.09904400	2.61455900	1.46597900
C	-0.27805600	0.49957900	2.91057600
C	0.46527400	1.81138900	2.64131100
H	-1.35093700	0.68750300	3.06097900
H	0.11851300	0.03183900	3.82390200
H	0.41125400	2.44089200	3.55250000
H	1.52773500	1.59521000	2.45993000
H	0.44854600	3.56429600	1.36842700
H	-1.16027900	2.84869400	1.63806900
O	-3.86343900	0.83359700	0.69419900
C	-1.64018400	0.05147100	-1.97822400
O	-1.96331600	0.17787900	-3.11130300
C	1.75633200	-1.96717500	-0.26024000
N	2.08765200	-3.10219400	-0.37230300
O	1.80513700	0.07120000	-2.99164100
H	-0.67304000	-2.61977400	-0.71978100

Complex CN-C8

Fe	-1.23320900	-0.29337400	-0.28399200
Fe	1.35214500	-0.13875900	-0.18438000
S	-0.02341700	1.67891300	-0.21914100
S	0.02003000	-0.91065200	1.56485300
N	-2.51580800	-3.10394100	-0.52766600
O	3.90632700	0.97766800	0.68387800
C	-1.95403800	-2.06379700	-0.43069700
C	1.64540600	0.03715800	-1.88981900
C	-2.76575300	0.51299800	0.44648100
C	2.85776000	0.50196100	0.39018300
C	-0.11572100	2.50711700	1.43498800
C	-0.18046500	0.39016900	2.87984700
C	0.50156500	1.73174500	2.60108500
H	-1.25830000	0.53446100	3.03624200
H	0.24280600	-0.06056300	3.78944900
H	0.42607100	2.35921600	3.51146100
H	1.57153300	1.56223800	2.41266000
H	0.40184000	3.46945200	1.30734400
H	-1.18499000	2.69270100	1.60231300
O	-3.06993700	1.63898500	0.86945100
C	-1.71799700	0.06751000	-1.90747800
O	-2.07260500	0.31541900	-3.00769500
C	1.98735900	-1.93224800	-0.32520800
N	2.48124100	-3.00583600	-0.42719400
O	1.92162700	0.14470000	-3.03272000
H	-3.57494000	-0.27063700	0.50068200

Complex CN-C9

Fe	-1.46841700	-0.21208300	-0.24681200
Fe	1.44463300	-0.21109700	-0.17822800
S	0.00104800	1.58744600	-0.31876700
S	-0.09469100	-0.97404300	1.50022200
N	-2.70225500	-3.03286700	-0.50951300
O	3.79840300	1.18101200	0.71567200
C	-2.16897400	-1.97832900	-0.40449900
C	1.87072800	-0.06428000	-1.96620300
C	-2.81226900	0.49669400	0.60094100
C	2.81646700	0.60159500	0.35969200
C	-0.11590500	2.44367600	1.32385200
C	-0.24343600	0.33404900	2.81203800
C	0.44395700	1.67160400	2.52344600
H	-1.31546400	0.48528600	3.00339700
H	0.20271700	-0.11235600	3.71283700
H	0.33549300	2.31524300	3.41951900
H	1.51900800	1.50220900	2.37672800
H	0.43389200	3.38907200	1.20699200
H	-1.17847700	2.68002200	1.47688200
O	-3.77525200	0.96443600	1.11551700
C	-2.06967400	0.15150500	-1.84742700
O	-2.56148200	0.41086900	-2.88625500
C	2.34353100	-1.87725700	-0.15527900
N	2.99579400	-2.86995500	-0.14930300
O	0.96838200	-0.37729000	-2.76202000
H	2.86633200	0.22656500	-2.39154600

Complex CN-C10

Fe	-1.50388400	-0.14522900	-0.23486400
Fe	1.41640700	-0.20914200	-0.18582100
S	0.04310300	1.65530500	-0.33173200
S	-0.10570700	-0.90652800	1.50628900
N	-2.21459400	-2.98258900	-0.83641100
O	3.96213800	0.96004900	0.67929900
C	-1.88741400	-1.81534700	-0.57179000
C	1.77025500	-0.12711300	-1.88825500
C	-2.87627800	0.46519900	0.66977000
C	2.89214600	0.48618700	0.44644100
C	-0.09856100	2.50117300	1.31532300
C	-0.25146500	0.40236600	2.81256200
C	0.46351200	1.72492600	2.51185000
H	-1.32474100	0.57732600	2.98199800
H	0.17702900	-0.03930500	3.72564100
H	0.38826000	2.37334900	3.40824700
H	1.53127100	1.52583000	2.34322300
H	0.43649800	3.45823100	1.21553900
H	-1.16870300	2.71171800	1.46181000
O	-3.97872800	0.67279700	1.08306800
C	-2.03019000	0.27455300	-1.83438900
O	-2.43668400	0.55001300	-2.91215200
C	2.08638600	-1.99030200	-0.17511900
N	2.57487500	-3.07290500	-0.17685200
O	2.12924100	-0.06335100	-3.01660500
H	-3.04353800	-3.38151600	-0.38415200

Complex CN-C11

Fe	-1.40056700	-0.27343400	-0.20600500
Fe	1.53201400	-0.09492900	-0.17056100
S	-0.09938100	1.64302000	-0.33668400
S	0.09283600	-0.87712500	1.53316700
N	-2.47140100	-3.17399000	-0.19615200
O	4.05676200	0.60763700	1.11786600
C	-2.01601200	-2.07698000	-0.19595800
C	2.07912000	0.35721100	-1.75265000
C	-2.85131000	0.39760900	0.50265000
C	2.94224300	0.44904500	0.71473600
C	-0.22491000	2.50889100	1.30236900
C	-0.03741200	0.43915700	2.83843100
C	0.49157400	1.83119300	2.47480800
H	-1.10606800	0.49329700	3.09603600
H	0.51007600	0.06309400	3.71653100
H	0.38892100	2.48273400	3.36632300
H	1.56369900	1.75935700	2.24571300
H	0.19557200	3.51491000	1.14991300
H	-1.29961900	2.61116100	1.51436300
O	-3.91075300	0.86096900	0.79805000
C	-1.82260700	-0.17186900	-1.89048500
O	-2.22007200	-0.09447800	-3.00576400
C	1.84761900	-1.76860100	-0.56163600
N	2.14766600	-2.93421200	-0.86055400
O	2.49073200	0.65497300	-2.82194700
H	2.97739300	-3.36040100	-0.43484800

Complex CN-C12

Fe	-1.33827300	-0.11088500	-0.18779800
Fe	1.32025300	-0.13888500	-0.19447300
S	0.01492800	1.79137600	-0.20870700
S	-0.03258700	-0.83654800	1.55203100
N	-2.96684000	-2.66970100	-0.28018500
O	3.91287400	0.91824400	0.63618500
C	-2.14945100	-1.79967900	-0.27336000
C	1.57665000	0.01503500	-1.90856500
C	-2.88774500	0.39227700	0.43122000
C	2.84109200	0.47586700	0.36880800
C	-0.08855000	2.57797300	1.46684600
C	-0.11859100	0.44481400	2.89644100
C	0.55834000	1.78763600	2.60753600
H	-1.18644700	0.59407900	3.11432600
H	0.35294000	-0.02336100	3.77370600
H	0.51477700	2.40476900	3.52754000
H	1.62127400	1.62023600	2.38191700
H	0.39207500	3.56353700	1.37422400
H	-1.16122800	2.72764000	1.66372200
O	-4.11474400	-0.10492200	0.49463900
C	-1.61791100	0.03043200	-1.90736800
O	-1.91079900	0.09439900	-3.04933600
C	1.92656800	-1.93972400	-0.31434600
N	2.39459200	-3.02664400	-0.40310400
O	1.85473400	0.11649600	-3.05356300
H	-4.04218300	-1.07596700	0.20434500

Complex CN-C13

Fe	-1.29600200	-0.14941200	-0.27007700
Fe	1.34447000	0.00559000	-0.12225100
S	-0.12167300	1.84991000	-0.18720100
S	-0.01883400	-0.79825200	1.54421900
N	-2.15461000	-3.09483500	-0.66326800
O	4.16773800	0.20952100	0.33951400
C	-1.76303600	-1.98615100	-0.50387900
C	1.46345400	0.04930300	-1.86241300
C	-2.80240800	0.34431600	0.42613100
C	2.90637400	0.61928500	0.34968600
C	-0.29880800	2.61097000	1.49472300
C	-0.19399000	0.46742000	2.89711800
C	0.40829900	1.84739900	2.61850500
H	-1.26919500	0.55172200	3.11653100
H	0.29900700	0.01878100	3.77237100
H	0.34975800	2.45046900	3.54698200
H	1.47175700	1.73178300	2.36038900
H	0.12623900	3.62198000	1.40566900
H	-1.37462100	2.70719300	1.70668900
O	-3.85342500	0.72228400	0.83461500
C	-1.67387300	0.11392900	-1.94970600
O	-2.02535100	0.28871800	-3.06493200
C	2.26207600	-1.62436900	-0.15036000
N	3.12321500	-2.45104100	-0.15647500
O	1.67234900	0.05399600	-3.02510800
H	4.14133900	-0.78200400	0.11968700

Complex CN-C14

Fe	-1.31249200	-0.14158000	-0.23802700
Fe	1.25377000	0.04221700	-0.31136700
S	-0.19828400	1.87199500	-0.18035600
S	0.03766000	-0.80950700	1.50493400
N	-2.22944900	-3.07560700	-0.58724700
O	3.86927700	1.06401600	0.48677100
C	-1.80766000	-1.97530900	-0.45056800
C	1.43887300	0.24770100	-2.06144400
C	-2.80547900	0.33642800	0.48622700
C	2.78926400	0.64290800	0.22026500
C	-0.26219900	2.59645000	1.52781000
C	-0.07887600	0.42247100	2.89068500
C	0.48988900	1.81730100	2.61212200
H	-1.14031700	0.49048900	3.17279100
H	0.46561300	-0.03356500	3.73147700
H	0.45305600	2.40526800	3.55173900
H	1.54804200	1.72636900	2.32832500
H	0.16638300	3.60579000	1.43437700
H	-1.32280800	2.70346400	1.79914800
O	-3.83481900	0.72752400	0.93451900
C	-1.71211000	0.10284200	-1.91959900
O	-2.07075300	0.24677600	-3.03508300
C	1.90505800	-1.67454700	-0.70534200
N	2.43815500	-2.61327900	-1.21277800
O	1.93166700	-0.43783600	-3.07020400
H	2.20617200	-1.34651600	-2.70682200

Complex CN-C15

Fe	-1.26433500	0.02459000	-0.38067100
Fe	1.29107100	-0.10899600	-0.17118100
S	0.12086300	1.88023800	-0.18633300
S	-0.14770100	-0.79907900	1.50500100
N	-2.39943000	-2.61589100	-1.38921900
O	3.89159400	0.82662900	0.75656800
C	-1.86690000	-1.69744900	-0.84464700
C	1.59419100	0.04459200	-1.87957700
C	-2.77076700	0.58327600	0.26294600
C	2.82122600	0.41971100	0.43578900
C	0.01249800	2.62019000	1.51410100
C	-0.25893400	0.45457600	2.87567300
C	0.53009700	1.75034200	2.66380600
H	-1.32576200	0.67572000	3.02788600
H	0.11353300	-0.05794300	3.77557200
H	0.48294800	2.34652000	3.59743200
H	1.58753900	1.50589900	2.49022100
H	0.59074800	3.55537800	1.47140300
H	-1.04397500	2.88102700	1.67807000
O	-3.81543600	0.99100500	0.65718100
C	-1.56830500	0.31899300	-2.10733700
O	-2.11744200	-0.32613600	-3.11309300
C	1.85147600	-1.92845400	-0.28586900
N	2.30246500	-3.02298300	-0.36282600
O	1.89492300	0.12261800	-3.01844600
H	-2.34439500	-1.26157800	-2.77855600

Complex CN-D1

Fe	1.38585300	-0.33201000	0.00881700
Fe	-1.36899500	-0.34729500	-0.00830400
S	0.04910800	0.72821100	1.61624600
S	-0.02541800	0.69832000	-1.61123200
N	2.52446900	-2.11339800	-2.24589500
O	-3.90258500	1.14228300	-0.07166600
C	2.05429900	-1.43163300	-1.39738500
C	-1.98973900	-1.45203600	1.41319500
C	2.75839900	0.77393700	-0.02658700
C	-2.82213500	0.65598200	0.00542700
C	0.18915300	2.54178700	1.27205500
C	0.06509700	2.52508800	-1.31228700
C	-0.51664200	3.04726900	0.00711100
H	1.13064400	2.79356500	-1.38358800
H	-0.46212200	2.99775300	-2.15416000
H	-0.44094100	4.15299400	-0.00638600
H	-1.58442900	2.79745200	0.06132200
H	-0.24053300	3.04619500	2.15030800
H	1.26205000	2.78487800	1.22681400
O	3.78026300	1.37546000	0.02700200
C	2.04552700	-1.42435500	1.18129500
O	2.55667400	-2.14864500	1.95928200
C	-1.96664100	-1.49608600	-1.16220800
O	-2.45072900	-2.25246800	-1.92704400
N	-2.42321300	-2.13889500	2.27691900
H	0.03628600	-1.34212100	0.01067900
H	2.97517966	1.67157008	0.00000004
C	2.61852524	2.68038008	0.00000004
H	2.97519808	3.18477827	0.87365154
H	2.97519808	3.18477827	-0.87365147
H	1.54852524	2.68039327	0.00000004

Complex CN-D2

Fe	-1.46592900	-0.29065600	0.01243800
Fe	1.62727500	-0.10350900	0.01334800
S	-0.01247000	0.66327200	-1.53861900
S	0.01574800	0.69862100	1.55760000
N	-2.49641300	-2.12974100	2.27426100
O	3.65855000	-0.41806000	2.05835900
C	-2.06678800	-1.42457200	1.42167600
C	2.86960600	-0.37938300	-1.39799800
C	-2.86707900	0.76401500	-0.03374400
C	2.82139900	-0.32617500	1.23180900
C	-0.18322900	2.49896500	-1.32123500
C	-0.15165300	2.52931000	1.28069600
C	0.39690800	3.09680400	-0.03433300
H	-1.22755400	2.73768400	1.37096400
H	0.36452300	3.01573100	2.12162600
H	0.19332000	4.18619800	-0.04378500
H	1.48776700	2.96520600	-0.04854800
H	0.31882300	2.95668400	-2.18611500
H	-1.26019200	2.70729100	-1.39405300
O	-3.89383700	1.35694100	-0.13402400
C	-2.05592200	-1.44053200	-1.15272400
O	-2.54063900	-2.18863400	-1.93035700
C	1.10435600	-1.81287400	-0.04472300
O	1.02819700	-2.98470300	-0.07832600
N	3.68630000	-0.51838400	-2.24610800
H	2.32292100	1.26837300	-0.01300700

Complex CN-D3

Fe	-1.46373200	-0.28695100	0.01013800
Fe	1.63525600	-0.07696400	0.01402700
S	-0.00094000	0.64711300	-1.54971800
S	0.02066000	0.71247000	1.56348700
N	-2.40643900	-2.14824400	2.28804400
O	3.67448900	-0.25059000	2.06865900
C	-2.01312300	-1.43121100	1.42793800
C	2.89645200	-0.27630200	-1.39223800
C	-2.95171800	0.64582300	-0.06536600
C	2.83639600	-0.21927400	1.23844600
C	0.03363000	2.49094500	-1.34557200
C	0.07408800	2.54382000	1.26289300
C	-0.56985800	3.03057300	-0.04215500
H	-0.45789500	3.00070400	2.11098600
H	1.13150300	2.83772800	1.30897400
H	-1.64119300	2.78883300	-0.01954900
H	-0.49009100	4.13575200	-0.06635200
H	1.08818800	2.78087200	-1.44361000
H	-0.52900600	2.90556300	-2.19552600
O	-4.03968200	1.11658000	-0.16439100
C	-1.96069700	-1.50259800	-1.13166300
O	-2.39008900	-2.30096000	-1.89091200
C	1.21659500	-1.81157800	-0.03587500
O	1.17094400	-2.98488300	-0.06066400
N	3.72442300	-0.36035400	-2.23680000
H	2.20155700	1.35684600	-0.02221400

Complex CN-D4

Fe	1.32660000	-0.09298000	0.02344600
Fe	-1.40001000	-0.53270600	-0.25927900
S	-0.30917300	0.39693400	1.63090700
S	-0.10426600	0.96542600	-1.54602300
N	3.07375900	-1.05864000	-2.33873200
O	-4.01749900	0.59532700	-0.93419000
C	2.35908400	-0.70934500	-1.45909000
C	-2.32680500	-1.73366800	0.87120700
C	2.46087900	1.20117800	0.38661500
C	-2.94239900	0.25361100	-0.57470700
C	-0.47927200	2.24279600	1.63035500
C	-0.34678900	2.69301400	-0.91763700
C	-1.13409900	2.85847500	0.38880600
H	0.66022200	3.12392600	-0.80654300
H	-0.86520600	3.23707600	-1.72096000
H	-1.25522500	3.94507600	0.57217200
H	-2.14122400	2.43829200	0.26604100
H	-1.08465700	2.48570100	2.51630000
H	0.52671900	2.66400400	1.77783800
O	3.34978600	1.92572500	0.69345100
C	2.08755100	-1.27274500	1.05146600
O	2.69604400	-2.01029700	1.74459400
C	-0.24036200	-1.87966800	-0.59353900
O	0.12212600	-2.94649000	-0.94159600
N	-2.96349600	-2.46816900	1.55161200
H	-1.91918000	-1.17235100	-1.52040800

Complex CN-D5

Fe	-1.46577000	-0.11008100	-0.04904700
Fe	1.55172500	-0.47240000	0.25432600
S	0.25124300	0.39845300	-1.54442800
S	0.02662400	0.80555200	1.55603000
N	-2.96311600	-1.45360400	2.29577900
O	3.85641900	1.23662100	0.81299300
C	-2.35999500	-0.94096700	1.41158400
C	2.66312500	-1.39408500	-0.97275100
C	-2.75358900	1.03187100	-0.39679300
C	2.88231200	0.63800700	0.50839300
C	0.60591000	2.22121500	-1.48512900
C	0.38701200	2.56411900	1.08189900
C	-0.04047200	2.99218300	-0.32795600
H	-0.15180400	3.17875000	1.81803500
H	1.46393200	2.73245200	1.22473700
H	-1.13263400	2.91244500	-0.41130600
H	0.21574200	4.06396700	-0.44700600
H	1.70043900	2.32269800	-1.46032400
H	0.24409800	2.63493700	-2.43824300
O	-3.72423100	1.65704500	-0.68402100
C	-2.05312200	-1.37546300	-1.09267300
O	-2.55022700	-2.18676700	-1.79467000
C	0.78877700	-2.03646000	0.62774800
O	0.52711000	-3.12606700	0.97537300
N	3.40789400	-1.95125500	-1.70910300
H	2.27374400	-0.97999700	1.48118100

Complex CN-D6

Fe	-1.47858100	-0.23005100	0.02365900
Fe	1.47604900	-0.31504600	0.00003100
S	-0.01644600	0.66383500	-1.54550300
S	0.01091900	0.70542000	1.59571100
N	-2.55594600	-2.07054000	2.26306900
O	4.04851600	0.95846700	-0.53905000
C	-2.10200600	-1.36694500	1.42047500
C	1.53383900	-1.69842300	-1.06253900
C	-2.83880200	0.87641800	-0.00394800
C	2.93216400	0.61529600	-0.28049600
C	0.00264700	2.50643600	-1.30286000
C	0.00019900	2.53748900	1.28303000
C	0.65970700	3.01588000	-0.01488300
H	-1.05689000	2.84282300	1.31006500
H	0.51478100	2.99869800	2.14011900
H	0.63107200	4.12434700	-0.03101200
H	1.71696600	2.71739900	-0.00843500
H	0.53023700	2.92798000	-2.17246200
H	-1.04989700	2.82384500	-1.34770100
O	-3.86832200	1.47223400	-0.10054400
C	-2.08984000	-1.37925200	-1.12814500
O	-2.60194500	-2.11998600	-1.89862500
C	2.12039400	-1.28534500	1.28566900
O	2.60733900	-1.94596100	2.13699200
N	1.64668100	-2.68718600	-1.80829900
H	2.43830300	-2.71391100	-2.46123000

Complex CN-D7

Fe	1.44917800	-0.30125300	-0.02005700
Fe	-1.39353600	-0.20734700	0.04187000
S	0.10511100	0.77934600	1.60342900
S	-0.00480000	0.74050900	-1.56546100
N	1.43316800	-2.62900800	-1.89325000
O	-3.97617300	1.16980300	-0.17810300
C	1.40141700	-1.67598300	-1.09823100
C	-1.94950300	-1.32924400	1.47361600
C	2.88832500	0.67643600	-0.28998600
C	-2.87673700	0.73054500	-0.02922200
C	0.31682700	2.59049900	1.26087200
C	0.17055600	2.56996800	-1.31018800
C	-0.39070900	3.11636600	0.00673900
H	1.24483600	2.79556100	-1.39077600
H	-0.34826400	3.04367100	-2.15764800
H	-0.29565100	4.22105100	-0.01118600
H	-1.46284800	2.88142500	0.07097400
H	-0.07895200	3.11115900	2.14670900
H	1.39884400	2.78420200	1.19667600
O	3.99032200	1.05895800	-0.54400300
C	2.08271100	-1.32150600	1.23164100
O	2.56967600	-2.01350500	2.05828500
C	-1.80923900	-1.49355900	-1.04096500
O	-2.16020700	-2.36972400	-1.76368400
N	-2.36266500	-2.03414500	2.33594900
H	0.60601900	-3.23447200	-1.93386300

Complex CN-D8

Fe	1.27637000	-0.36270100	-0.01029900
Fe	-1.29254400	-0.32791500	-0.00661100
S	-0.02933700	0.74778600	1.57456200
S	0.01706900	0.77330300	-1.58382000
N	2.69440300	-1.97542400	-2.23296600
O	-3.90952700	0.80937600	-0.64250000
C	2.11317800	-1.36008200	-1.39986800
C	-1.93339600	-1.41286100	1.42002100
C	2.76806900	0.76212800	0.21029600
C	-2.84157500	0.38598400	-0.34225400
C	-0.00047000	2.58121300	1.29981900
C	0.05948000	2.59997300	-1.25787400
C	-0.66210900	3.06797600	0.00883700
H	1.12092400	2.87748300	-1.21081700
H	-0.39489300	3.06410400	-2.14529600
H	-0.66315500	4.17619200	0.01568600
H	-1.71232800	2.74219000	-0.01921900
H	-0.52080700	3.00269800	2.17254700
H	1.05592700	2.87823200	1.32177200
O	2.87310200	1.99287400	0.35078900
C	1.81362400	-1.50462500	1.17663100
O	2.21274200	-2.28910300	1.96530100
C	-1.42109100	-1.72154400	-1.05058700
O	-1.60709800	-2.67304000	-1.72103000
N	-2.39951600	-2.08632100	2.27887000
H	3.73889600	0.20010800	0.12116700

Complex CN-D9

Fe	-1.34519000	-0.20229700	0.06247200
Fe	1.21626300	-0.42669900	-0.13112200
S	-0.09227500	0.72327000	-1.66957200
S	0.13103900	0.82894600	1.46722000
N	-2.44950900	-1.72551900	2.51515600
O	3.36703200	1.07015400	1.12654800
C	-1.97875100	-1.14878200	1.59095500
C	1.80344900	-1.56426700	-1.55662000
C	-2.77447600	0.76393700	-0.12011900
C	2.85396100	0.41559200	0.20928000
C	-0.07563300	2.56389100	-1.41864000
C	0.30698700	2.64223800	1.12347500
C	0.80456000	3.05036100	-0.26606300
H	-0.68098100	3.08443200	1.32276700
H	1.01211200	3.00762000	1.88256500
H	0.85467600	4.15698800	-0.30194000
H	1.82734000	2.67590800	-0.40526000
H	0.29306600	2.97882400	-2.36836600
H	-1.11663900	2.89128900	-1.28498500
O	-3.76239200	1.39542500	-0.31109200
C	-1.79098600	-1.57721900	-0.91871800
O	-2.17734500	-2.49592400	-1.54910300
C	1.57310700	-1.67146900	1.01425400
O	1.84777500	-2.53528500	1.76831400
N	2.23735800	-2.25276700	-2.42032000
H	3.49351100	0.25072300	-0.71026700

Complex CN-D10

Fe	1.42678300	-0.35145100	0.06268600
Fe	-1.37196800	-0.23439900	-0.02338600
S	0.00489600	0.73115800	1.58317600
S	0.07869600	0.68695800	-1.59355800
N	3.01489000	-1.89102100	-2.08004500
O	-3.78425400	1.13825700	-0.93254000
C	2.36105700	-1.31136100	-1.27503100
C	-2.27543400	-1.10040800	1.41185200
C	2.75111800	0.63843100	0.39130600
C	-2.79612700	0.62318000	-0.52289500
C	0.20603200	2.55108500	1.25496900
C	0.19938300	2.52378000	-1.32425800
C	-0.43148100	3.06655400	-0.03839300
H	1.27052900	2.77020500	-1.35633700
H	-0.28582200	2.99047700	-2.19434600
H	-0.33774500	4.17096600	-0.05165600
H	-1.50566100	2.83380200	-0.03014900
H	-0.26164300	3.04835300	2.11707900
H	1.28134500	2.77672600	1.27275700
O	3.69058700	1.33305500	0.63651400
C	1.83332700	-1.67113500	1.28256600
O	0.98766300	-2.57800900	1.32599500
C	-1.59958300	-1.66416400	-1.00794800
O	-1.86286000	-2.61954600	-1.64399400
N	-2.92839100	-1.59848000	2.26812000
H	2.73202000	-1.74762800	1.94960200

Complex CN-D11

Fe	1.42859600	-0.21773700	0.01313100
Fe	-1.45824500	-0.38588500	-0.05629300
S	-0.08931700	0.60086500	1.61745700
S	-0.04745900	0.72330800	-1.53842500
N	3.04524300	-1.45301000	-2.30932400
O	-3.89808200	0.95430800	-0.78509100
C	2.37350600	-1.00510100	-1.43988300
C	-2.33303500	-1.41674500	1.26970100
C	2.70141500	0.91003400	0.37539500
C	-2.88175700	0.40951100	-0.47431700
C	-0.05964000	2.44547400	1.39810900
C	-0.09303700	2.54767800	-1.18549000
C	-0.74426700	2.97437800	0.13443200
H	0.94918100	2.89477000	-1.23186300
H	-0.64214600	2.99903400	-2.02463400
H	-0.73070000	4.08221200	0.17409300
H	-1.79815800	2.66485200	0.13840000
H	-0.57408100	2.84768900	2.28335600
H	0.98956200	2.77154700	1.43589400
O	3.61459600	1.61072700	0.66624100
C	1.95697500	-1.50719100	1.07159600
O	2.40467900	-2.34200400	1.77344900
C	-1.67396100	-1.77437600	-1.24277400
O	-0.72218400	-2.57085800	-1.24725600
N	-2.95280300	-2.04489200	2.06514000
H	-2.55070300	-1.98674800	-1.90758000

Complex CN-D12

Fe	1.28368400	-0.17130900	0.04632500
Fe	-1.27900500	-0.37594500	-0.03907900
S	-0.13744100	0.78509900	1.60169400
S	-0.02272200	0.84661200	-1.54453900
N	3.12695600	-1.27348700	-2.00607900
O	-3.97283200	0.61274500	-0.58241500
C	1.99691100	-1.29990500	-1.50472400
C	-1.82726900	-1.55324300	1.35760400
C	2.69309800	0.79878300	0.30287200
C	-2.87357000	0.25480600	-0.30177600
C	-0.20875500	2.62368000	1.33003700
C	-0.16101700	2.67356800	-1.23729300
C	-0.89773200	3.08187600	0.04153600
H	0.86639700	3.06567100	-1.23252300
H	-0.68209800	3.08611700	-2.11412500
H	-0.97819400	4.18757200	0.05779700
H	-1.92206100	2.68371300	0.01611900
H	-0.74832900	3.02875800	2.19939400
H	0.82500600	2.99788400	1.36671200
O	3.62786800	1.47420000	0.59759700
C	1.70718800	-1.51172600	1.07464400
O	2.07658000	-2.41229100	1.74605700
C	-1.29622600	-1.73538700	-1.11253900
O	-1.36478100	-2.67824400	-1.82581200
N	-2.24733400	-2.28722800	2.19034500
H	1.19242500	-1.96947300	-1.87166700

Complex CN-D13

Fe	1.33399200	-0.25474900	0.03830800
Fe	-1.23883400	-0.28754300	-0.04014000
S	-0.01142800	0.83832800	1.55325200
S	0.10021200	0.80068000	-1.59014000
N	2.46411600	-2.10449100	-2.17247900
O	-3.84363000	0.89140600	-0.63084900
C	1.97894800	-1.40295800	-1.34723100
C	-1.78426300	-1.49883700	1.51099000
C	2.80112700	0.65175500	0.21327600
C	-2.79309700	0.42697900	-0.31793300
C	0.03167400	2.67125800	1.25477900
C	0.12149300	2.63817500	-1.31095300
C	-0.60430100	3.13752900	-0.05765400
H	1.17769400	2.94573100	-1.29205500
H	-0.34556900	3.07720300	-2.20540400
H	-0.60861500	4.24613200	-0.07105700
H	-1.65254000	2.80838000	-0.08869600
H	-0.50208400	3.11822900	2.10672600
H	1.08492400	2.98340100	1.30825300
O	3.80708400	1.24901400	0.42815500
C	1.62148300	-1.55076600	1.15835700
O	1.87556700	-2.43784600	1.90048900
C	-1.44362000	-1.68425200	-1.05592200
O	-1.66791500	-2.63720800	-1.71817200
N	-2.90704800	-1.62544500	2.01285800
H	-0.89345700	-2.05025700	1.87420700

Complex CN-D14

Fe	1.32367800	-0.34895900	0.11594700
Fe	-1.28221600	-0.11727700	-0.16082500
S	-0.05412900	0.89108700	1.50256500
S	0.31427800	0.77347200	-1.66201700
N	2.61500500	-2.21176600	-1.97400000
O	-4.10756800	0.33845500	-0.39190000
C	2.08209900	-1.51311700	-1.19307200
C	-2.32385400	-0.93903600	1.15705700
C	2.81208900	0.47703900	0.46197500
C	-2.82011100	0.54316700	-0.62166300
C	0.21800900	2.68937400	1.13088000
C	0.46032900	2.60032300	-1.39846100
C	-0.32254200	3.15510400	-0.21460000
H	1.52935300	2.82413100	-1.28520800
H	0.10698400	3.05957400	-2.33047600
H	-0.27815300	4.25982600	-0.24977100
H	-1.37478200	2.85683800	-0.30643900
H	-0.27703900	3.22952000	1.94761500
H	1.29765700	2.87350600	1.19583400
O	3.81291000	1.00899800	0.78190400
C	1.38979800	-1.63444600	1.29983600
O	1.50024500	-2.48632600	2.09333300
C	-1.15763700	-1.65496000	-0.99274800
O	-1.20586700	-2.69051700	-1.52942800
N	-3.20330600	-1.35874800	1.82741200
H	-4.15315500	-0.32617300	0.36612600

Complex CN-D15

Fe	1.28481600	-0.07568500	0.16290400
Fe	-1.31943700	-0.39619900	-0.12629400
S	-0.32324000	0.66263700	1.70850000
S	0.01059400	0.95611100	-1.44448800
N	3.24824400	-1.10375900	-1.90394400
O	-3.95957000	0.56674800	-0.89708300
C	2.34480900	-0.79926700	-1.20349900
C	-1.97999600	-1.66042000	1.13773400
C	2.72144500	0.84394900	0.49843500
C	-2.89543500	0.21903300	-0.52729200
C	-0.31652900	2.50498000	1.52357900
C	-0.22324300	2.74228300	-1.00726500
C	-1.00485700	3.02476400	0.26834900
H	0.78649800	3.16547600	-0.93037700
H	-0.72768600	3.19526000	-1.87033700
H	-1.13244900	4.11994800	0.35733100
H	-2.00518800	2.58279600	0.19250500
H	-0.81665900	2.89678300	2.41828200
H	0.73650000	2.81656300	1.53810700
O	4.00132800	0.82092300	0.17745600
C	1.50784800	-1.55484900	1.07895500
O	1.74041200	-2.53818100	1.66238900
C	-1.15317800	-1.68236900	-1.29588500
O	-1.11727700	-2.55002800	-2.07943300
N	-2.44171600	-2.43369300	1.89383100
H	4.09147100	0.13003400	-0.55607900

Complex CN-D16

Fe	-1.28799600	-0.46595500	-0.00429900
Fe	1.24777000	-0.01455100	0.07048000
S	-0.20014600	0.76775200	-1.63225300
S	-0.29164300	0.90277700	1.54763400
N	-2.10313900	-2.24826700	2.37709700
O	3.59785300	1.64798700	0.50164900
C	-1.74482300	-1.57431900	1.48280300
C	2.11980900	-1.08360500	-1.20873500
C	-2.90611600	0.08783800	-0.27855400
C	2.62450400	1.01443100	0.30027800
C	-0.65546000	2.54904000	-1.38677900
C	-0.57071400	2.69510400	1.16007900
C	-0.02042700	3.20532400	-0.16735900
H	-1.65629000	2.84961600	1.20183200
H	-0.11628900	3.24868500	1.99155400
H	-0.19719100	4.29646600	-0.21819200
H	1.06410100	3.04880200	-0.19821300
H	-0.33452800	3.06364600	-2.30147400
H	-1.74920400	2.60277200	-1.32238800
O	-4.00197300	0.47569100	-0.47147800
C	-1.21908700	-1.89752600	-1.00877400
O	-1.22785500	-2.87076300	-1.65303700
C	1.72028900	-1.33622900	1.15262500
O	2.62106000	-2.29011300	1.11534600
N	2.81096000	-1.84632300	-1.79066200
H	2.94866400	-2.34595700	0.16169400

Complex CN-D17

Fe	1.28847000	0.14034100	0.07542000
Fe	-1.18286300	-0.56873000	0.01447000
S	-0.32326100	0.88072500	1.58847200
S	-0.22822300	0.79993300	-1.59930300
N	2.96884100	-1.59480500	-1.77781900
O	-3.98962600	-0.20376100	-0.65959500
C	2.23615000	-0.86998800	-1.19860800
C	-1.52886500	-1.74236100	1.47889400
C	2.48260200	1.39155300	0.24055500
C	-2.86236100	-0.32263400	-0.33881400
C	-0.71421400	2.64855100	1.17429100
C	-0.77110500	2.55965800	-1.37604600
C	-1.53529700	2.85712800	-0.09237400
H	0.13820100	3.17238100	-1.42472400
H	-1.39360300	2.79993700	-2.24778100
H	-1.87263600	3.91002300	-0.12675200
H	-2.43175100	2.22834800	-0.04744900
H	-1.26243600	3.03632800	2.04205900
H	0.23991000	3.18486000	1.10130600
O	3.31377400	2.20855900	0.41074600
C	1.93299400	-1.09363900	1.17566200
O	2.75829200	-2.10583100	1.10369100
C	-0.84520200	-1.96406900	-0.97874200
O	-0.68669400	-2.92444400	-1.62337900
N	-1.81318000	-2.47214600	2.35548500
H	3.03648900	-2.19698700	0.13523700
